# New Aluminum Complexes with an Asymmetric Amidine–Imine Ligand: Synthesis, Characterization, and Application in Catalysis

**DOI:** 10.3390/molecules30193842

**Published:** 2025-09-23

**Authors:** Fernando Gómez Zamorano, María José Rojas, Sonia Mallet-Ladeira, Alan R. Cabrera, Jordan Garo, Jean-Marc Sotiropoulos, Eddy Maerten, David Madec, René S. Rojas

**Affiliations:** 1 Laboratoire Hétérochimie Fondamentale et Appliquée (UMR 5069), Université de Toulouse, CNRS, 118 Route de Narbonne, 31062 Toulouse Cedex 09, France; fogomez@uc.cl (F.G.Z.); eddy.maerten@univ-tlse3.fr (E.M.); 2 Departamento de Química Inorgánica, Facultad de Química y de Farmacia, Pontificia Universidad Católica de Chile, Vicuña Mackenna 4860, Macul 7820436, Santiago, Chile; mjrojas20@uc.cl (M.J.R.); arcabrer@uc.cl (A.R.C.); 3 Institut de Chimie de Toulouse (UAR 2599), 118 Route de Narbonne, 31062 Toulouse Cedex 09, France; sonia.ladeira@univ-tlse3.fr; 4 CNRS, IPREM, Universite de Pau et des Pays de l’Adour, Technopôle Hélioparc, 2 Avenue du Président Angot, 64053 Pau Cedex 09, France; jordan.garo.p@gmail.com (J.G.); jean-marc.sotiro@univ-pau.fr (J.-M.S.)

**Keywords:** asymmetric amidine–imine ligand, aluminum complexes, catalysis

## Abstract

In this work, a new asymmetric amidine–imine ligand, using 1,8-diaminonaphthalene as a rigid platform, was synthesized and characterized, and its ability to form complexes with aluminum(III) was investigated. Several aluminum complexes were synthesized and characterized in solution and in the solid state. The synthesis of a dihalogenated aluminum(III) complex (**AlI_2_L**) using a reducing agent revealed an atypical pathway, which was investigated using Density Functional Theory (DFT). The dimethylated aluminum complex **AlMe_2_L** and the dihalogenated aluminum complex **AlI_2_L** were evaluated as catalysts for the transformation of CO_2_ and epoxides into cyclic carbonates in the presence of Bu_4_NI as a co-catalyst or in a single-component system, respectively. **AlMe_2_L/**Bu_4_NI appeared to be the most efficient system under 1 bar of CO_2_ at 90 °C.

## 1. Introduction

Coordination chemistry has experienced significant growth in recent decades due to the need to develop new materials and catalysts with applications in various scientific and technological fields, such as homogeneous catalysis, functional material design, and sustainable chemistry, among others [1,2,3]. In this context, ligands play a fundamental role, as their nature and design can decisively influence the reactivity and stability of the metal complexes they form.

**Figure 1:**
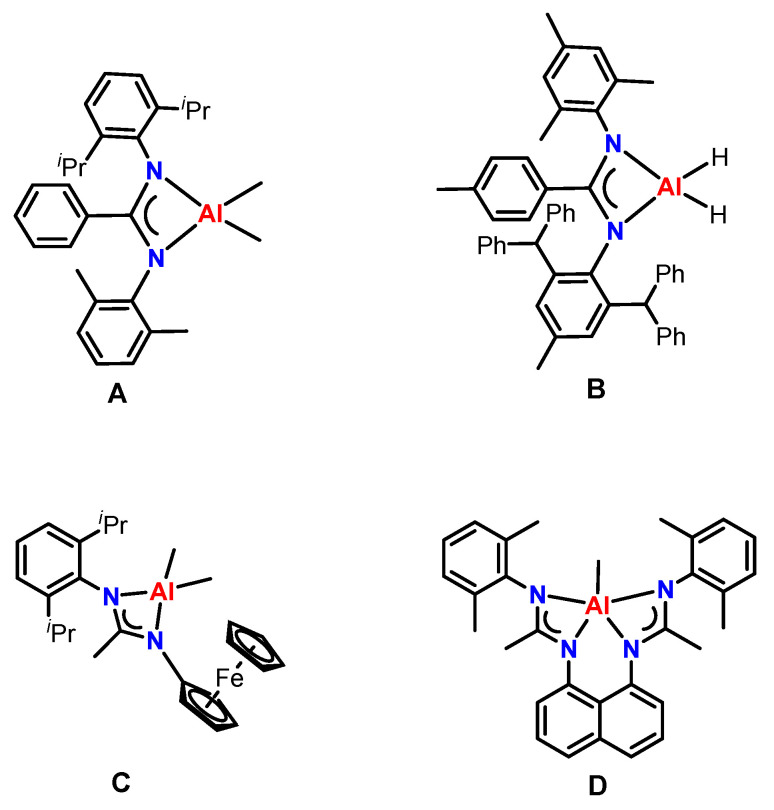
Aluminum complexes with amidine ligands applied in several catalytic systems [11,12,13,14].

Among the different types of ligands, amidine-based systems have attracted special attention due to their structural versatility, high basicity, affinity for Lewis acids, ability to stabilize metals in different oxidation states, and potential applications in catalysis [4,5]. For instance, these ligands have been utilized in the stabilization of transition metal complexes such as chromium, copper, silver, and titanium, which have demonstrated activity in the polymerization of ethylene, CS_2_ activation reactions, and olefin polymerization [6,7,8,9]. On the other hand, aluminum is a metal of great interest in coordination chemistry due to its abundance, low cost, and versatility in catalytic processes [10]. Aluminum complexes with amidinate ligands have proven to be efficient in processes such as ring-opening polymerization (ROP) of *rac*-lactide [11] (Figure 1A), hydroboration of terminal alkyls [12] (Figure 1B), and the chemical transformation of carbon dioxide into value-added products like cyclic carbonates [13,14] (Figure 1C,D).

This latest application will be the selected benchmark reaction. Indeed, carbon dioxide valorization and activation remain a major challenge today. Because of its importance in the greenhouse effect, consequent research has been conducted to convert it into commercially viable products such as urea formation, syngas, formic acid, polycarbonates, and cyclic carbonates [13,15,16].

In this work, we report the synthesis and characterization of a non-symmetrical amidine–imine ligand that could lead to the formation of a potential chiral environment around a metal center and the ability of this ligand to form aluminum(III) complexes. The formation of a dihalogenated aluminum complex with a reducing agent was studied in detail. The application of the dimethylated and the dihalogenated aluminum complexes as catalysts for the conversion of CO_2_ and epoxides into cyclic carbonates was evaluated.

## 2. Results and Discussion

### 2.1. Synthesis and Characterization of Amidine–Imine Ligand

**Scheme 1:**
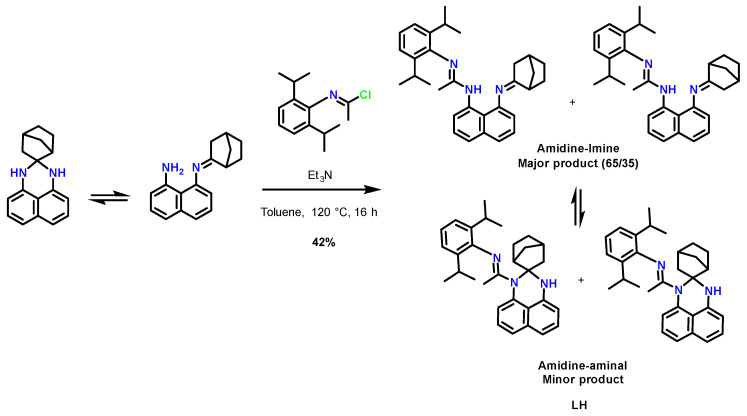
Amidine–imine and amidine–aminal equilibrium.

The asymmetric amidinate-imine ligand was synthesized starting from the aminal derived from 1,8-diaminonaphthalene and racemic norcamphor (see Section 3.2, and Supplementary Materials, Figures S1 and S2). It is interesting to note that the starting aminal in DMSO solution at room temperature exists only under the aminal form and that heating at 120 °C displaced the equilibrium to the corresponding amino-imine, as indicated by the total disappearance of the N-H signal of aminal by ^1^H NMR spectroscopy (see Supplementary Materials, Figure S3). Starting from this equilibrium, aminal reacts with *N*-imidoylphenyl chloride in the presence of triethylamine at reflux of toluene [17]. As shown in Scheme 1, a mixture of products (**LH**) was obtained in an imine–aminal equilibrium at room temperature, the main product corresponding to the amidine–imine under a 65/35 mixture of E/Z diastereomers (see Supplementary Materials, Figures S4–S6), due to the two stereogenic centers present in the bicycle, and traces of the amidine–aminal (Scheme 1). Several factors, such as temperature, pH, solvent choice, and reactions with bases or acids, can influence the predominance of one species over another [18]. In our case, at low temperature (−50 °C), ^1^H NMR in CDCl_3_ (See Supplementary Materials, Figure S7) show two signals around 11 ppm corresponding to the N-H of the E/Z diastereomers of the imine (73%), and two others signals at 5.3–5.4 ppm corresponding to the N-H of the diastereomers of the aminal (27%).

**Figure 2:**
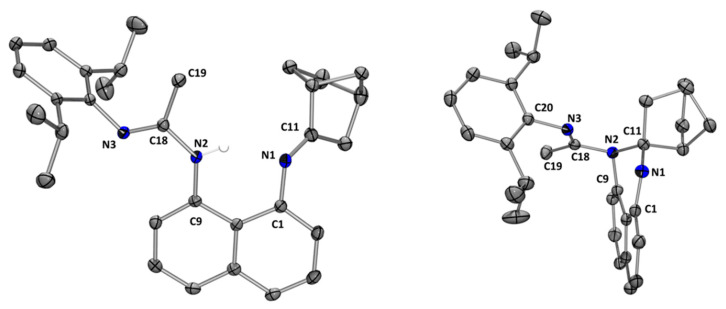
Molecular structures of amidine–imine and amidine–aminal isomers of ligand **LH**. Thermal ellipsoids are represented with a 30% probability. Hydrogen atoms (except H2 for amidine–imine) have been omitted for clarity.

Furthermore, both species were isolated by co-crystallization during the purification by column chromatography on silica (42% yield) and have been characterized in the solid state by X-ray diffraction (Figure 2 and Figures S19–S21). The presence of an intramolecular hydrogen bond between the N-H group of the amidine moiety and the nitrogen atom of the imine is confirmed in the solid-state structure of the amidine–imine isomer.

### 2.2. Synthesis and Characterization of New Aluminum(III) Complexes with an Asymmetric Amidine–Amine Ligand

**Scheme 2:**
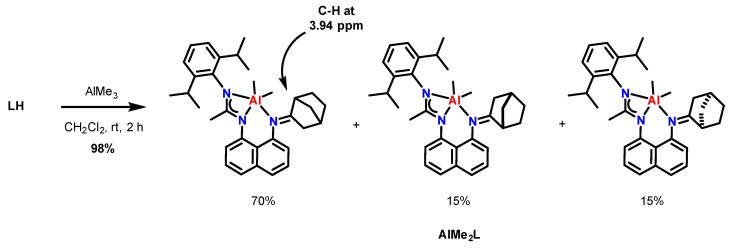
Synthesis of **AlMe_2_L** from **LH** and AlMe_3_.

The dimethylated aluminum complex (**AlMe_2_L**) was synthesized starting from the mixture of isomers (**LH**) and a solution of trimethylaluminum (2.0 M in heptane) in dichloromethane at room temperature (Scheme 2). The pure product could be extracted from the crude with dry pentane (98% yield). The crystalline product of **AlMe_2_L** was fully characterized by NMR, MS (Maldi-TOF), and X-ray diffraction.

**Figure 3:**
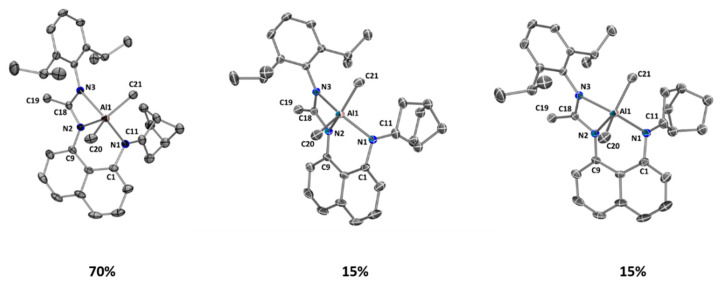
Molecular structures of stereomers of **AlMe_2_L**. Thermal ellipsoids are represented with a 30% probability. Hydrogen atoms and solvent molecules have been omitted for clarity.

The X-ray diffraction analysis revealed the formation of a pentacoordinate aluminum complex with the ligand in the amidine–imine form as a mixture of three stereomers in a 70/15/15 ratio in the crystal (Figure 3, Figures S22 and S23), which explains the rather complicated NMR spectra in solution. These stereomers arise from the *Z*/*E* configuration of the imine and the R*/S* configuration of the two stereocenters present in the bicycle (which was initially used as a racemic mixture). The crystalline structure revealed that the pentacoordinate aluminum atom exhibits a distorted square pyramidal geometry. The Al-C20 and Al-C21 bond lengths of 1.966 Å and 1.984 Å, respectively (see Supplementary Materials, Table S11) are close to the length of 1.964 Å described for a symmetrical system [14].

The ^1^H-NMR analysis of **AlMe_2_L** (see Supplementary Materials, Figure S8) shows that the peaks corresponding to the methyl groups bonded to the aluminum center, at −0.78 and −0.88 ppm, do not have the same integrations, illustrating the presence of stereomers in solution. Furthermore, focusing on the signal at 3.94 ppm corresponding to the C-H proton of the alpha carbon in the norbornane bicycle of the major stereomer, we can observe that it is displaced towards lower field, indicating a possible interaction with one of the methyl groups bonded to the aluminum center (see Scheme 2). The other protons corresponding to the bicycle group are shifted to higher field, between 2.7 and 1.2 ppm.

**Figure 4:**
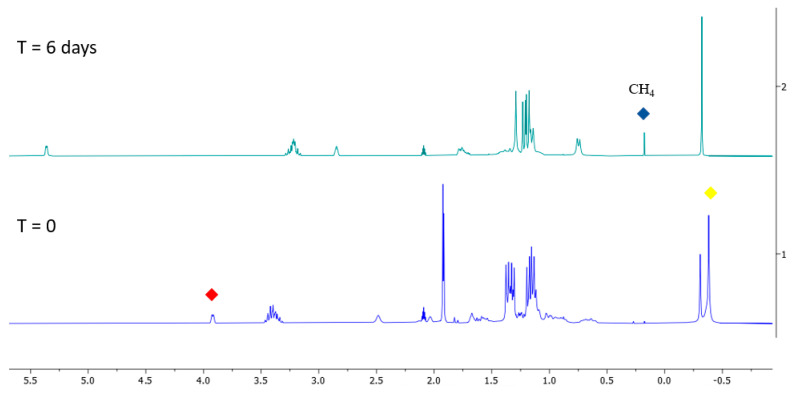
Comparison of ^1^H-NMR spectra of **AlMe_2_L** heated at 100 °C (Toluene-d_8_, 300 MHz): *T* = 0 and *T* = 6 days.

**Scheme 3:**
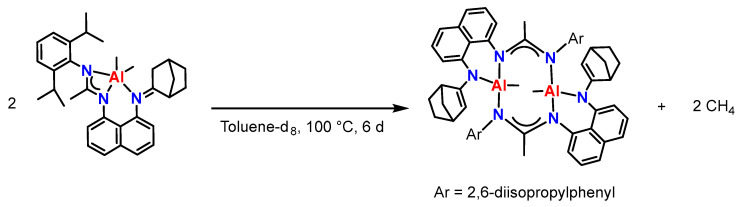
Al_2_Me_2_L_2_ obtention.

To determine the thermal stability and potentially corroborate a reactivity associated with this possible interaction, **AlMe_2_L** was dissolved in toluene-d_8_, heated to 100 °C (see Scheme 3), and monitored by ^1^H-NMR (Figure 4). After 24 h, in addition to the release of methane, two new peaks began to increase (see Supplementary Materials, Figure S10): one between the methyl peaks at −0.32 ppm and another at 5.35 ppm (enamine proton), while the proton at 3.94 ppm was decreasing. At the end of 6 days under these conditions, complete conversion was achieved (Figure 4, *T* = 6 days). The solvent was then removed under vacuum, and the resulting oil was mixed with dry dichloromethane and kept at −30 °C for three days until yellow crystals formed in quantitative yield.

**Figure 5:**
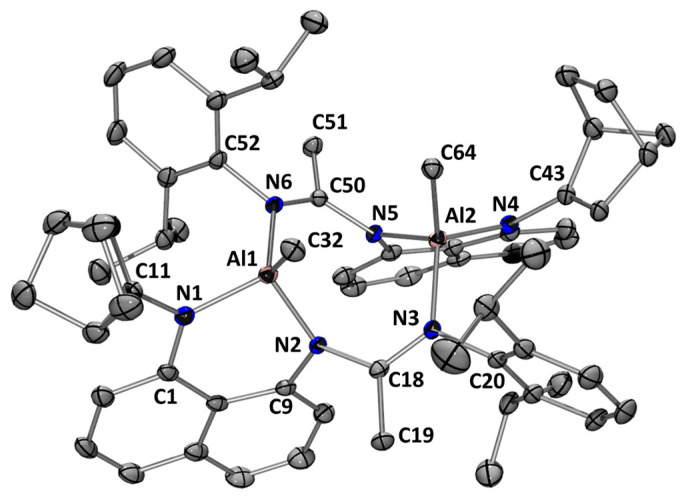
Molecular structure of **Al_2_Me_2_L_2_**. Thermal ellipsoids are represented with a 30% probability. Hydrogen atoms have been omitted for clarity.

The X-ray analysis of the crystals revealed a dimeric aluminum(III) complex (**Al_2_Me_2_L_2_**) (Figure 5 and Figure S24), where one of the methyl groups from the **AlMe_2_L** extracted a proton from the alpha carbon to the imine in the norcamphor moiety, resulting in an amidinate-enamine coordination of the ligand. The crystalline structure confirmed that the aluminum atoms are tetracoordinated with a distorted tetrahedral geometry. The Al−C bond distances for each Al center of 1.934 Å and 1.944 Å, respectively, are slightly shorter than in the starting pentacoordinated aluminum complex (see Supplementary Materials, Table S15).

**Scheme 4:**
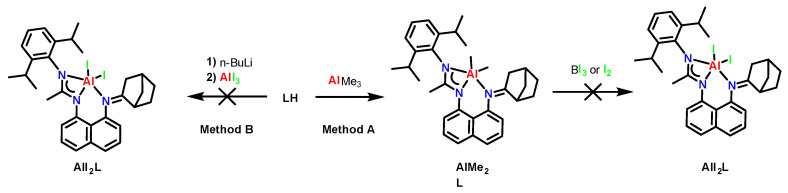
Pathways for **AlI_2_L** obtention attempts.

To synthesize the corresponding dihalogenated complex **AlI_2_L**, a potential catalyst for the preparation of cyclic carbonates from CO_2_ and epoxides, we tested two different methods (Scheme 4). The first one involves the exchange of methyl groups for the halogens of a halogenating agent [14] (BI_3_ and I_2_ were tested) in different conditions of solvent and temperature (Scheme 4, Method **A**).

**Figure 6:**
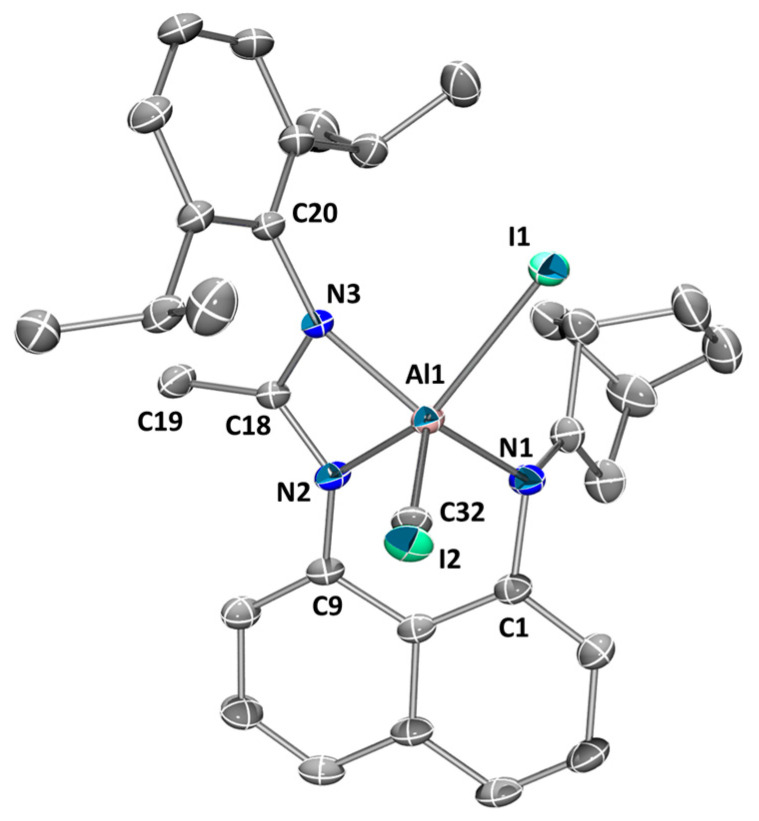
Molecular structure of **AlMeIL**/**AlI_2_L** mixture. Thermal ellipsoids are represented with a 30% probability. Hydrogen atoms have been omitted for clarity.

Still, in every case, instead of noticing the disappearance of the methyl groups bonded to the aluminum center, additional methyl signals continued to appear in the ^1^H-NMR. This surprising result, probably linked to a difference in reactivity of the different methyl groups present in the mixture of isomers, could be confirmed by the formation of crystals, as minority products, which could be analyzed by X-ray diffraction (see Supplementary Materials, Figures S27 and S28). The structure shows a mixture of products in a 97:3 ratio, where the major product corresponds to a methyl-iodide aluminum complex (**AlMeIL**), and the minor one to the desired **AlI_2_L** (Figure 6). For the major product **AlMeIL**, the aluminum center, substituted by one methyl group and one iodine atom, becomes stereogenic, thus leading to the formation of additional diastereomers, and inducing the formation of additional signals in ^1^H-NMR for the different methyl groups.

The second method (Scheme 4, Method **B**) consists of a metathesis reaction between the deprotonated ligand and one halogenated metal salt, AlI_3_ [19]. However, although the deprotonation reaction of the **LH** ligand can be carried out by different bases, such as n-butyl lithium, KHMDS, or KH (see Supplementary Materials Figure S13 for ^1^H NMR of **LK**), the reaction of the generated anion on AlI_3_ never led to the formation of the expected product **AlI_2_L**, and we have observed a re-protonation of the ligand. These phenomena are common in compounds derived from 1,8-diaminonaphthalene since they are “proton sponge” compounds. These classes of compounds are extremely basic, with pKa values over 15 in some aprotic solvents, and can rapidly absorb protons [20]. (Trifonov and co-workers obtained a similar re-protonation example in 2017, where they did a salt metathesis reaction between a 1,8-diaminonaphthalene-derived lithium salt and YCl_3_ [21]).

**Scheme 5:**
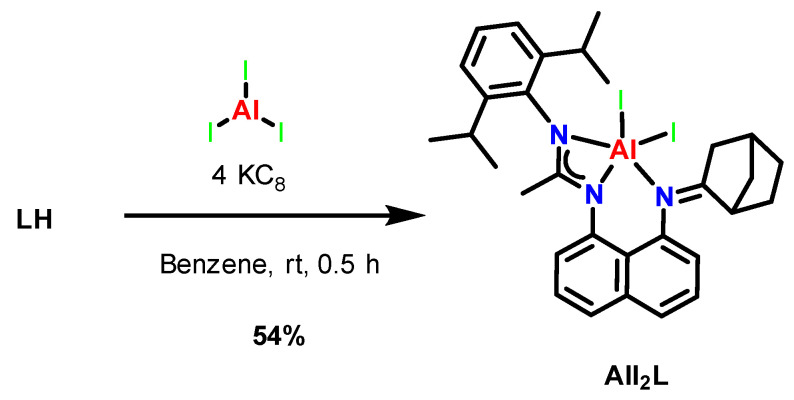
Unusual synthetic route for **AlI_2_L** obtention.

**Figure 7:**
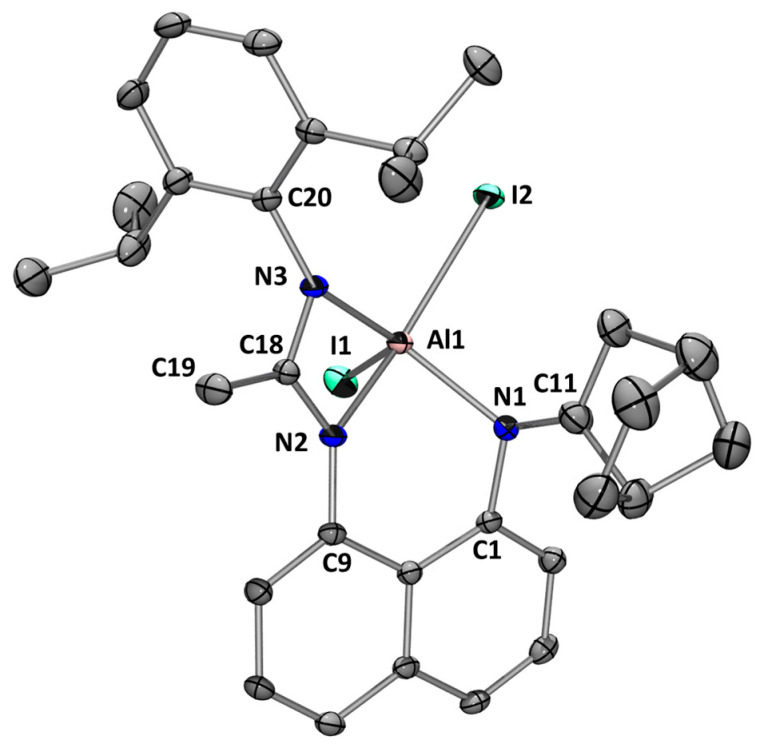
Molecular structure of **AlI_2_L**. Thermal ellipsoids are represented with a 30% probability. Hydrogen atoms and solvent molecules have been omitted for clarity.

Faced with this lack of results for the formation of **AlI_2_L** according to the two methods previously tested, the solution appeared to us through serendipity. Indeed, while we were interested in parallel in the synthesis of low-valent aluminum species [22,23], we have tested conditions described by Roesky and co-workers in 2017 for the formation of a stable neutral radical aluminum complex [24], by reacting **LH** with aluminum triiodide salt AlI_3_ and an excess of KC_8_ in benzene at room temperature (Scheme 5). After 30 min of reaction and subsequent filtration, yellow crystals began settling into the flask’s bottom. After isolation and characterization in solution (see Supplementary Materials Figures S14 and S15) and in solid state (Figure 7, Figures S25 and S26), we have confirmed the surprising formation of **AlI_2_L** complex in 54% yield. Interestingly, without the presence of KC_8_, the reaction was not reproducible. Moreover, ^1^H-NMR analysis of the black residual solid obtained after filtration has shown the concomitant formation of KH (see Supplementary Materials, Figure S16). The crystalline structure of **AlI_2_L** revealed that the pentacoordinate aluminum atom exhibits a distorted trigonal bipyramidal geometry. The Al-I1 and Al-I2 bond lengths are non-equivalent (2.561 Å and 2.635 Å, respectively, see Supplementary Materials Table S19), and longer than the length of 2.533 Å described for a symmetrical system [14].

**Scheme 6:**
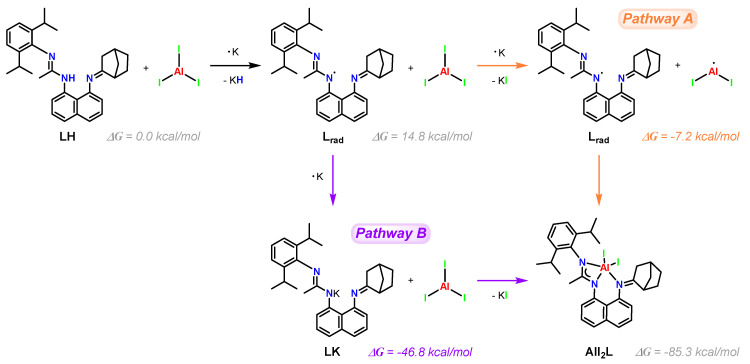
Proposed mechanism for synthesizing **AlI_2_L** in the presence of **LH**, AlI_3,_ and a potassium metal atom as a reductive agent to model KC_8_. Relative Gibbs free energies (Δ*G*) were calculated at the PCM(benzene)-(U)M06-2X/6-311++G(d,p),aug-cc-pVTZ-PP(I)//(U)M06-2X/6-31G(d,p),LANL2DZ(I) level of theory.

The formation of an aluminum(III) complex after performing the reaction with an excess of the reducing agent is a somewhat atypical result. To gain deeper insight into this result, DFT calculations were conducted at the PCM(benzene)-(U)M06-2X/6-311++G(d,p),aug-cc-pVTZ-PP(I)//(U)M06-2X/6-31G(d,p),LANL2DZ(I) level of theory (Scheme 6, see Supplementary Materials for further computational details). Given the structural complexity of KC_8_, where potassium atoms are intercalated between graphite layers and render it unsuitable for standard DFT treatments, we modeled it as a potassium metal atom with a single valence electron [25]. Furthermore, given the nature of the reaction, which involves radical processes without well-defined transition states, we focus on assessing the thermodynamic feasibility of the proposed mechanisms rather than explicitly characterizing reaction pathways. First, considering the overall reaction, we observe an energy difference of −85.3 kcal/mol between the reactant **LH** and the product **AlI_2_L**. The magnitude of this value indicates a strong thermodynamic driving force for product formation, which is consistent with the mild and rapid experimental conditions regarding temperature and reaction time.

The first step in the mechanism involves the deprotonation of **LH** by potassium through an endergonic hydrogen atom transfer (HAT) process (ΔG = +14.8 kcal/mol), leading to the formation of the radical species L_rad_ and potassium hydride. Subsequently, two possible pathways are considered. The first pathway, denoted as Pathway A (in orange), involves a reaction between a potassium atom and AlI_3_, yielding potassium iodide and the AlI_2_ radical species, a process that is relatively favorable with a relative Gibbs free energy (ΔG) of −7.2 kcal/mol compared to the reactants. The subsequent coupling of these two radical species leads to the formation of the desired product, **AlI_2_L**. In contrast, Pathway B (in purple) begins with a radical coupling step, forming the significantly favorable intermediate **LK** (ΔG = −46.8 kcal/mol), which could subsequently react with AlI_3_ to yield the final product.

Ultimately, from a purely thermodynamic standpoint, Pathway B appears to be the favored route, although Pathway A remains plausible. Indeed, considering factors that could influence the reaction kinetics, such as steric hindrance, it can be assumed that the final step of Pathway A would be more favorable than that of Pathway B, as it involves the approach of AlI_3_, a bulkier species.

### 2.3. Catalytic Evaluation of AlMe_2_L as a Two-Component System for the Synthesis of Cyclic Carbonates

The complex **AlMe_2_L** (1.5 mol%) was evaluated in a two-component catalytic system for the formation of cyclic carbonate from epoxides and CO_2_, with tetrabutylammonium iodide (TBAI) as co-catalyst (1.5 mol%).

Initially, the reactions were carried out at 80 °C under 1 bar of CO_2_ pressure for 24 h under solvent-free conditions to easily compare the activity of **AlMe_2_L** to our previously published studies [14]. The results are summarized in Table 1.

Styrene epoxide (**1a**, Table 1, entry 1) yielded a promising 90% conversion in cyclic carbonates. Relatively close conversions were obtained for alkyl substituted epoxides **1b** and **1c**, with 86% and 83%, respectively (Table 1, entries 3 and 5). For epoxides bearing a fluorinated alkyl chain **1d** or halogenated aryl moieties (**1e**, **1f**), conversion dropped significantly between 22% and 36% (Table 1, entries 7, 9, 11). Increasing the reaction temperature to 90 °C was beneficial in every case, especially for the epoxide bearing the fluorinated chain (**1d**) where the conversion was multiplied by 2.5 (Table 1, entries 7 and 8). For epoxides (**1a**–**d**), conversions ranging from 80% to 98% were obtained (Table 1, entries 2, 4, 6, 8).

**Scheme 7:**
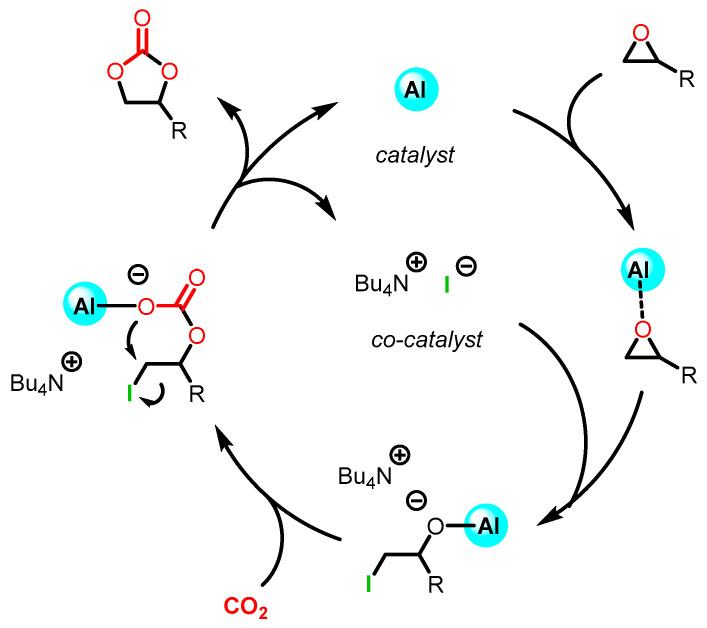
Proposed catalytic cycle for cyclic carbonates formation from epoxides and CO_2_ using aluminum complexes and TBAI.

A commonly investigated and widely accepted mechanism for this two-component catalytic system involves several stages (Scheme 7) [26,27,28]. Initially, one of the free electron pairs on the oxygen atom within the epoxide is coordinated to the metal center, leading to a redistribution of electron density within the epoxide group and an increase in the electrophilicity of the carbon atoms within the cycle. Subsequently, an iodide ion from a cocatalyst molecule (TBAI in this case) present in the reaction environment acts as a nucleophile, targeting the carbon atom in α or β position depending on electronic or steric parameters. Following the epoxide opening, a CO_2_ molecule inserts into the Al−O bond, inducing a charge transfer that ultimately yields a formal negative charge on one of the oxygen atoms. This oxygen atom then serves as a nucleophile, attacking the carbon atom bonded to the iodide, causing the dissociation of iodide from the molecule and forming a five-membered cyclic carbonate. This cyclic carbonate is promptly liberated from the catalyst center.

### 2.4. Catalytic Evaluation of AlI_2_L as a Single-Component Catalyst for the Synthesis of Cyclic Carbonates

For evaluation of **AlI_2_L** (1.5 mol%), no co-catalyst was used because of the presence of the iodide atoms at the aluminum center (the dissociation of the iodine anion of the complex allowing its nucleophilic attack to the carbon atom of the epoxide in the catalytic cycle). Using the above reaction conditions, the reaction proved to be sluggish for every epoxide (See Table 2). The best result being a 21% conversion, obtained with styrene epoxide (**1a**, Table 2, entry 1). Increasing the carbon dioxide pressure from 1 to 5 bar, keeping the other conditions unchanged, led to a considerable improvement. The conversions were improved by a factor ranging from 4 to 8 (Table 2).

The obtained catalytic results may suggest a change in the CO_2_ reaction order, from 0 to a positive value, where the CO_2_ insertion in the alkoxide intermediate is probably the rate-limiting step [29,30]. This behavior contrasts with other catalytic systems in which the epoxide ring-opening step is rate-limiting, and the influence of CO_2_ pressure may be negligible. In the study of Tian et al., the single-component architecture of their complex enables highly efficient epoxide activation and ring-opening, effectively shifting the kinetic bottleneck to the CO_2_ insertion step. Consequently, increasing the CO_2_ pressure enhances its solubility in the reaction media and lowers the activation barrier for carbonate intermediate formation, thereby accelerating the overall catalytic cycle [29].

These new catalytic systems (**AlMe_2_L**/TBAI) and **AlI_2_L** (without addition of any co-catalyst), using an asymmetric amidine–imine **L** ligand, show interesting reactivities, comparable to those previously described in the literature for symmetrical systems [14,27,31,32]. The recyclability of the catalyst, due to the type of catalysis (homogeneous catalysis) and the experimental conditions (without solvent, mol%...), is not possible.

### 2.5. Conclusions

This study successfully synthesized and characterized a novel amidine–imine ligand derived from 1,8-diaminonaphthalene and explored its coordination chemistry with aluminum(III). The ligand demonstrated versatile coordination behavior, forming complexes with different structural arrangements, including an amidine–enamine ligand resulting from a unique transformation.

The synthesis of a dihalogenated aluminum complex (**AlI_2_L**) revealed an unconventional formation pathway involving a reducing agent, highlighting the complex reactivity of this system. The complexes **AlMe_2_L** and **AlI_2_L** were evaluated as catalysts for converting CO_2_ and epoxides into cyclic carbonates in two-component (with TBAI) and single-component systems, respectively, exhibiting moderate catalytic activity, with performance influenced by CO_2_ pressure. The lower yields obtained by using a single-component system can be explained by a steric hindrance around the aluminum center.

## 3. Materials and Methods

### 3.1. General Comments

All manipulations were performed under an inert argon or nitrogen atmosphere using standard Schlenk−line and glovebox techniques. Dry oxygen−free solvents were employed. Reagents were obtained from commercial suppliers unless otherwise stated. *N*-imidoylphenyl chloride was synthesized following a reported procedure [33]. 1D and 2D NMR spectra were recorded with the following spectrometers for ^1^H and ^13^C: Bruker Avance II 300 MHz, Avance III HD 400 MHz, and Avance I and II 500 MHz spectrometers (Brucker, Karlsruhe, Germany). The chemical shift has been counted positively verse the low field and expressed in parts per million (ppm). The mass spectrometric analysis was performed using electrospray ionization recorded on a Waters Xevo G2 Q-TOF mass spectrometer (Waters, Los Angeles, CA, USA) and a Maldi micro−MX micro–Mass in a pyrene matrix (ratio product/matrix:1/100). Melting points were measured with a capillary Electrothermal SMP40 apparatus (Stuart, Staffordshire, UK) and samples were prepared in the glovebox before the analysis. Single-crystal X-ray data were collected at low temperature (193(2)K) on a Bruker APEX II Quazar diffractometer (Billerica, MA, USA) equipped with a 30W air-cooled microfocus source [**LH(amidine–imine)** and **Al_2_Me_2_L_2_**] or on a Bruker D8 VENTURE diffractometer (Billerica, MA, USA) equipped with a PHOTON III detector [**LH(amidine–aminal)**, **AlMe_2_L**, **AlIMeL** and **AlI_2_L**], using MoK_α_ radiation (λ = 0.71037 Å) or CuK_α_ radiation (λ = 1.54178 Å). Phi and Omega scans were performed for data collection, and an empirical absorption correction was applied [34]. The structure was solved by the intrinsic phasing method (ShelXT) [35] and refined by the full-matrix least-squares method on F^2^ [36]. All non-hydrogen atoms were refined with anisotropic displacement parameters, while hydrogen atoms were refined isotropically at calculated positions using a riding model, except the N-bound hydrogen atoms located in difference Fourier maps and refined freely for **LH(amidine–imine)** or with *U*_iso_(H) = 1.2*U*_eq_(N) for **LH(amidine–aminal)**. All calculations were carried out using density functional theory (DFT) [37,38] as implemented in the Gaussian 16 software [39] package employing the (U)M06-2X [40] hybrid functional. Geometry optimizations and analytical frequency calculations were performed using the 6-31G(d,p) triple-ζ basis set [41,42] for C, N, H, and Al atoms, while the LANL2DZ [43,44] effective core potential was used for iodine. Frequency analyses confirmed that all optimized structures correspond to true minima, as all vibrational frequencies were found to be real (i.e., positive). Subsequently, single-point energy calculations were carried out on the optimized geometries using an augmented basis set: 6-311++G(d,p) [45,46,47] for C, N, H, and Al atoms, and aug-cc-pVTZ-PP [48] for iodine. Thermodynamic corrections corresponding to standard conditions (298 K, 1 atm) were taken into account to report reaction pathways in terms of standard Gibbs free energies. Solvent effects were included in all calculations using the polarizable continuum model [49,50] (PCM), with benzene as the solvent, in line with experimental conditions. Due to the structural complexity of KC_8_, where potassium atoms are intercalated between graphite layers, making it unsuitable for standard DFT treatments, it was modeled as a potassium atom bearing a single valence electron [25]. In addition, since the proposed mechanisms involve radical species, all calculations were performed under open-shell (unrestricted) conditions. It is worth noting that the geometry of the final compound **AlI_2_L** was constructed based on the available single-crystal X-ray diffraction data.

### 3.2. Synthesis

***Synthesis of aminal precursor*.** This compound was synthesized by adapting the conditions from a patent [51]. 1,8-diaminonaphthalene (1,8-DAN) (15 g, 95 mmol), norcamphor (11.49 g, 104 mmol), and a catalytic amount (3 mg) of p-toluenesulfonic acid were solubilized in toluene (60 mL). The solution was kept under reflux for 4 h in a Dean-Stark system. After this time, the solvent is removed under reduced pressure, and a sticky purple-brown product is obtained. The product was washed with cyclohexane (3 × 20 mL), and a purple-brown powder was obtained (22.68 g, 91 mmol, 96%). **^1^H NMR** (400 MHz, Dimethylsulfoxide-d_6_, 25 °C): δ/ppm 7.09 (m, 2H), 6.86 (d, *J* = 8.2 Hz, 2H), 6.52 (s, 2H), 6.45–6.39 (m, 2H), 2.24–2.17 (m, 1H), 2.08 (d, *J* = 4.0 Hz, 1H), 1.83 (m, 1H), 1.76 (m, 1H), 1.61 (m, 1H), 1.49 (m, 1H), 1.43–1.33 (m, 2H), 1.22–1.11 (m, 2H). **^13^C{^1^H} NMR** (100 MHz, Chloroform-d_1_, 25 °C): δ/ppm 142.7 (C_(C10H6)_), 142.4 (C_(C10H6)_), 134.4 (C_(C10H6)_), 127.0 (CH_(C10H6)_), 126.9 (CH_(C10H6)_), 114.2 (CH_(C10H6)_), 114.1 (CH_(C10H6)_), 113.0 (C_(C10H6)_), 103.8 (CH_(C10H6)_), 103.3 (CH_(C10H6)_), 72.9 (N-C-N), 45.8 (CH_2(C7H10)_), 44.4 (CH_(C7H10)_), 36.7 (CH_2(C7H10)_), 35.3 (CH_(C7H10)_), 27.8 (CH_2(C7H10)_), 21.6 (CH_2(C7H10)_). **HRMS** (DCI-CH_4_) *m*/*z*: 251.1557 ([M + H]^+^) calcd for C_17_H_19_N_2_^+^ 251.1543

***Synthesis of LH*.** The procedure followed was reported by our group in previous work [14]. In a three-neck flask, 8 g (31.36 mmol) of the aminal compound was added, followed by 60 mL of dry toluene and 3.56 g (4.90 mL, 35.15 mmol) of dry triethylamine. One free neck was connected to the Schlenk line to administer argon, while the other entry was covered with a septum. 8.36 g (35.15 mmol) of *N*-(2,6-diisopropylphenyl)acetimidoyl chloride was slowly added to the solution. The reaction was left in reflux, stirring, and under an argon atmosphere for 16 h. After the reaction time, a filtration was performed to remove the ammonium salts; then, the toluene was removed in a rotary evaporator. The resulting oil was solubilized in 50 mL of dichloromethane and was washed with a 20% ammonia solution (3 × 20 mL). The organic layer was dried with anhydrous Na_2_SO_4_, then filtered, and the DCM was removed on a rotary evaporator. The product was purified by a chromatographic column through basic silica using a 20% ethyl acetate and 80% pentane mixture as the eluent. The orange solution was left at room temperature overnight, obtaining 5.87 g of orange crystals (13.06 mmol, 42% yield). **^1^H NMR** (400 MHz, Chloroform-d_1_, 25 °C): δ/ppm 10,74 (s, 1H, major isomer N-H), 10.64 (s, 1H, minor isomer N-H) 9.30–9.22 (m, 1H, C_10_H_6_) 7.65–7.57 (2dd, *J* = 6.2, 1.25 Hz minor aminal, *J* = 6.2, 1.3 Hz major imine, overlapping signals, C_10_H_6_), 7.48–7.28 (m, 3H, C_10_H_6_), 7.37–7.30 (m, 1H, C_10_H_6_), 7.20 (m, 2H, C_6_H_3_), 7.11 (m, 2H, C_6_H_3_), 6.80–6.67 (2dd, *J* = 7.3, 1.3 Hz major, *J* = 7.4, 1.2 Hz minor, 1H, C_10_H_6_), 3.1–2.97 (m, 2H, CH-*^i^*Pr), 2,64 (m, 1H, C_7_H_10_), 2.39 (m, 1H, C_7_H_10_), 2.30 (m, 1H, C_7_H_10_), 2.04 (dd, *J* = 17.9, 3.5 Hz, 1H, C_7_H_10_), 1.93 (2s, one for each isomer, 3H, NNC-CH_3_), 1.75–1.51 (m, 4H, C_7_H_10_), 1.25–1.10 (m, 12H, *^i^*Pr-CH_3_), 0,60 (m, 2H, C_7_H_10_). **^13^C{^1^H} NMR** (400 MHz, Chloroform-d_1_, 25 °C): δ/ppm 185.1 (C꞊N), 168.1 (N꞊C-N), 146.0 (C_(C10H6)_), 145.9 (C_(C6H3)_), 138.8 (CH_(C10H6)_), 138.0 (C_(C6H3)_), 126.5 (C_(C10H6)_), 126.4 (CH_(C10H6)_), 126.0 (C_(C10H6)_), 125.9 (C_(C6H3)_), 125.3 (CH_(C6H3)_), 125.2 (CH_(C10H6)_), 123.5 (CH_(C10H6)_), 123.5 (CH_(C6H3)_), 117.0 (CH_(C10H6)_), 116.6 (CH_(C6H3)_), 116.5 (CH_(C10H6)_), 116.4 (CH_(C10H6)_), 48.7 (C_7_H_10_), 43.4 (C_7_H_10_), 41.8 (C_7_H_10_), 38.6 (C_7_H_10_), 38.1 (C_7_H_10_), 36.0 (CH-*^i^*Pr), 35.1 (CH-*^i^*Pr), 27.8 (*^i^*Pr-CH_3_), 27.5 (*^i^*Pr-CH_3_), 26.6 (*^i^*Pr-CH_3_), 26.4 (*^i^*Pr-CH_3_), 25.8 (C_7_H_10_), 25.7 (NNC-CH_3_). **HRMS** (DCI-CH_4_) *m*/*z*: 452.3078 ([M + H]+) calcd for C_31_H_38_N_3_ + 452.3066.

***Synthesis of complex AlMe_2_L*.** Trimethylaluminum 2M (298.8 mg, 4.14 mmol) was added to a solution of **LH** (1.87 g, 4.14 mmol) in 5 mL of dry dichloromethane. After 1 h of stirring at room temperature, the solvent was removed under reduced pressure. The resulting product was extracted with pentane (3 × 3 mL), keeping it at −30 °C until a yellow-orange crystalline solid was obtained (2.10 g, 4.13 mmol, 98% yield). **^1^H NMR** (400 MHz, Chloroform-d_1_, 25 °C): δ/ppm 7.66 (ddd, *J* = 9.6, 8.2, 1.2 Hz, 1H, C_10_H_6_), 7.42–7.27 (m, 3H, C_6_H_3_), 7.18–7.12 (m, 4H, C_10_H_6_), 6.85 (ddd, *J* = 9.8, 7.3, 1.2 Hz, 1H, C_10_H_6_), 3.94 (d, *J* = 4.7 Hz, 1H, C_7_H_10_), 3.27–3.09 (m, 2H, CH-*^i^*Pr), 2.72–2.51 (m, 1H, C_7_H_10_), 2.36 (s, 1H, C_7_H_10_), 2.12 (d, *J* = 3.6 Hz, 3H, NNC-CH_3_), 2.01–1.82 (m, 1H, C_7_H_10_), 1.75–1.28 (m, 6H, C_7_H_10_), 1.25–1.07 (m, 12H, *^i^*Pr-CH_3_), −0.78 (s, 2H, Al-CH_3_), −0.88 (s, 3H, Al-CH_3_). **^13^C{^1^H} NMR** (400 MHz, Chloroform-d_1_, 25 °C): δ/ppm 192,6 (C꞊N), 168.9 (N꞊C‒N), 146.0 (C_(C10H6)_), 145.3 (C_(C10H6)_), 143.5 (C_(C6H3)_), 143.1 (C_(C6H3)_), 140.6 (C_(C6H3)_), 136.2 (C_(C10H6)_), 127.3 (CH_(C10H6)_), 127.1 (d, *J* = 6.2 Hz, CH_(C6H3)_) 126.7 (d, *J* = 5.7 Hz, CH_(C10H6)_), 125.5 (CH_(C6H3)_), 125.0 (CH_(C10H6)_), 122.5 (C_(C10H6)_), 119.4 (CH_(C10H6)_), 119.3 (CH_(C10H6)_), 119.2 (CH_(C6H3)_), 38.2 (C_7_H_10_), 35.1 (C_7_H_10_), 28.4 (CH-*^i^*Pr), 28.3 (CH-*^i^*Pr), 27.1 (C_7_H_10_), 26.9 (C_7_H_10_), 26.6 (C_7_H_10_), 24.9 (d, *J* = 5.4 Hz, *^i^*Pr-CH_3_), 24.8 (d, *J* = 6.6 Hz, *^i^*Pr-CH_3_), 24.5 (C_7_H_10_), 24.4 (d, *J* = 5.8 Hz, *^i^*Pr-CH_3_), 24.3 (d, *J* = 6.2 Hz, *^i^*Pr-CH_3_), 18.4 (NNC-CH_3_), −3.5 (Al-CH_3_), −4.9 (Al-CH_3_ **MS** (Maldi-TOF) *m*/*z*: 507.3 ([M]^+^), 492.5 ([M-CH_3_]^+^), 450.4 ([M-Al(CH_3_)_2_]^+^).

***Synthesis of complex Al_2_Me_2_L_2_*.** 30 mg of **AlMe_2_L** (0.06 mmol) was solubilized in 0.3 mL of toluene-d_8_ and was heated at 100 °C for 6 days in an NMR Young tube. After this time, the toluene was removed, and a yellow oil was obtained; this one was solubilized in dry dichloromethane and kept at −30 °C for 3 days. 29 mg of yellow crystals were obtained (0.03 mmol, 99% yield). **^1^H NMR** (600 MHz, Toluene-d_8_, 25 °C): δ/ppm 7.54–7.47 (m, 2H, C_10_H_6_), 7.30 (t, J = 7.8 Hz, 2H, C_10_H_6_), 7.20 (t, *J* = 0.8 Hz, 1H, C_6_H_3_), 7.19 (s, 1H, C_6_H_3_), 7.18 (dd, *J* = 1.2, 0.5 Hz, 1H, C_6_H_3_), 7.17 (d, *J* = 1.1 Hz, 1H, C_6_H_3_), 7.16 (s, 1H, C_10_H_6_), 7.08 (d, *J* = 1.0 Hz, 1H, C_6_H_3_), 7.07 (d, *J* = 0.9 Hz, 2H, C_10_H_6_), 7.06 (s, 1H, C_6_H_3_), 7.04 (d, *J* = 1.7 Hz, 1H, C_10_H_6_), 7.02 (s, 1H, C_10_H_6_), 7.01 (d, *J* = 1.2 Hz, 1H, C_10_H_6_), 6.99 (d, *J* = 1.7 Hz, 4H, C_10_H_6_), 6.99–6.96 (m, 2H, C_10_H_6_), 5.36 (d, *J* = 3.0 Hz, 2H, -CH=C-N), 3.39–3.05 (m, 8H, CH-*^i^*Pr and C_7_H_9_), 2.84 (dq, *J* = 3.3, 1.6 Hz, 2H, C_7_H_9_), 1.86–1.62 (m, 4H, C_7_H_9_), 1.47–1.32 (m, 2H, C_7_H_9_), 1.29 (s, 8H, NNC-CH_3_ and C_7_H_9_), 1.22 (d, *J* = 6.8 Hz, 6H, *^i^*Pr-CH_3_), 1.20 (d, *J* = 6.8 Hz, 6H, *^i^*Pr-CH_3_), 1.17–1.13 (m, 8H, *^i^*Pr-CH_3_ and C_7_H_9_), 1.10 (d, *J* = 10.8 Hz, 2H, C_7_H_9_), 0.74 (d, *J* = 6.8 Hz, 6H, *^i^*Pr-CH_3_), −0.34 (s, 6H, Al-CH_3_). **^13^C{^1^H} NMR** (151 MHz, Toluene-d_8_, 25 °C): δ/ppm 182.0 (N=C-N), 154.5 (C_7_H_9_), 148.2 (C_(C6H3)_), 144.3 (C_(C6H3)_), 143.8 (C_(C6H3)_), 141.0 (C_(C10H6)_), 137.5 (C_(C10H6)_), 128.8 (C_(C10H6)_), 128.7 (C_(C10H6)_), 126.5 (C_(C10H6)_), 126.2 (C_(C10H6)_), 124.9 (C_(C6H3)_), 124.3 (C_(C6H3)_), 124.2 (C_(C10H6)_), 124.1 (C_(C10H6)_), 120.3 (C_(C6H3)_), 119.6 (C_(C10H6)_), 118.4 (C_(C10H6)_), 117.8 (C_7_H_9_), 46.8 (C_7_H_9_), 43.1 (C_7_H_9_), 42.5 (C_7_H_9_), 29.6 (C_7_H_9_), 28.9 (CH-*^i^*Pr), 28.4 (CH-*^i^*Pr), 25.5 (C_7_H_9_), 24.6 (*^i^*Pr-CH_3_), 24.3 (*^i^*Pr-CH_3_), 24.1 (*^i^*Pr-CH_3_), 23.6 (*^i^*Pr-CH_3_), 13.9 (NNC-CH_3_), −4.4 (Al-CH_3_).

***Synthesis of complex AlI_2_L*.** 100 mg (0.22 mmol) of **LH**, 90.27 mg (0.22 mmol) of AlI_3_, and 119.73 mg (0.89 mmol) of KC_8_ were mixed in 2 mL of dry benzene for 30 min at room temperature. After this time, the solution was filtered, and the orange solution was kept at room temperature overnight. Orange crystals were observed, washed with pentane (3 × 1 mL), and dried under vacuum. 85 mg (0.12 mmol, 54% yield) was obtained. **^1^H NMR** (500 MHz, Dichloromethane-d_2_, 25 °C): δ/ppm 7.97–7.71 (m, 1H, C_10_H_6_), 7.58–7.41 (m, 3H, C_10_H_6_), 7.34–7.29 (m, 1H, C_6_H_3_), 7.24 (m, 2H, C_6_H_3_), 7.20–7.10 (m, 2H C_10_H_6_), 4.71 (d, *J* = 4.7 Hz, 1H, C_7_H_10_), 3.60–3.37 (m, 1H, CH-*^i^*Pr), 3.25–3.02 (m, 1H, CH-*^i^*Pr), 2.43 (d, *J* = 5.1 Hz, 1H, C_7_H_10_), 2.27 (d, *J* = 1.7 Hz, 1H, C_7_H_10_), 2.26 (d, *J* = 0.9 Hz, 1H, C_7_H_10_), 2.24 (s, 3H, NNC-CH_3_), 1.34–1.28 (m, 3H, *^i^*Pr-CH_3_), 1.27–1.22 (m, 3H, *^i^*Pr-CH_3_), 1.20 (d, *J* = 6.9 Hz, 4H, C_7_H_10_, *^i^*Pr-CH_3_), 1.14 (d, *J* = 6.9 Hz, 4H, C_7_H_10_, *^i^*Pr-CH_3_). **^13^C{^1^H} NMR** (126 MHz, Dichloromethane-d_2_, 25 °C): δ 199.5 (C=N), 170.7 (N=C-N), 146.6 (C_(C6H3)_), 146,4 (C_(C6H3)_), 145.2 (C_(C6H3)_), 142.6 (C_(C10H6)_), 142.5 (C_(C10H6)_), 136.4 (C_(C10H6)_), 135.8 (C_(C10H6)_), 128.8 (CH_(C10H6)_), 128.7 (CH_(C10H6)_), 127.4 (CH_(C6H3)_), 127.3 (CH_(C10H6)_), 127.3 (CH_(C6H3)_), 125.9 (CH_(C10H6)_), 123.2 (CH_(C6H3)_), 123.1 (CH_(C10H6)_), 114.5 (CH_(C10H6)_), 48.6 (C_7_H_10_), 42.0 (C_7_H_10_), 38.2 (C_7_H_10_), 35.2 (C_7_H_10_), 28.9 (CH-*^i^*Pr), 28.5 (CH-*^i^*Pr), 27.2 (C_7_H_10_), 25.7 (C_7_H_10_), 25.4 (*^i^*Pr-CH_3_), 24.7 (*^i^*Pr-CH_3_), 24.3 (*^i^*Pr-CH_3_), 24.0 (*^i^*Pr-CH_3_), 16.6 (NNC-CH_3_). **MS** (Maldi-TOF) *m*/*z*: 730.0 ([M]^+^), 604.1( [M-I]^+^).

#### General Catalysis

The epoxide (0.2 mL) and the catalyst **AI_2_L** (1.5 mol%) were placed in glass vials with a magnetic stirring bar in an autoclave reactor. The reaction mixture was stirred at 90 °C with 1 bar and 5 bar of CO_2_ for 24 h. The conversion of epoxide to cyclic carbonate was then determined by analyzing a sample using ^1^H NMR spectroscopy.

### 3.3. X-Ray Data

CCDC-2477487 (LH amidine–imine), CCDC-2477488 (LH amidine–aminal), CCDC-24774489 (Al_2_Me_2_L_2_) CCDC-2477490 (AlMe_2_L), CCDC-24774491 (AlIMeIAl) and CCDC-2477492 (AlI_2_L) contain the supplementary crystallographic data for this paper. These data can be obtained free of charge from The Cambridge Crystallographic Data Centre via www.ccdc.cam.ac.uk/data_request/cif, accessed on 31 July 2025.

## Figures and Tables

**Table 1:**
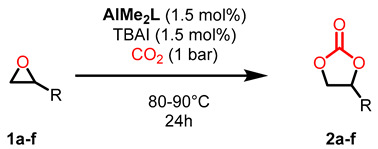
Formation of cyclic carbonates **2a**–**f** employing **AlMe_2_L** as catalyst ^a^.

Entry	Epoxides	Temperature [°C]	Yield [%] ^b^	TOF ^c^ [h^–1^]
1	**1a** (R = Ph)	80	90	2.50
2		90	98	2.73
3	**1b** (R = CH_2_Cl)	80	86	2.39
4		90	99	2.75
5	**1c** (R = n-bu)	80	83	2.31
6		90	87	2.42
7	**1d** (R = CH_2_OCH_2_(CF_2_)_3_CHF_2_)	80	32	0.89
8		90	80	2.23
9	**1e** (R = 4-ClPh)	80	22	0.61
10		90	37	1.00
11	**1f** (R = 4-BrPh)	80	36	1.00
12		90	58	1.60

^a^ Reactions were carried out at 1 bar of CO_2_ for 24 h, using 1.5 mol% of **AlMe_2_L** and 1.5 mol% TBAI under solvent-free conditions. ^b^ All yields were determined by ^1^H-NMR spectroscopy of the crude reaction mixture. ^c^ TOF: turnover frequency calculated as number of mol of product divided by number of mol of catalyst used per hour.

**Table 2:**
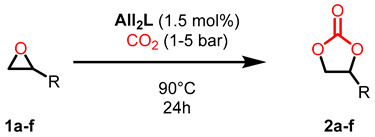
Formation of cyclic carbonates **2a**–**f** employing **AlI_2_L** as catalyst ^a^.

Entry	Epoxides	CO_2_ Pressure [bar]	Yield [%] ^b^	TOF ^c^ [h^–1^]
1	**1a** (R = Ph)	1	21	-
2		5	80	2.22
3	**1b** (R = CH_2_Cl)	1	<5	-
4		5	33	0.92
5	**1c** (R = n-bu)	1	17	-
6		5	78	2.16
7	**1d** (R = CH_2_OCH_2_(CF_2_)_3_CHF_2_)	1	<5	-
8		5	42	1.17
9	**1e** (R = 4-ClPh)	1	<5	-
10		5	28	0.78
11	**1f** (R = 4-BrPh)	1	11	-
12		5	71	1.94

^a^ Reactions were carried out at 90 °C for 24 h using 1.5 mol% of **AlI_2_L** under solvent-free conditions. ^b^ All yields were determined by ^1^H-NMR spectroscopy of the crude reaction mixture (See Supplementary Materials, Figure S17). ^c^ TOF: turnover frequency calculated as number of mol of product divided by number of mol of catalyst used per hour.

## Data Availability

Data are available on request from the authors.

## Supplementary Materials

The following are available online at https://www.mdpi.com/article/10.3390/molecules30193842/s1. NMR spectra, crystal structure refinements and computational investigations (PDF).
