# Supplementary material for: New Aluminum Complexes with an Asymmetric Amidine–Imine Ligand: Synthesis, Characterization, and Application in Catalysis
Source: Molecules. 2025 Sep 23;30(19):3842. doi: 10.3390/molecules30193842 (PMC12525682; doi:10.3390/molecules30193842)
Supplement: Supplementary file 1 [file molecules-30-03842-s001.zip › Supporting information-final revised 2nd revision.pdf]

Supporting information

New Aluminum Complexes with Amidine-Imine Ligands: Synthesis, Characterization, and Application in Catalysis

Fernando Gómez Zamorano,[a,b] María José Rojas,[b] Sonia Mallet−Ladeira,[c] Alan R. Cabrera,[b] Jordan Garo,[d] Jean-Marc Sotiropoulos,[d] Eddy Maerten,[a] David Madec, [a*] and René S. Rojas,[b*]

[a] Laboratoire Hétérochimie Fondamentale et Appliquée, Université de Toulouse, Centre National de la Recherche Scientifique (CNRS), UMR 5069, 118 Route de Narbonne, 31062 Toulouse Cedex 09, France.

[b] Departamento de Química Inorgánica, Facultad de Química y de Farmacia, Pontificia Universidad Católica de Chile, Vicuña Mackenna 4860, 7820436, Macul, Santiago, Chile.

[c] Institut de Chimie de Toulouse (UAR 2599), 118 Route de Narbonne, 31062 Toulouse Cedex 09, France.

[d] Universite de Pau et des Pays de l'Adour, CNRS, IPREM, Technopôle Hélioparc, 2 Avenue du Président Angot, 64053 Pau Cedex 09, France

TABLE OF CONTENTS

General information. ... 3

Figure S1. ^{1}H NMR spectrum of aminal precursor (DMSO-d_6, 400 MHz) ... 6

Figure S2. ^{13}C{^{1}H} NMR spectrum of aminal precursor (Chloroform-d_1, 125 MHz) ... 7

Figure S3. Aminal - amino-imine equilibrium. **A** = ^{1}H-NMR at 20°C, **B** = ^{1}H-NMR at 130°C ... 8

Figure S4. E/Z amidine-imine relation ... 9

Figure S5. ^{1}H NMR spectrum of **LH** (Chloroform-d_1, 400 MHz) ... 10

Figure S6. ^{13}C{^{1}H} NMR spectrum of **LH** (Chloroform-d_1, 101 MHz) ... 11

Figure S7. ^{1}H NMR spectrum of **LH** at -50 °C (Chloroform-d_1, 101 MHz) ... 12

Figure S8. ^{1}H NMR spectrum of **$AlMe_2L$** (Chloroform-d_1, 400 MHz) ... 13

Figure S9. ^{13}C{^{1}H} NMR spectrum of **$AlMe_2L$** (Chloroform-d_1, 101 MHz) ... 14

Figure S10. Comparison of ^{1}H-NMR spectrum of **$AlMe_2L$** heated at 100 °C for 6 d (Toluene-d_8, 300 MHz). 1- t = 0 d. 2- t = 1 d. 3- t = 2 d. 4- t = 4 d. 5- t = 5 d. 6- t = 6 d ... 15

Figure S11. ^{1}H NMR spectrum of **$Al_2Me_2L_2$** (Toluene-d_8, 600 MHz) ... 16

Figure S12. ^{13}C{^{1}H} NMR spectrum of **$Al_2Me_2L_2$** (Toluene-d_8, 151 MHz) ... 17

Figure S13. ^{1}H NMR spectrum of **LK** (THF-d_8, 300 MHz) ... 18

Figure S14. ^{1}H NMR spectrum of **AlI_2L** (Dichloromethane-d_2 , 400 MHz) ... 19

Figure S15. ^{13}C{^{1}H} NMR spectrum of **AlI_2L** (Dichloromethane-d_2, 126 MHz) ... 20

Figure S16. ^{1}H-NMR comparison between residual solid in the synthesis of **AlI_2L** and KH in THF-d_8. ... 21

Figure S17. **A**) ^{1}H-NMR pure styrene oxide in chloroform-d_1. **B**) ^{1}H-NMR in chloroform-d_1 of styrene carbonate and styrene oxide mixture after 24 h at 90 °C, 5 bar of CO_2, and 1.5 mol% of **AlI_2L**. ... 22

Figure S18. Mass Analysis ... 23

Crystal data and structure refinement of ligand LH (amidine-imine) ... 26

Crystal data and structure refinement of ligand LH (amidine-aminal) ... 35

Crystal data and structure refinement of complex $AlMe_2L$ ... 43

Crystal data and structure refinement of complex $Al_2Me_2L_2$ ... 58

Crystal data and structure refinement of complex AlI_2L ... 73

Crystal data and structure refinement of mixture complex AlI_2L/AlMeIL ... 87

Computational details ... 98

Cartesian coordinates of optimized structures ... 98

General information.

All manipulations were performed under an inert argon atmosphere using standard Schlenk-line and glovebox techniques. Dry, oxygen-free solvents were employed. Reagents were obtained from commercial suppliers unless otherwise stated. *1'H,3'H*-spiro[bicyclo[2.2.1]heptane-2,2'-perimidine][1], *N*-(2,6-dimethylphenyl)acetamide,[2] and *N*-(2,6-dimethylphenyl)acetimidoyl chloride[2] were synthesized following reported procedures. L and $AlMe_2L$ were prepared according to the method in the literature.[3] 1D and 2D NMR spectra were recorded with the following ^{1}H, and ^{13}C spectrometers: Bruker Avance II 300MHz, Avance III HD 400 MHz, and Avance I and II 600 MHz spectrometers. The chemical shift has been counted positively verse the low field and expressed in parts per million (ppm). The mass spectrometric analysis was done using three techniques, direct chemical ionization (DCI-CH_4) methods and recorded on a GCT Premier Waters mass spectrometer; electrospray ionization (ESI), recorded on a Waters Xevo G2 Q-TOF mass spectrometer; and a Maldi micro-MX micro–Mass in a pyrene matrix (ratio product/matrix:1/100). Single-crystal X-ray data were collected at low temperature (193(2)K) on a Bruker APEX II Quazar diffractometer equipped with a 30W air-cooled microfocus source [**LH(amidine-imine)** and **Al2Me2L2**] or on a Bruker D8 VENTURE diffractometer equipped with a PHOTON III detector [**LH(amidine-aminal)**, **AlMe2L**, **AlIMeL** and **AlI2L**], using MoK_α radiation (λ= 0.71037 Å) or CuK_α radiation (λ= 1.54178 Å). Phi and Omega scans were performed for data collection and an empirical absorption correction was applied[4]. The structure was solved by the intrinsic phasing method (ShelXT)[5] and refined by the full-matrix least-squares method on F^2 [6]. All non-hydrogen atoms were refined with anisotropic displacement parameters, while hydrogen atoms were refined isotropically at calculated positions using a riding model, except the N-bound hydrogen atoms located in difference Fourier maps and refined freely for **LH(amidine-imine)** or with $U_{iso}(H) = 1.2U_{eq}(N)$ for **LH(amidine-aminal)**.

(1) Near Infrared Ray Absorber with Excellent Absorbing Ability in near IR Region, Heat Resistance and Light Fastness for near-IR Cut-off Filter and Manufacturing Method Thereof. JP2017165857, September 21, 2017.

(2) Boeré, R. T.; Klassen, V.; Wolmershäuser, G. Synthesis of Some Very Bulky N,N′-Disubstituted Amidines and Initial Studies of Their Coordination Chemistry†. *Journal of the Chemical Society, Dalton Transactions* **1998**, No. 24, 4147–4154. https://doi.org/10.1039/a805548c.

(3) Saltarini, S.; Villegas-Escobar, N.; Martínez, J.; Daniliuc, C. G.; Matute, R. A.; Gade, L. H.; Rojas, R. S. Toward a Neutral Single-Component Amidinate Iodide Aluminum Catalyst for the CO_2 Fixation into Cyclic Carbonates. *Inorg Chem* **2021**, *60* (2), 1172–1182. https://doi.org/10.1021/acs.inorgchem.0c03290.

(4) Bruker, SADABS, Bruker AXS Inc., Madison, Wisconsin, USA, 2008.

(5) Sheldrick, G. M. Crystal Structure Refinement with *SHELXL*. *Acta Crystallogr C Struct Chem* **2015**, *71* (1), 3–8. https://doi.org/10.1107/S2053229614024218.

(6) Sheldrick, G. M. *SHELXT* – Integrated Space-Group and Crystal-Structure Determination. *Acta Crystallogr A Found Adv* **2015**, *71* (1), 3–8. https://doi.org/10.1107/S2053273314026370.

(7) Hohenberg, P.; Kohn, W. Inhomogeneous Electron Gas. *Physical Review* **1964**, *136* (3B), B864–B871. https://doi.org/10.1103/PhysRev.136.B864.

(8) Kohn, W.; Sham, L. J. Self-Consistent Equations Including Exchange and Correlation Effects. *Physical Review* **1965**, *140* (4A), A1133–A1138. https://doi.org/10.1103/PhysRev.140.A1133.

(9) Frisch, M. J.; Trucks, G. W.; Schlegel, H. B.; Scuseria, G. E.; Robb, M. A.; Cheeseman, J. R.; Scalmani, G.; Barone, V.; Petersson, G. A.; Nakatsuji, H.; Li, X.; Caricato, M.; Marenich, A. V.; Bloino, J.; Janesko, B. G.; Gomperts, R.; Mennucci, R.; Hratchian, H. P.; Ortiz, J. V.; Izmaylov, A. F.; Sonnenberg, J. L.; Williams-Young, D.; Ding, F.; Lipparini, F.; Egidi, F.; Goings, J.; Peng, B.; Petrone, A.; Henderson, T.; Ranasinghe, D.; Zakrzewski, V. G.; Gao, J.; Rega, N.; Zheng, G.; Liang, W.; Hada, M.; Ehara, M.; Toyota, K.; Fukuda, R.; Hasegawa, J.; Ishida, M.; Nakajima, T.; Honda, Y.; Kitao, O.; Nakai, H.; Vreven, T.; Throssel, K.; Montgomery, J. A.; Jr.; Peralta, J. E.; Ogliar, F.; Bearpark, M. J.; Heyd, J. J.; Brothers, E. N.; Kudin, K. N.; Staroverov, V. N.; Keith, T. A.; Kobayashi, R.; Normand, J.; Raghavachari, K.; Rendell, A. P.; Burant, J. C.; Iyengar, S. S.; Tomasi, J.; Cossi, M.; Millam, J. M.; Klene, M.; Adamo, C.; Cammi, R.; Ochterski, J. W.; Martin, R. L.; Morokuma, K.; Farkas, O.; Foresman, J. B.; Fox, D. J. Gaussian 16, Revision C.01; Gaussian Inc., Wallingford, CT, 2016.

(10) Zhao, Y.; Truhlar, D. G. The M06 Suite of Density Functionals for Main Group Thermochemistry, Thermochemical Kinetics, Noncovalent Interactions, Excited States, and Transition Elements: Two New Functionals and Systematic Testing of Four M06-Class Functionals and 12 Other Functionals. *Theor Chem Acc* **2008**, *120* (1–3), 215–241. https://doi.org/10.1007/s00214-007-0310-x.

(11) Hehre, W. J.; Ditchfield, R.; Pople, J. A. Self—Consistent Molecular Orbital Methods. XII. Further Extensions of Gaussian—Type Basis Sets for Use in Molecular Orbital Studies of Organic Molecules. *J Chem Phys* **1972**, *56* (5), 2257–2261. https://doi.org/10.1063/1.1677527.

(12) Hariharan, P. C.; Pople, J. A. The Influence of Polarization Functions on Molecular Orbital Hydrogenation Energies. *Theor Chim Acta* **1973**, *28* (3), 213–222. https://doi.org/10.1007/BF00533485.

(13) Hay, P. J.; Wadt, W. R. *Ab Initio* Effective Core Potentials for Molecular Calculations. Potentials for the Transition Metal Atoms Sc to Hg. *J Chem Phys* **1985**, *82* (1), 270–283. https://doi.org/10.1063/1.448799.

(14) Wadt, W. R.; Hay, P. J. *Ab Initio* Effective Core Potentials for Molecular Calculations. Potentials for Main Group Elements Na to Bi. *J Chem Phys* **1985**, *82* (1), 284–298. https://doi.org/10.1063/1.448800.

(15) McLean, A. D.; Chandler, G. S. Contracted Gaussian Basis Sets for Molecular Calculations. I. Second Row Atoms, *Z* =11–18. *J Chem Phys* **1980**, *72* (10), 5639–5648. https://doi.org/10.1063/1.438980.

(16) Krishnan, R.; Binkley, J. S.; Seeger, R.; Pople, J. A. Self-Consistent Molecular Orbital Methods. XX. A Basis Set for Correlated Wave Functions. *J Chem Phys* **1980**, *72* (1), 650–654. https://doi.org/10.1063/1.438955.

(17) Clark, T.; Chandrasekhar, J.; Spitznagel, G. W.; Schleyer, P. V. R. Efficient Diffuse Function-augmented Basis Sets for Anion Calculations. III. The 3-21+G Basis Set for First-row Elements, Li–F. *J Comput Chem* **1983**, *4* (3), 294–301. https://doi.org/10.1002/jcc.540040303.

(18) Peterson, K. A.; Figgen, D.; Goll, E.; Stoll, H.; Dolg, M. Systematically Convergent Basis Sets with Relativistic Pseudopotentials. II. Small-Core Pseudopotentials and Correlation Consistent Basis Sets for the Post- *d* Group 16–18 Elements. *J Chem Phys* **2003**, *119* (21), 11113–11123. https://doi.org/10.1063/1.1622924.

(19) Miertuš, S.; Scrocco, E.; Tomasi, J. Electrostatic Interaction of a Solute with a Continuum. A Direct Utilizaion of AB Initio Molecular Potentials for the Prevision of Solvent Effects. *Chem Phys* **1981**, *55* (1), 117–129. https://doi.org/10.1016/0301-0104(81)85090-2.

(20) Barone, V.; Cossi, M. Quantum Calculation of Molecular Energies and Energy Gradients in Solution by a Conductor Solvent Model. *J Phys Chem A* **1998**, *102* (11), 1995–2001. https://doi.org/10.1021/jp9716997.

(21) Kulkarni, A.; Arumugam, S.; Francis, M.; Reddy, P. G.; Nag, E.; Gorantla, S. M. N. V. T.; Mondal, K. C.; Roy, S. Solid-State Isolation of Cyclic Alkyl(Amino) Carbene (CAAC)-Supported Structurally Diverse Alkali Metal-Phosphinidenides. *Chemistry – A European Journal* **2021**, *27* (1), 200–206. https://doi.org/10.1002/chem.202003505.

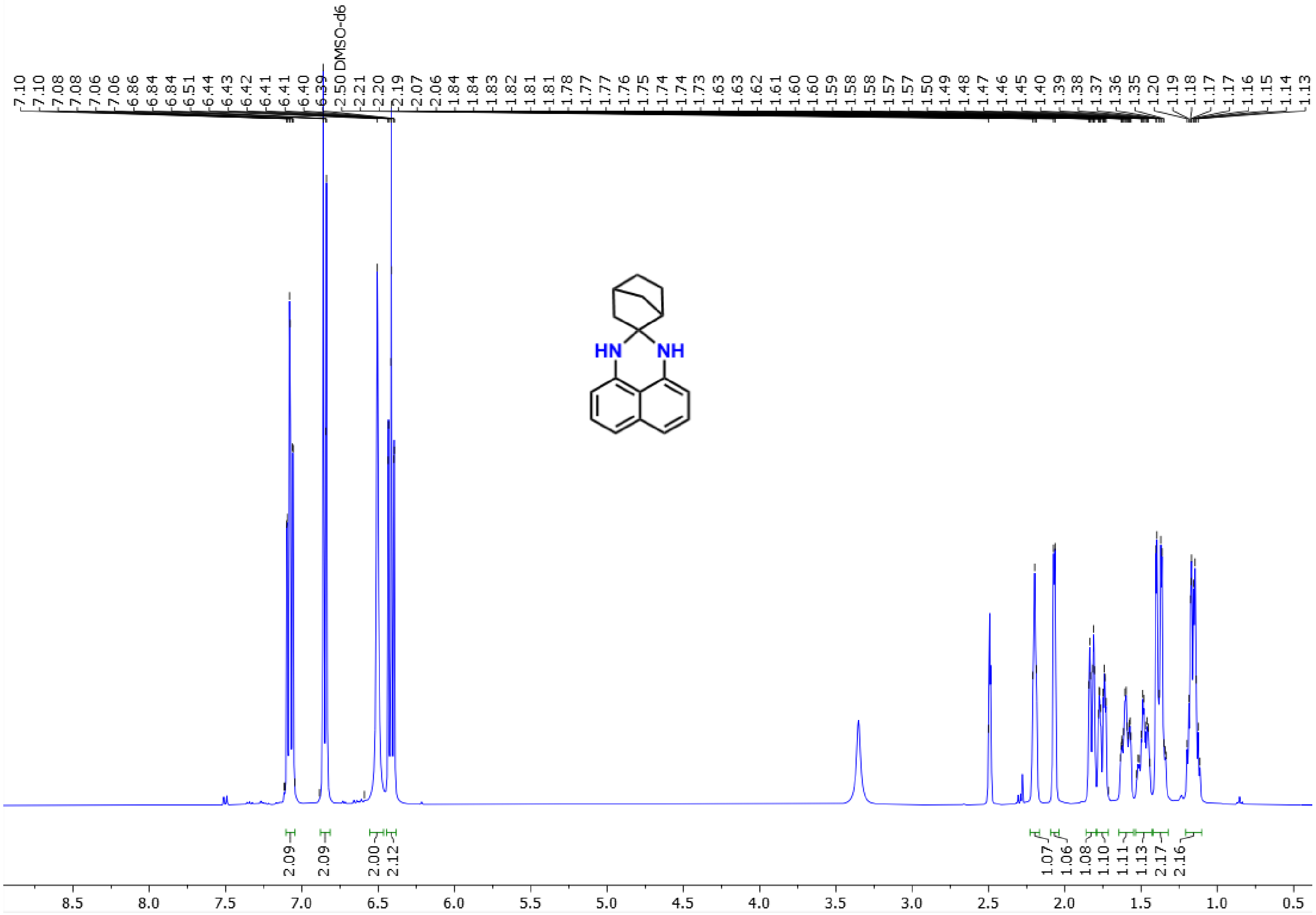

Figure S1. ^{1}H NMR spectrum of aminal precursor (DMSO-d_6, 400 MHz)

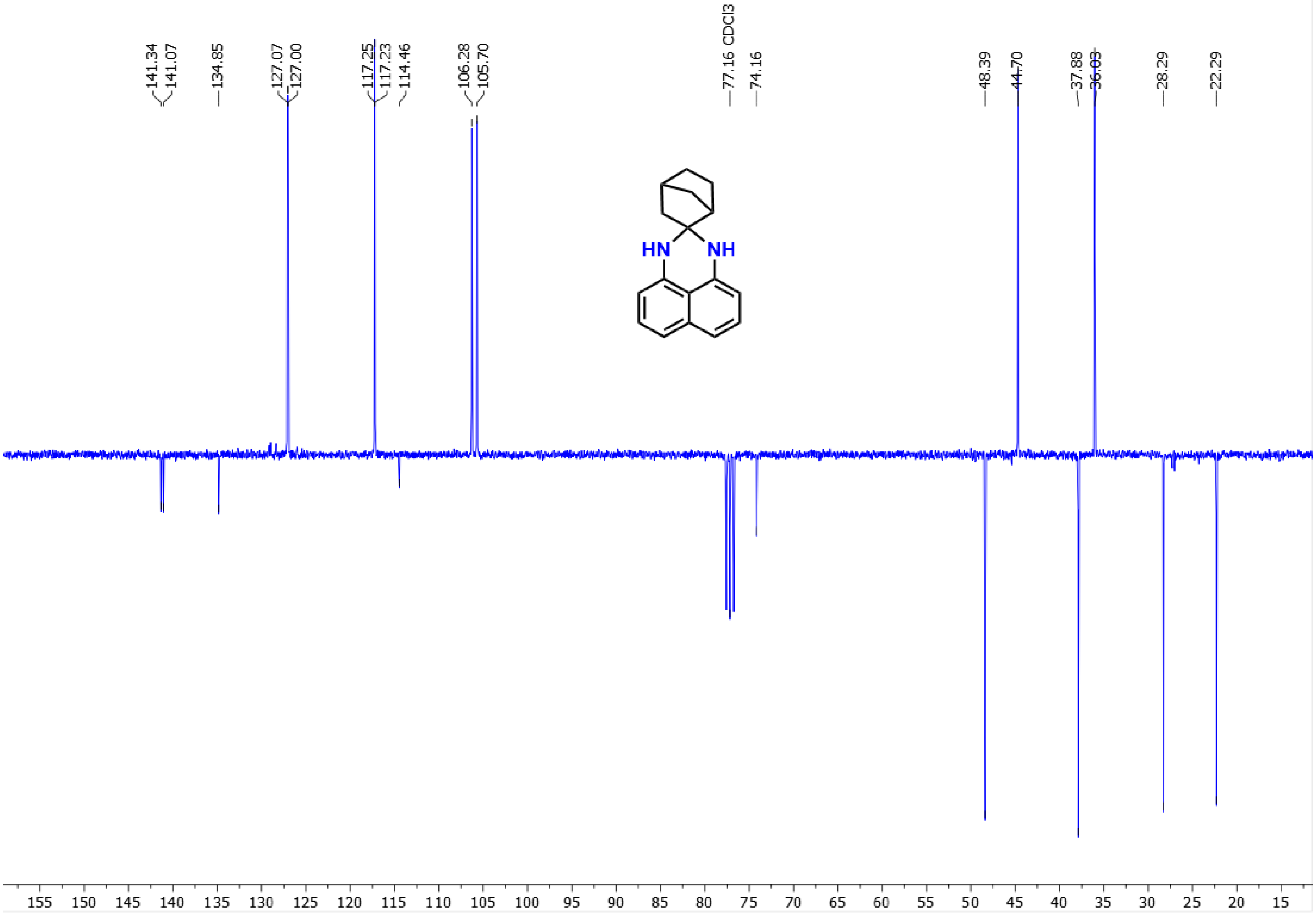

Figure S2. $^{13}C\{^{1}H\}$ NMR spectrum of aminal precursor (Chloroform-d_1, 125 MHz)

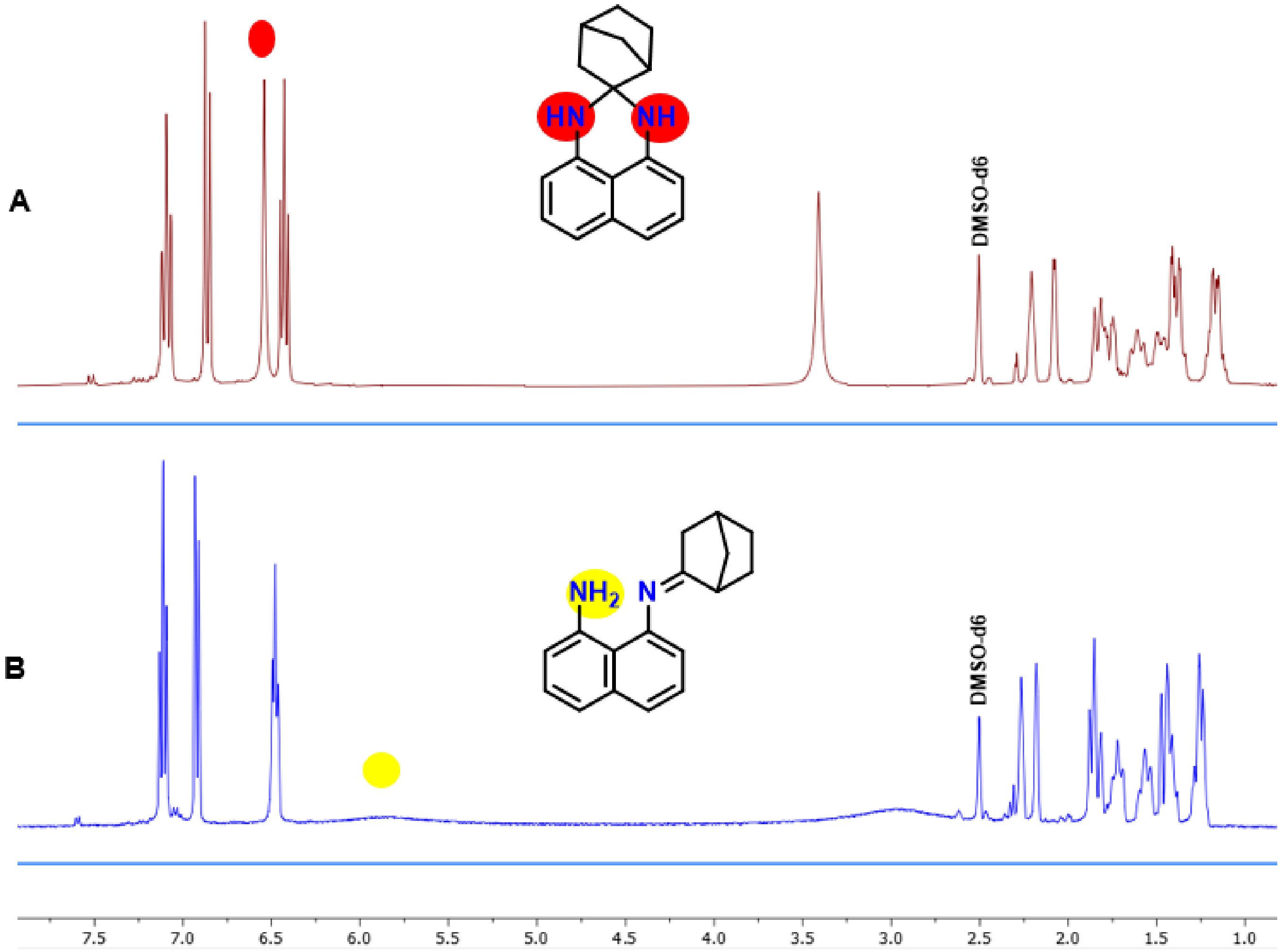

Figure S3. Aminal - amino-imine equilibrium. **A** = ^{1}H-NMR at 20°C, **B** = ^{1}H-NMR at 130°C

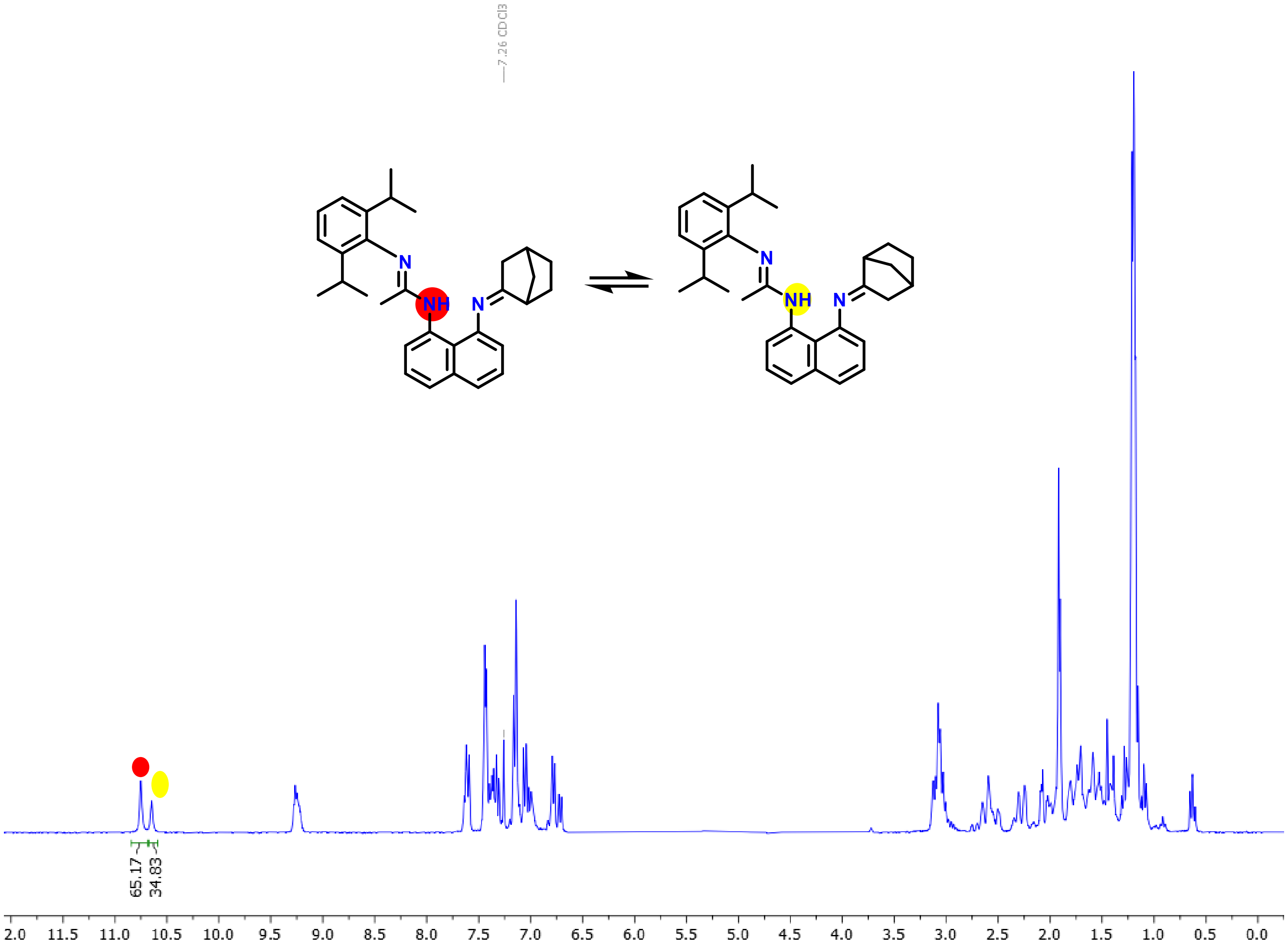

Figure S4. E/Z amidine-imine relation

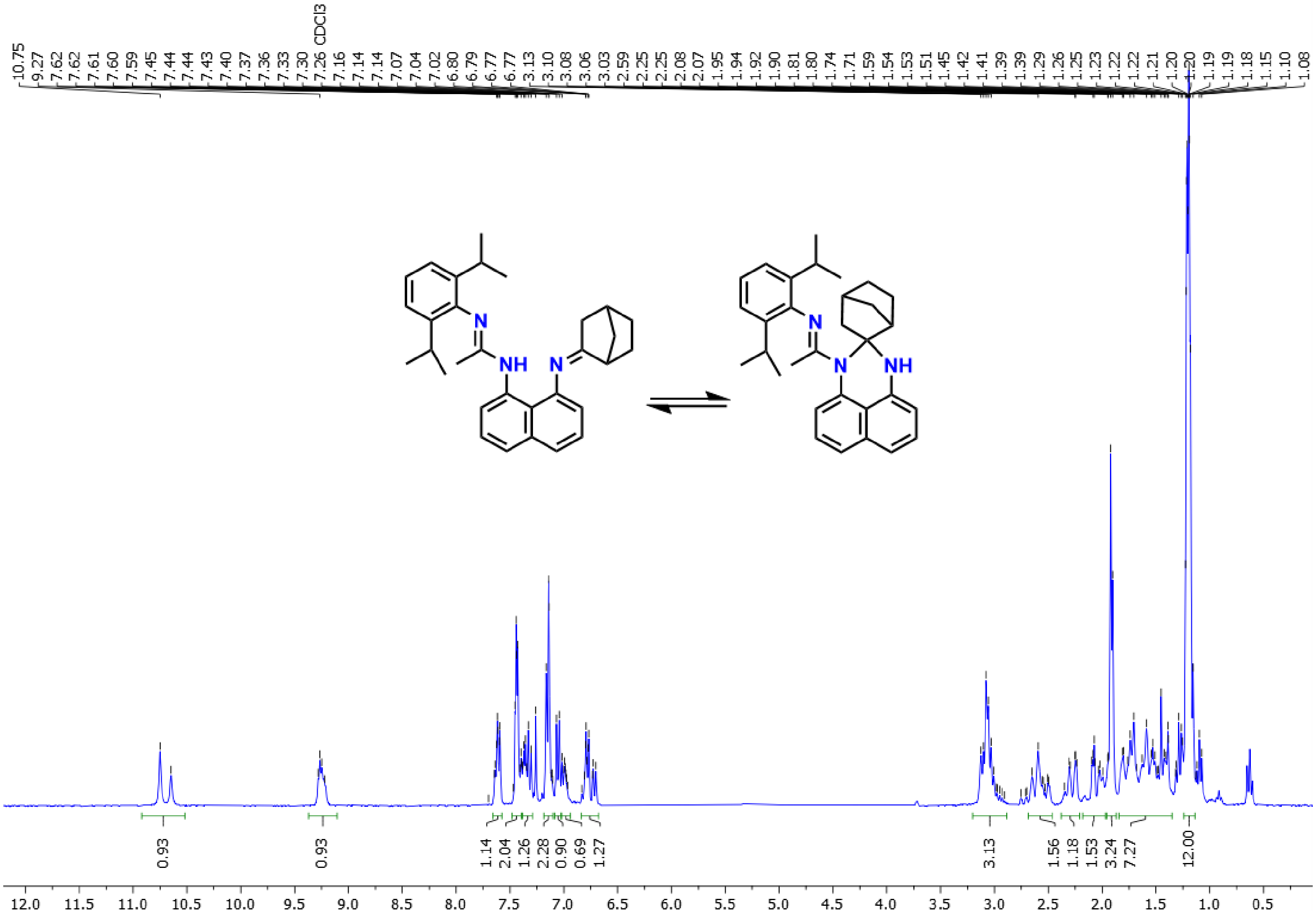

Figure S5. ^{1}H NMR spectrum of **LH** (Chloroform-d_1, 400 MHz)

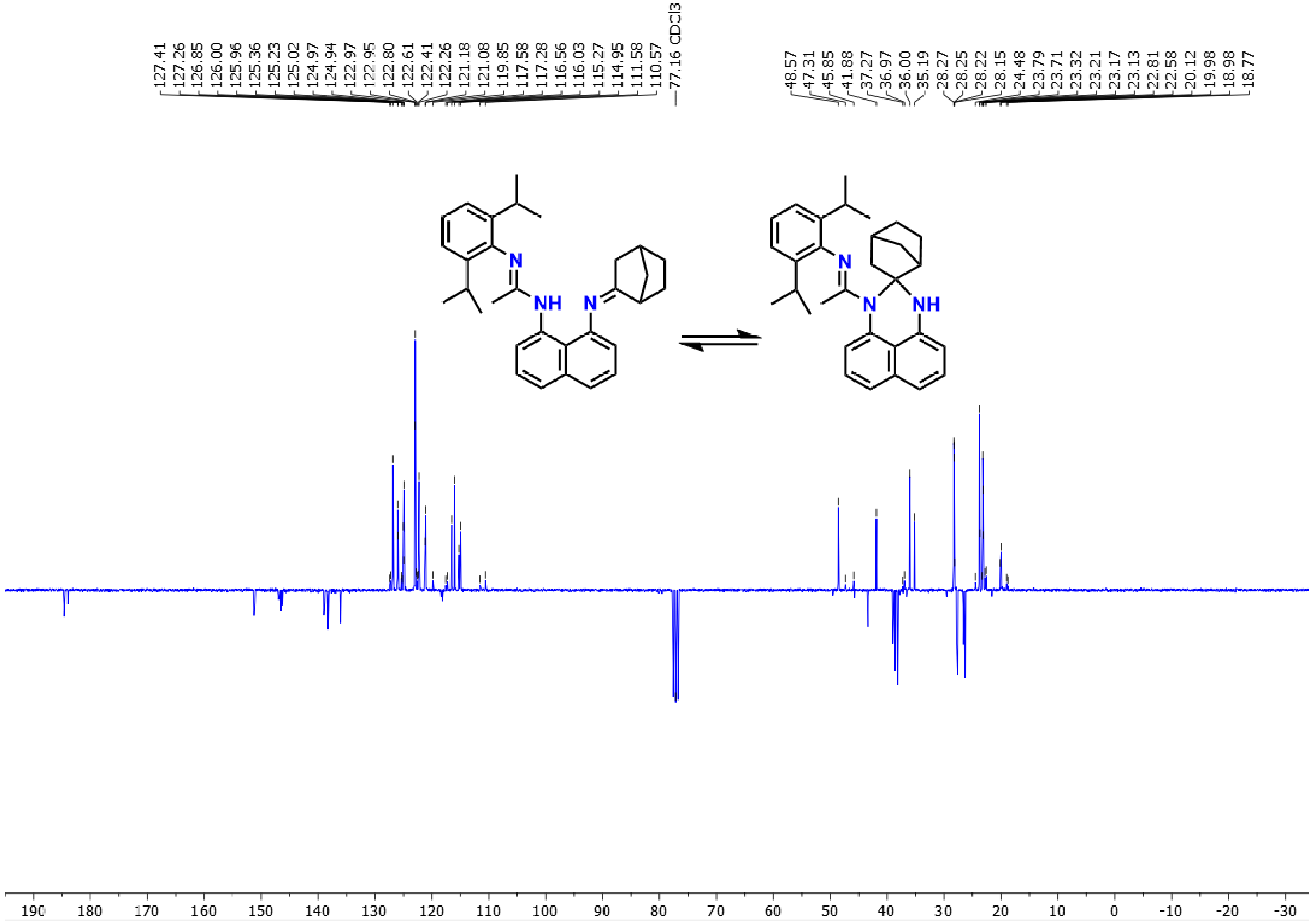

Figure S6. $^{13}C\{^{1}H\}$ NMR spectrum of **LH** (Chloroform-d_1, 101 MHz)

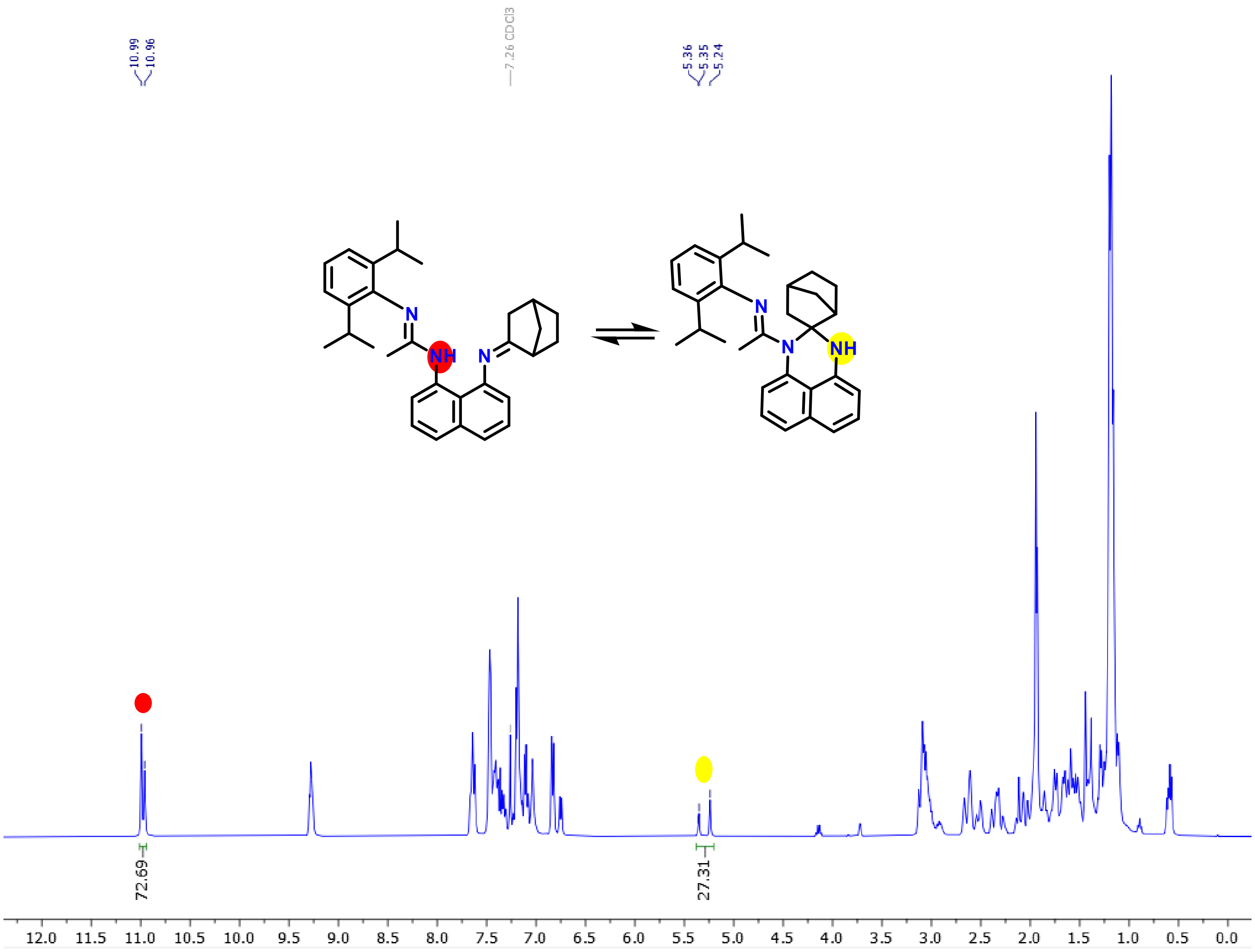

Figure S7. ^{1}H NMR spectrum of **LH** at -50 °C (Chloroform-d_1, 101 MHz)

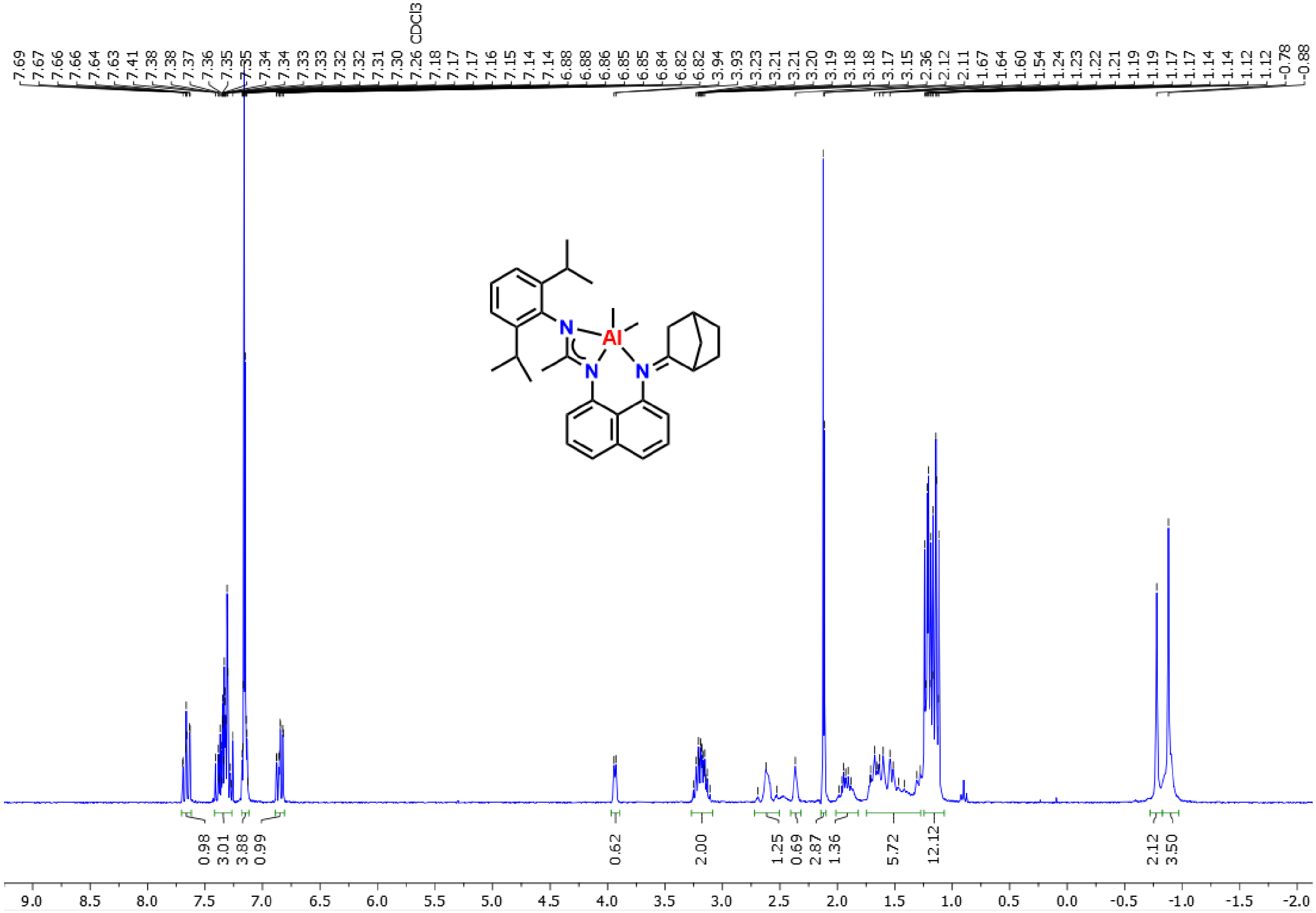

Figure S8. ^{1}H NMR spectrum of **AlMe$_2$L** (Chloroform-d$_1$, 400 MHz)

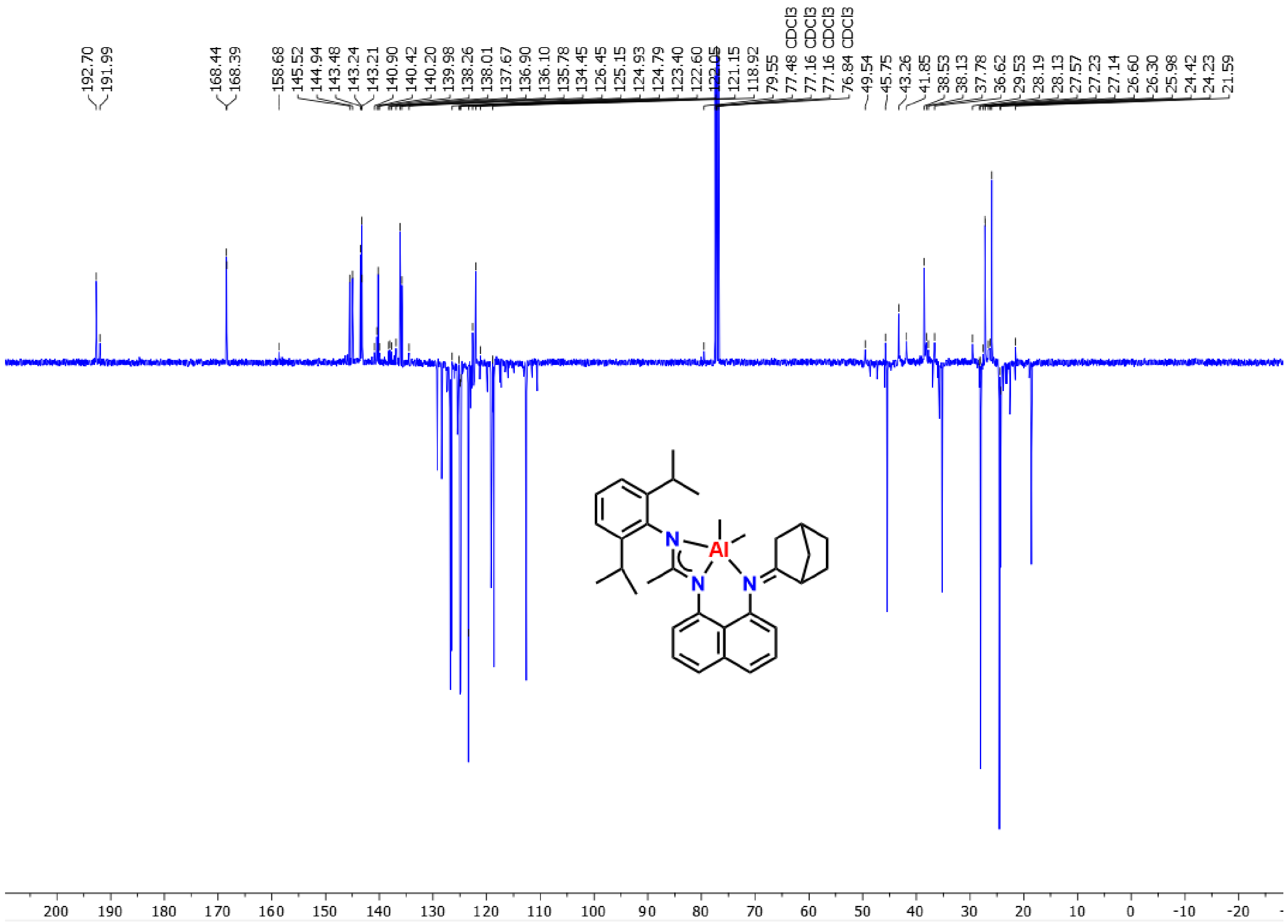

Figure S9. $^{13}C\{^{1}H\}$ NMR spectrum of **$AlMe_2L$** (Chloroform-d_1, 101 MHz)

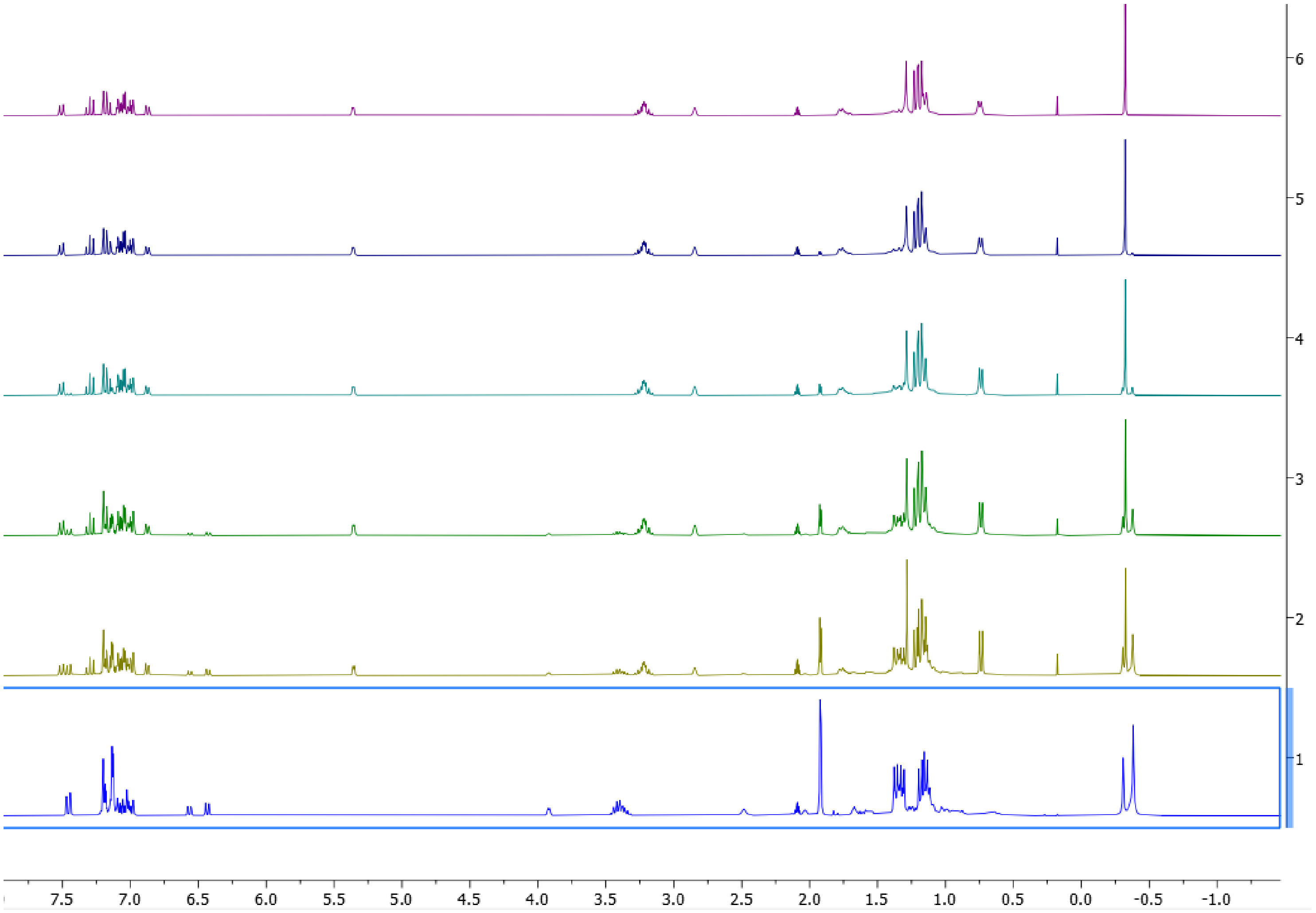

Figure S10. Comparison of ^{1}H-NMR spectrum of **AlMe$_2$L** heated at 100 °C for 6 d (Toluene-d$_8$, 300 MHz). 1- t = 0 d. 2- t = 1 d. 3- t = 2 d. 4- t = 4 d. 5- t = 5 d. 6- t = 6 d

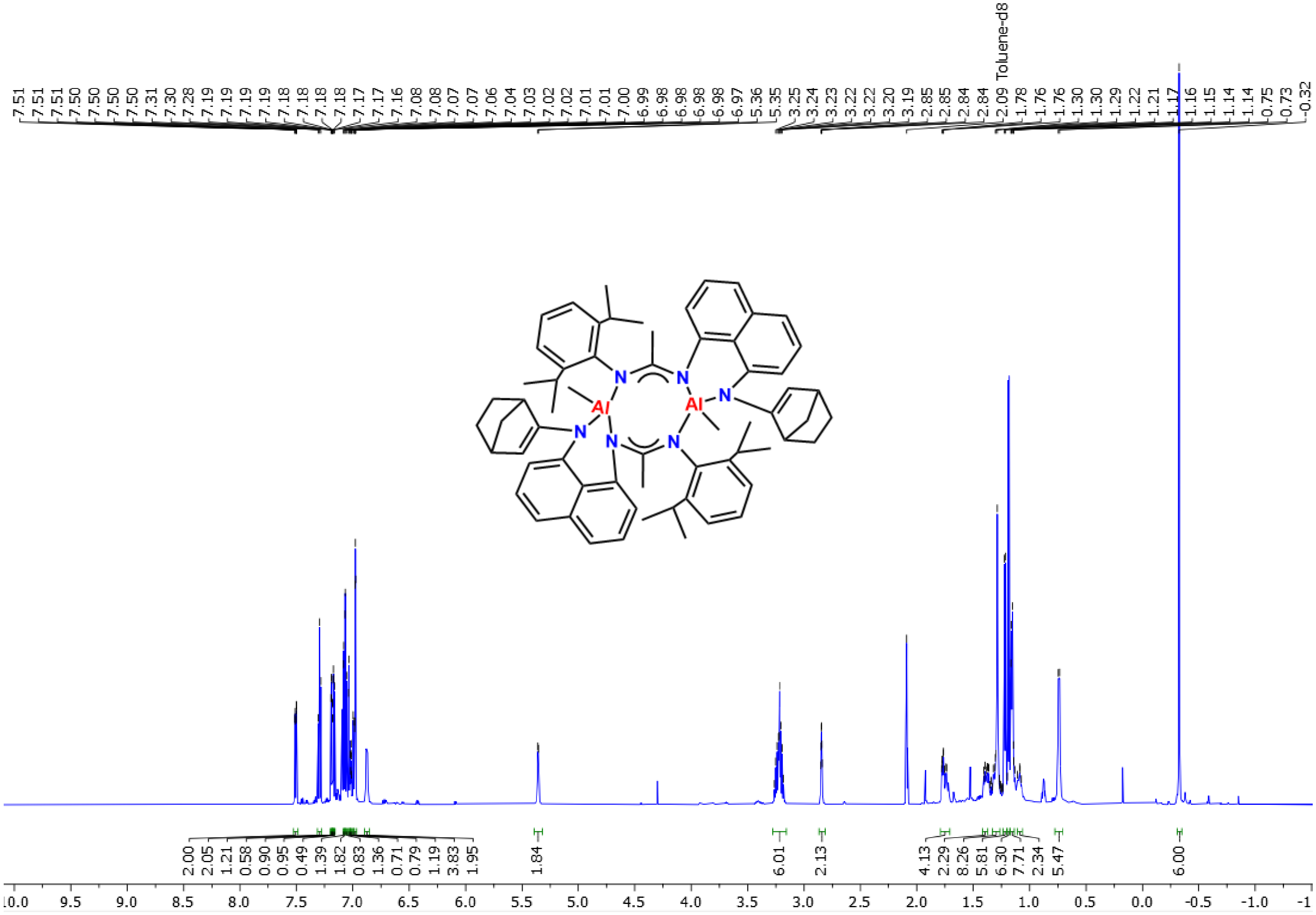

Figure S11. ^{1}H NMR spectrum of **$Al_2Me_2L_2$** (Toluene-d_8, 600 MHz)

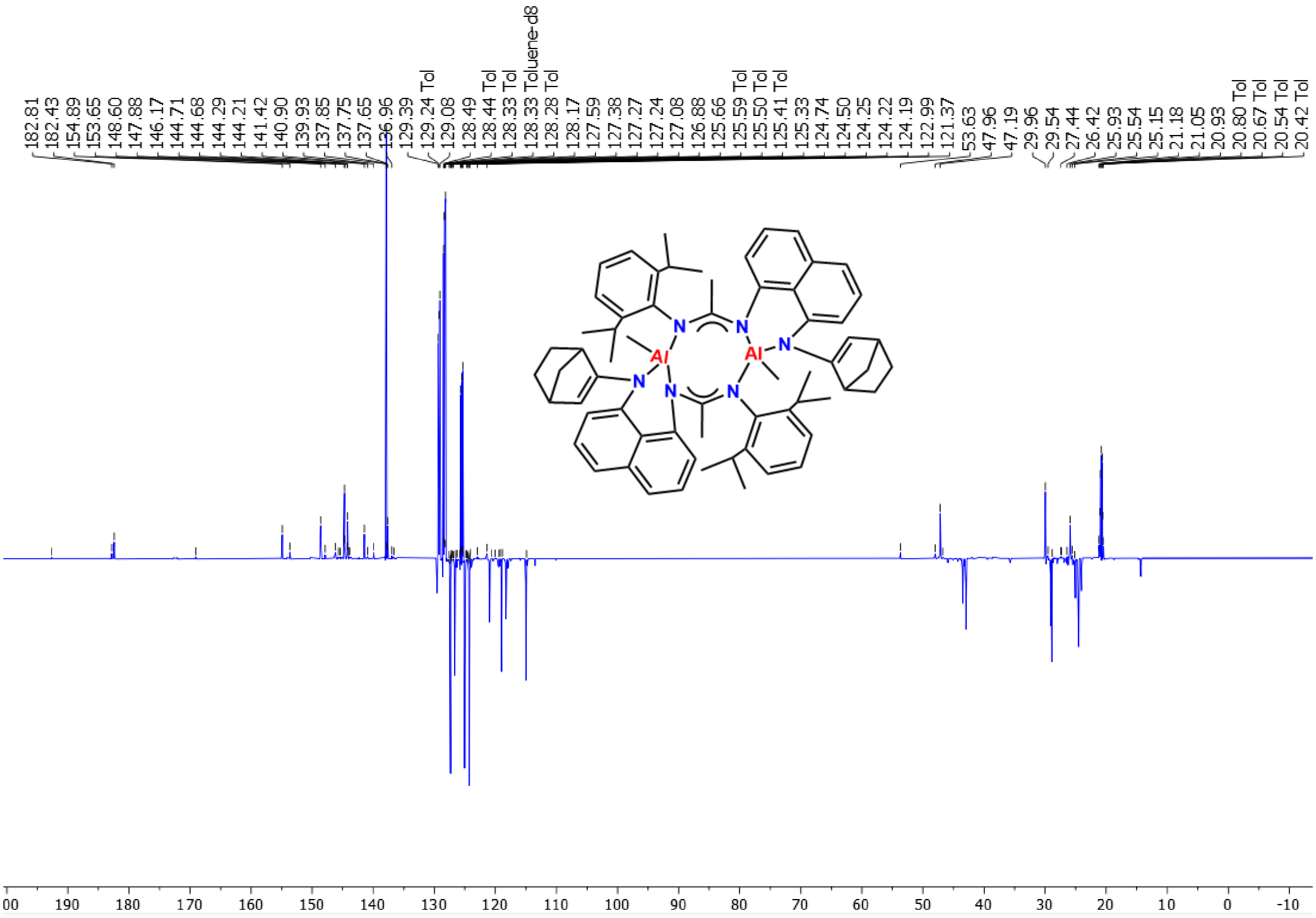

Figure S12. $^{13}C\{^{1}H\}$ NMR spectrum of **$Al_2Me_2L_2$** (Toluene-d_8, 151 MHz)

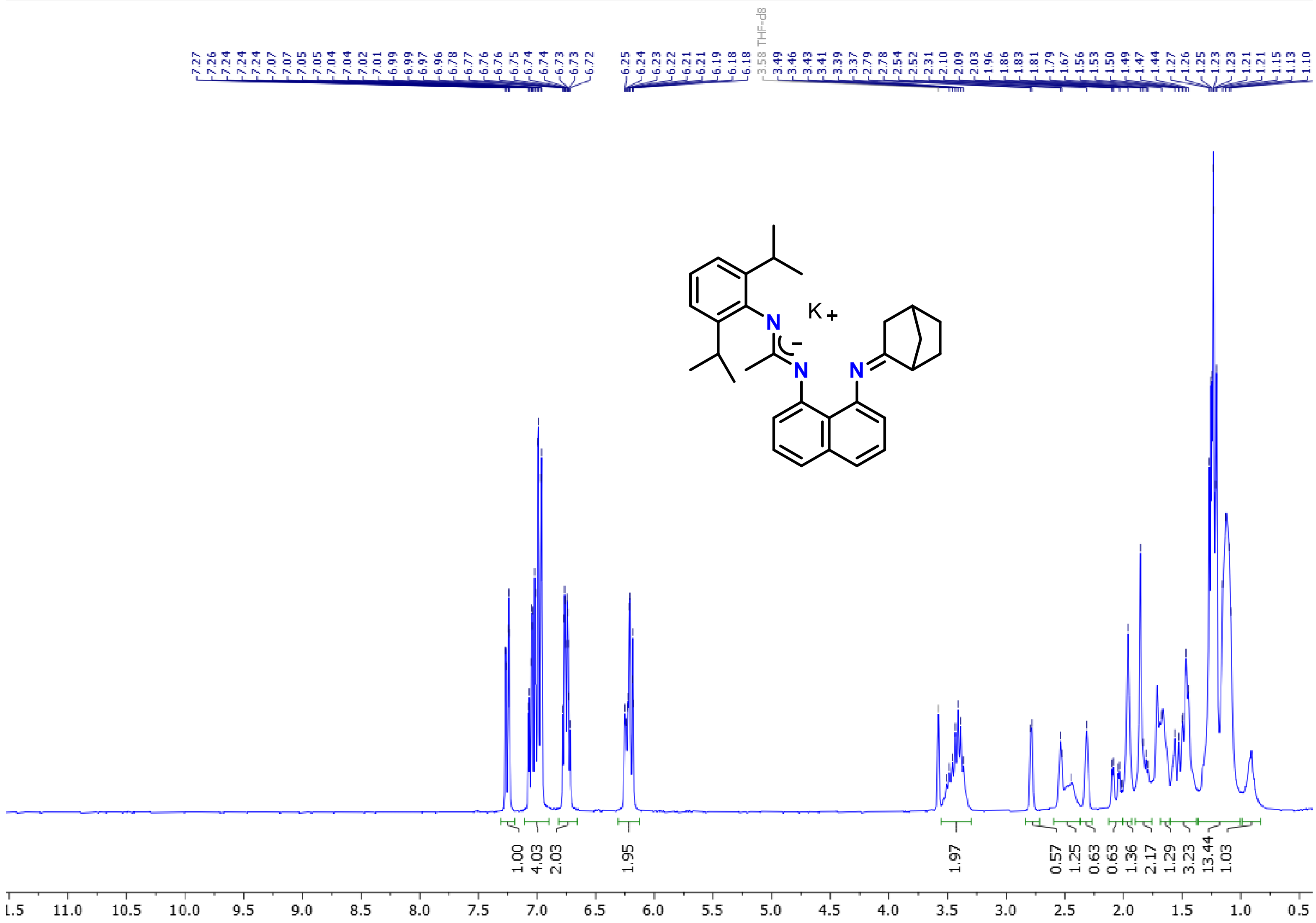

Figure S13. ^{1}H NMR spectrum of **LK** (THF-d_8, 300 MHz)

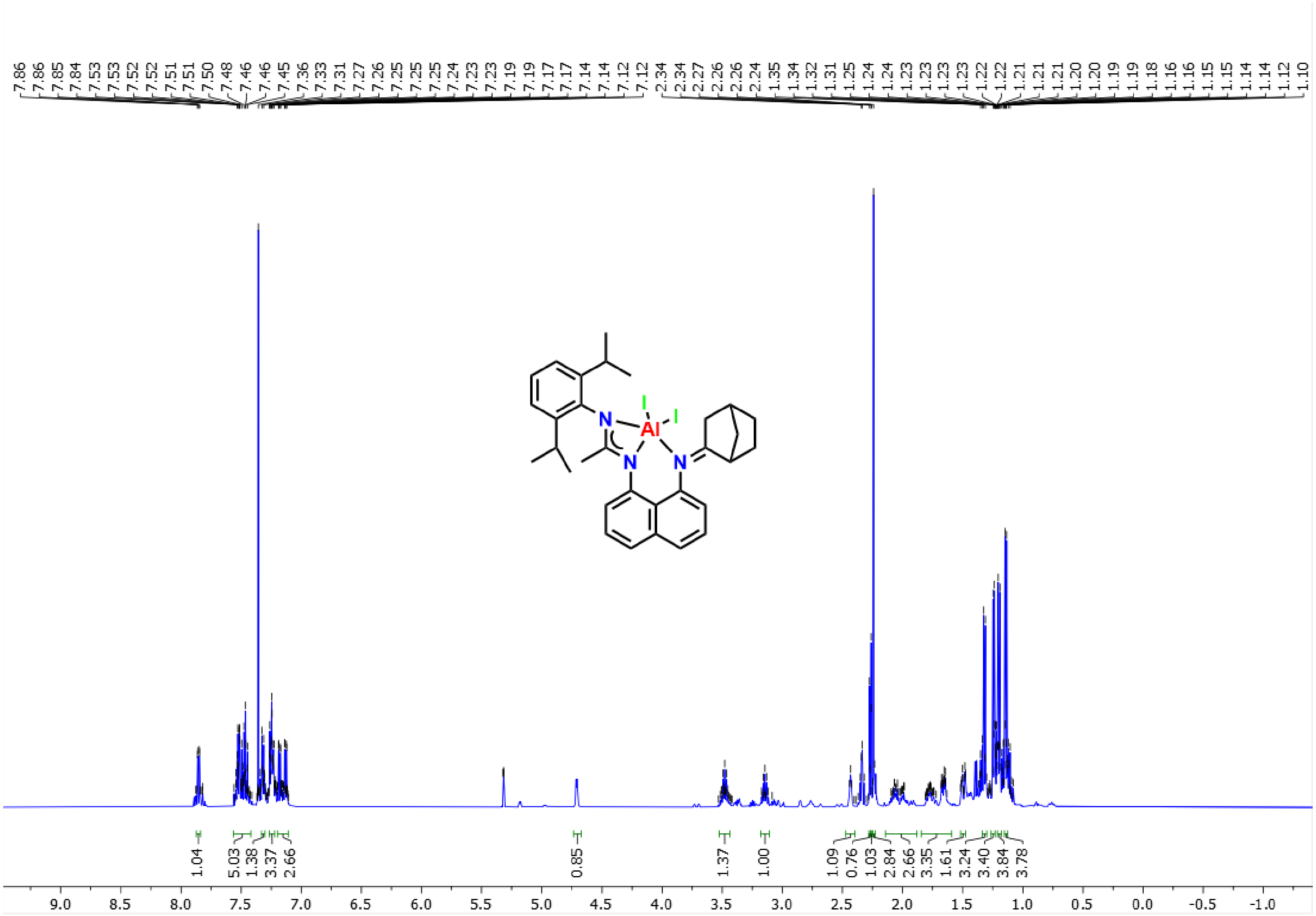

Figure S14. ^{1}H NMR spectrum of **AlI$_2$L** (Dichloromethane-d$_2$, 400 MHz)

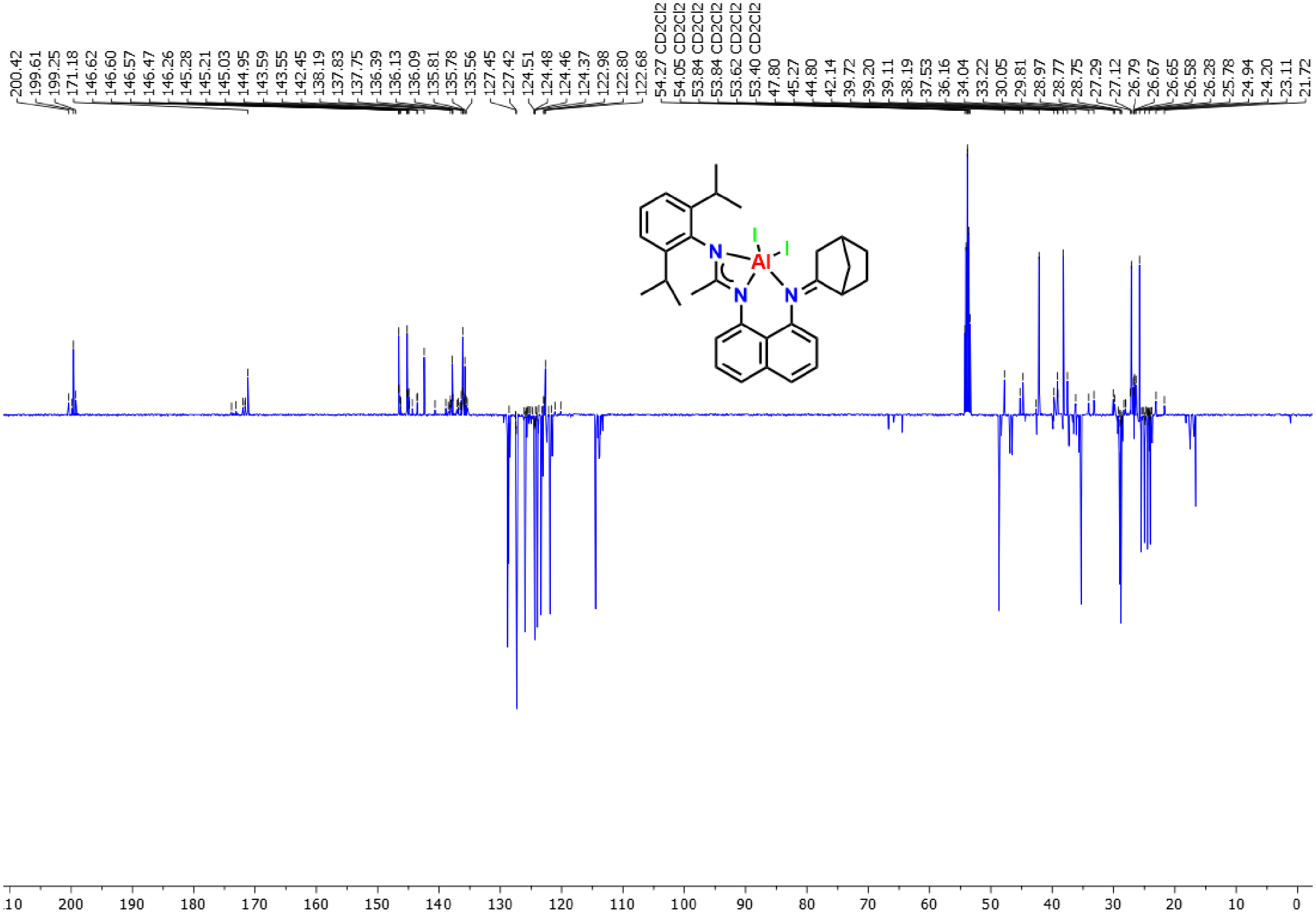

Figure S15. $^{13}C\{^{1}H\}$ NMR spectrum of **AlI_2L** (Dichloromethane-d_2, 126 MHz)

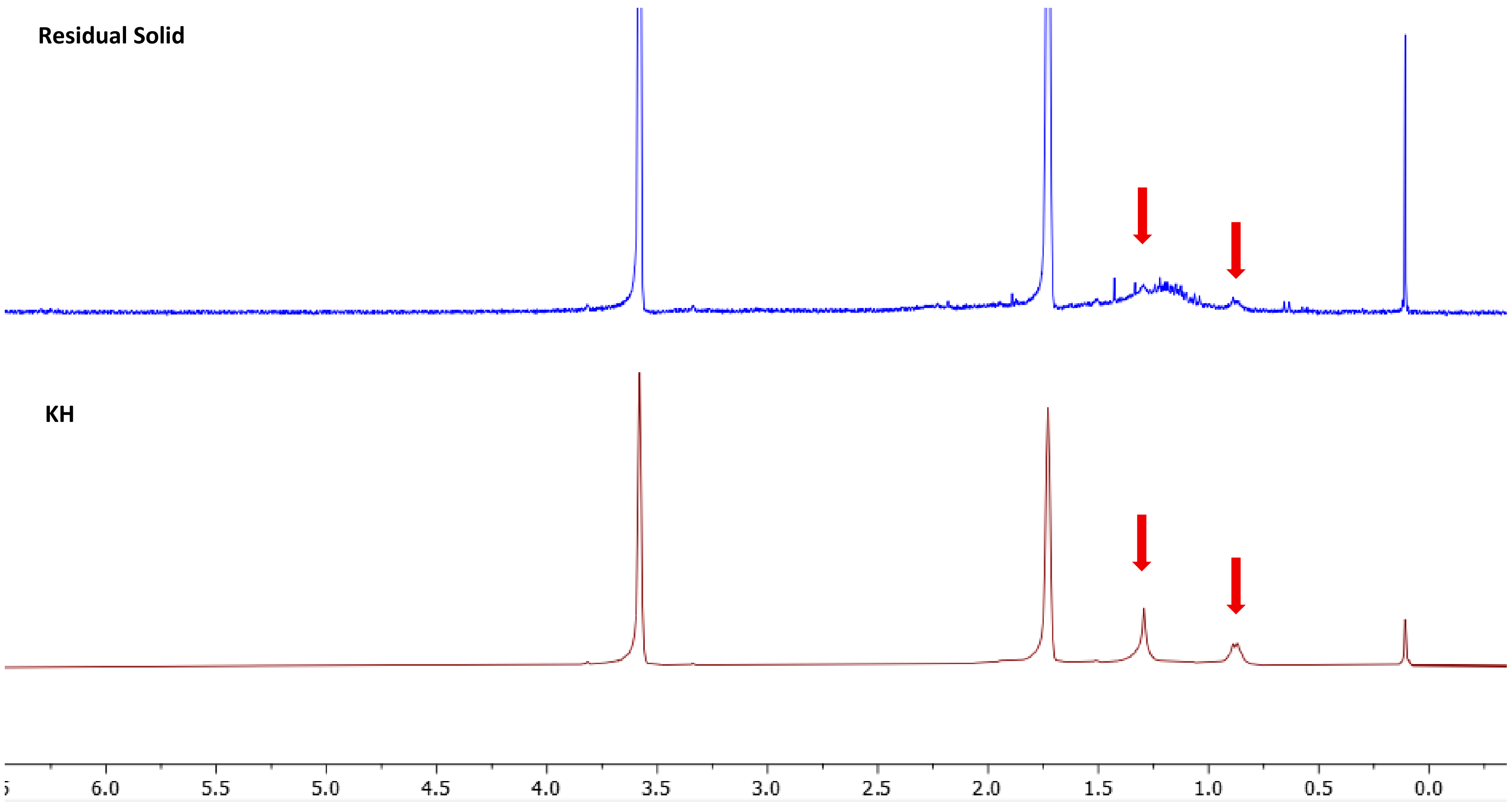

Figure S16. ^{1}H-NMR comparison between residual solid in the synthesis of **AlI$_2$L** and KH in THF-d_8.

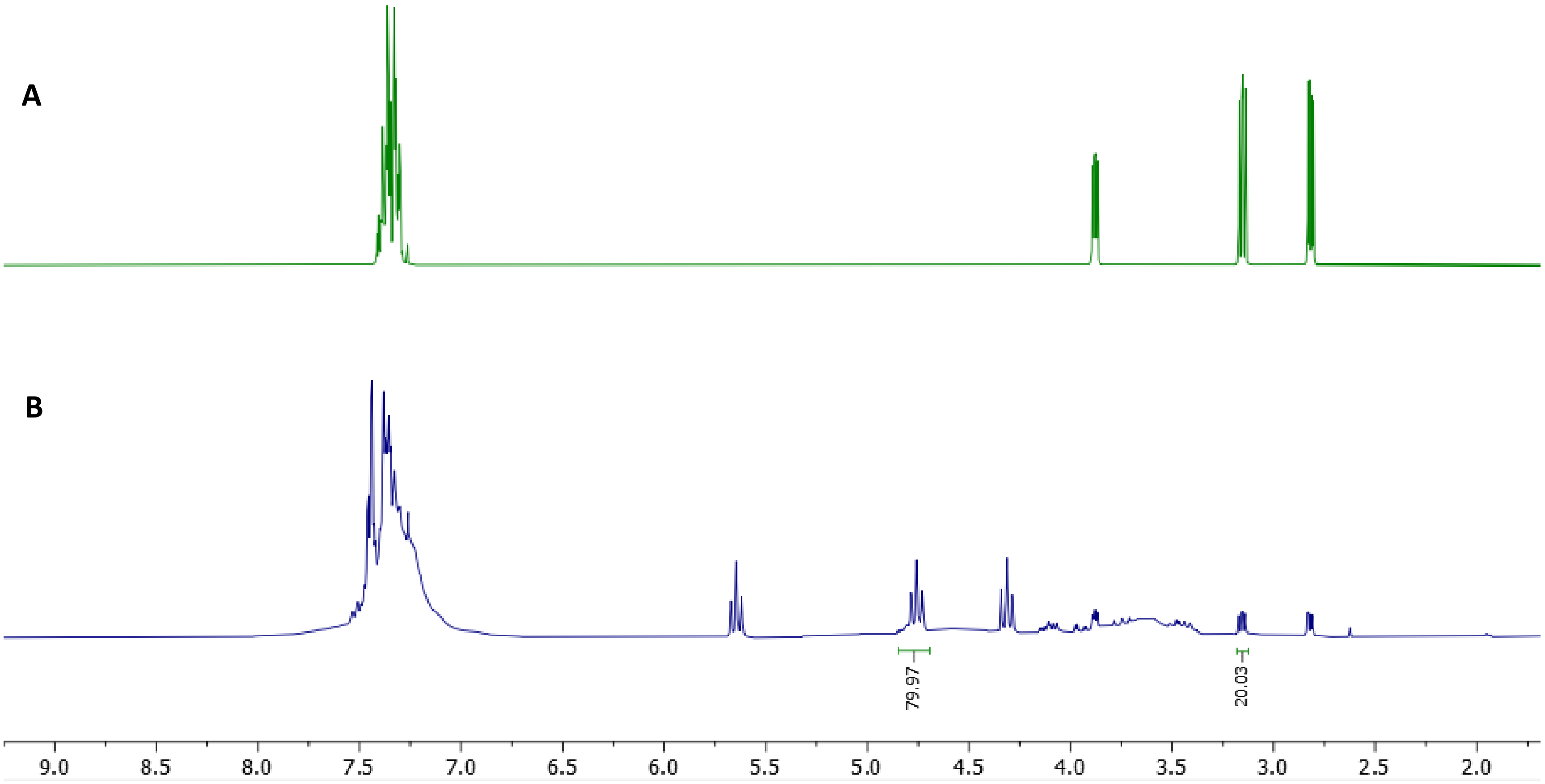

Figure S17. **A**) ^{1}H-NMR pure styrene oxide in chloroform-d_1. **B**) ^{1}H-NMR in chloroform-d_1 of styrene carbonate and styrene oxide mixture after 24 h at 90 °C, 5 bar of CO_2, and 1.5 mol% of **AlI₂L**.

Figure S18. Mass Analysis

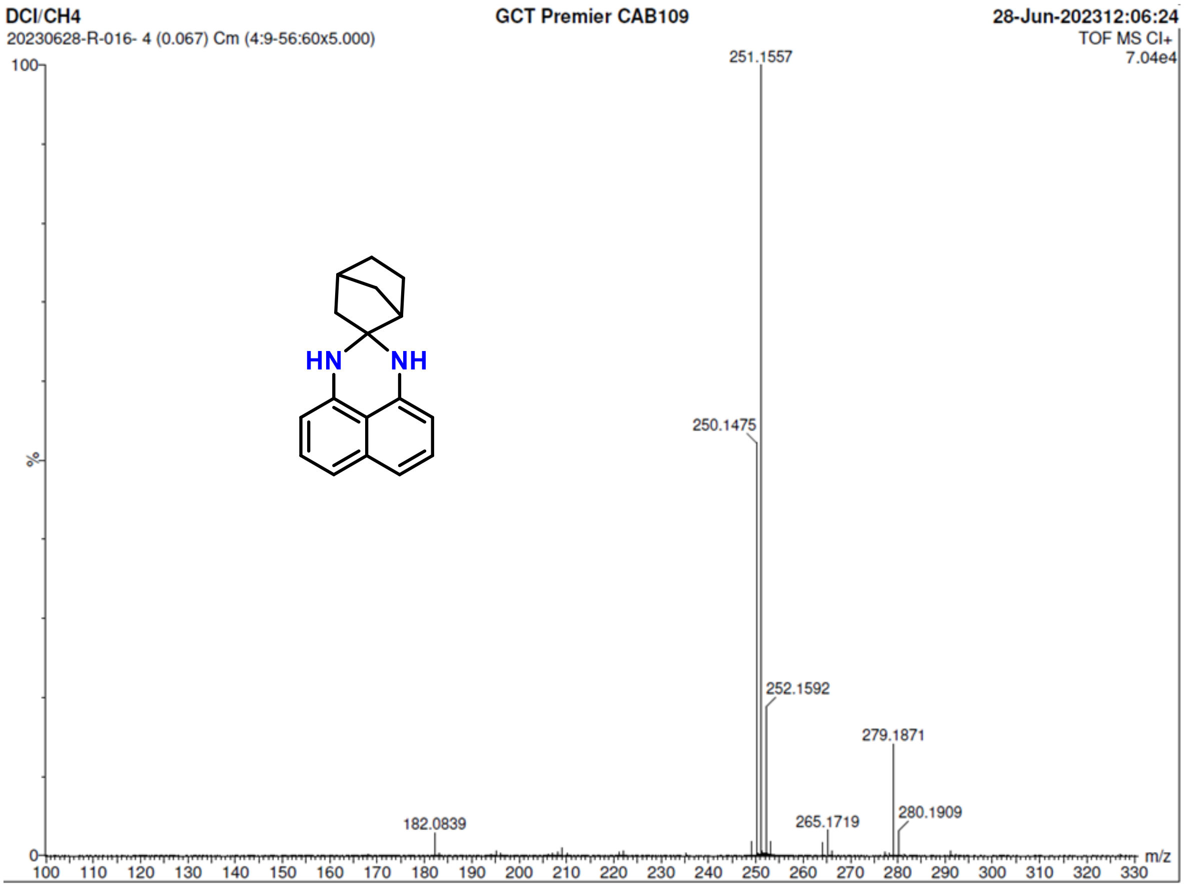

HRMS (DCI-CH_4) m/z: 251.1557 ([M + H]+) calcd for $C_{17}H_{19}N_2$+ 251.1543

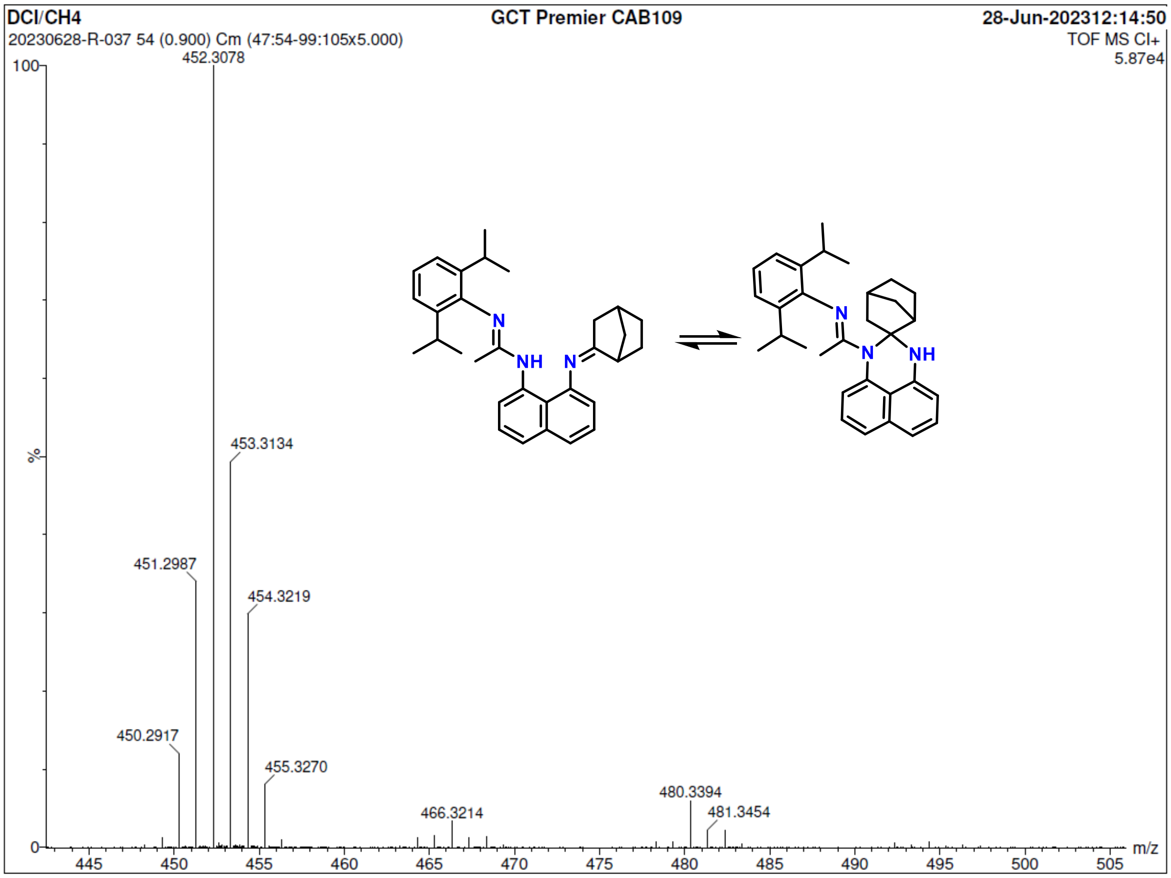

HRMS (DCI-CH_4) m/z: 452.3078 ([M + H]+) calcd for $C_{31}H_{38}N_3$+ 452.3066.

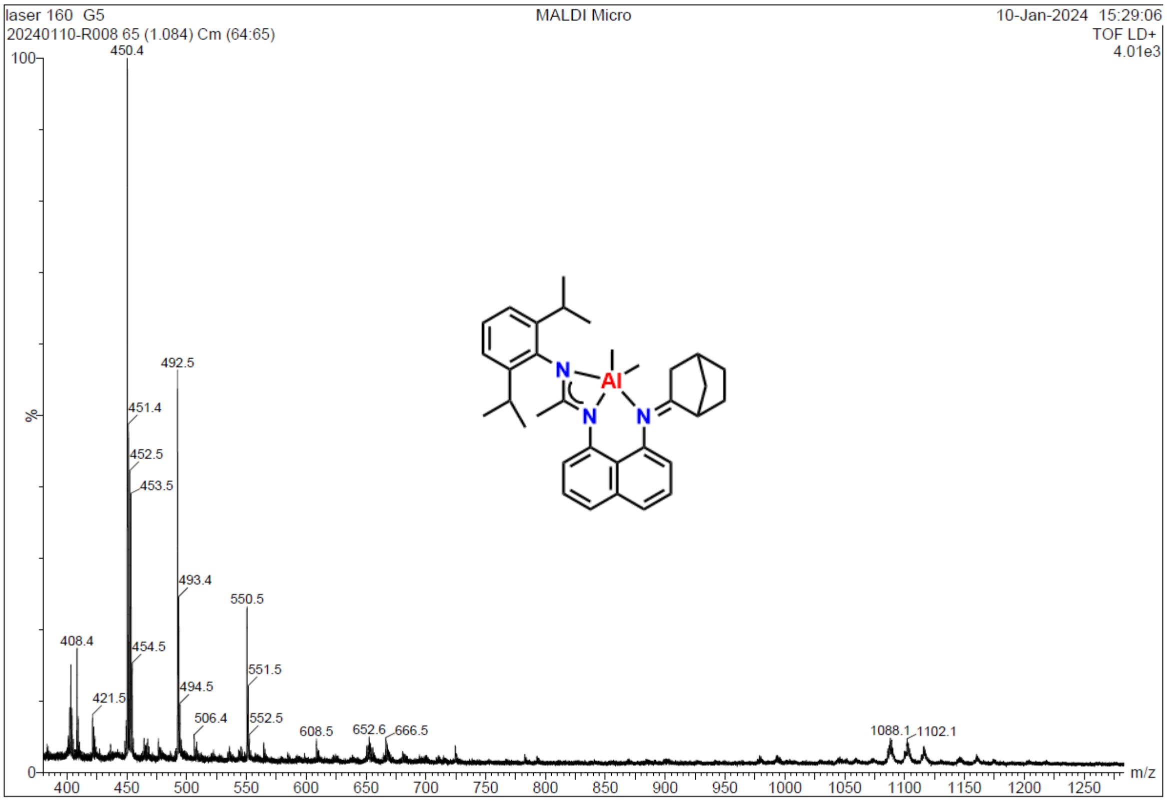

MS (Maldi-TOF) m/z: 507.3 ([M]$^+$), 492.5 ([M - CH_3]$^+$), 450.4 ([M - Al(CH_3)$_2$]$^+$).

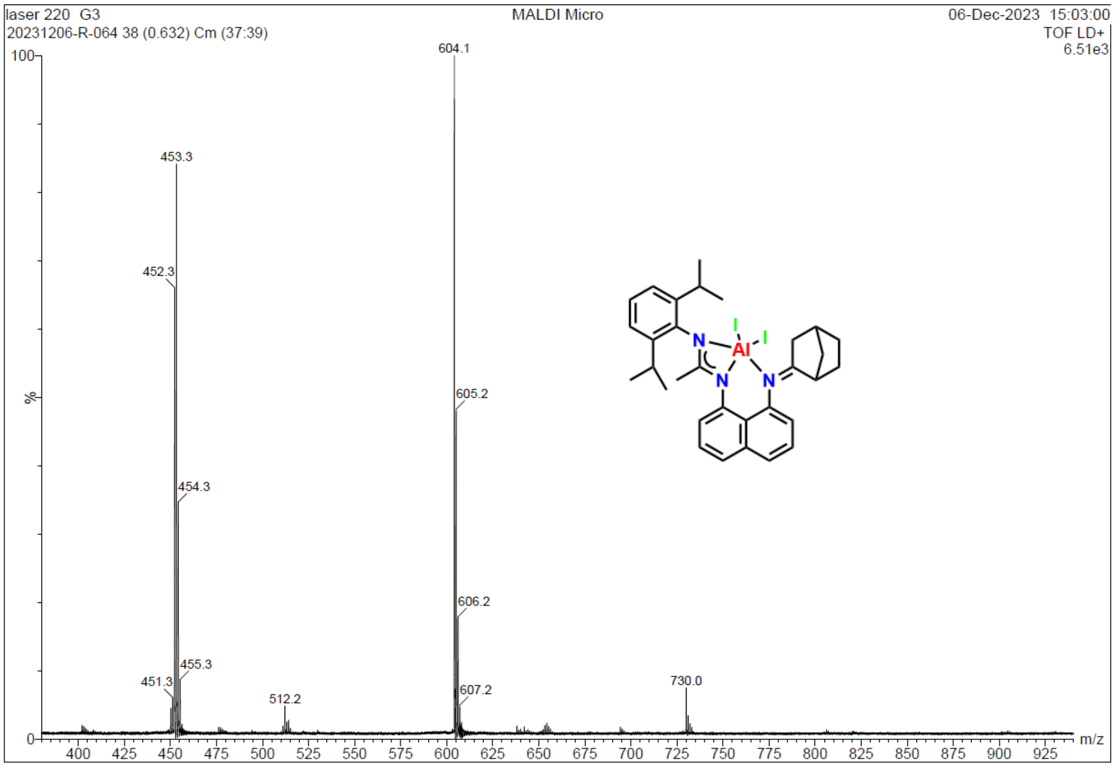

MS (Maldi-TOF) m/z: 730.0 ([M]$^+$), 604.1([M - I]$^+$).

Crystal data and structure refinement of ligand LH (amidine-imine)

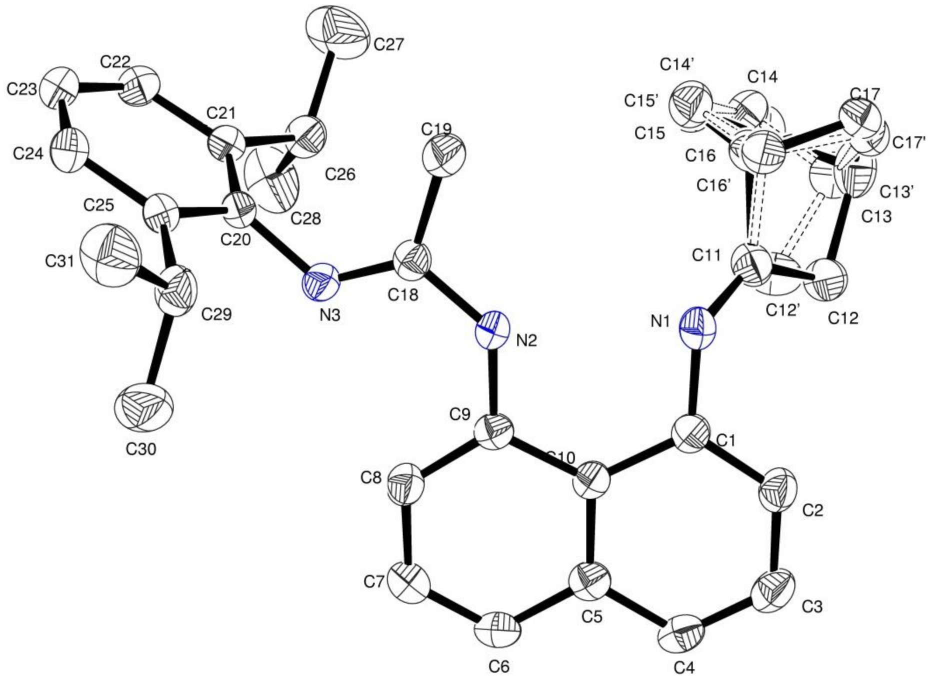

Figure S19 : Asymmetic Unit

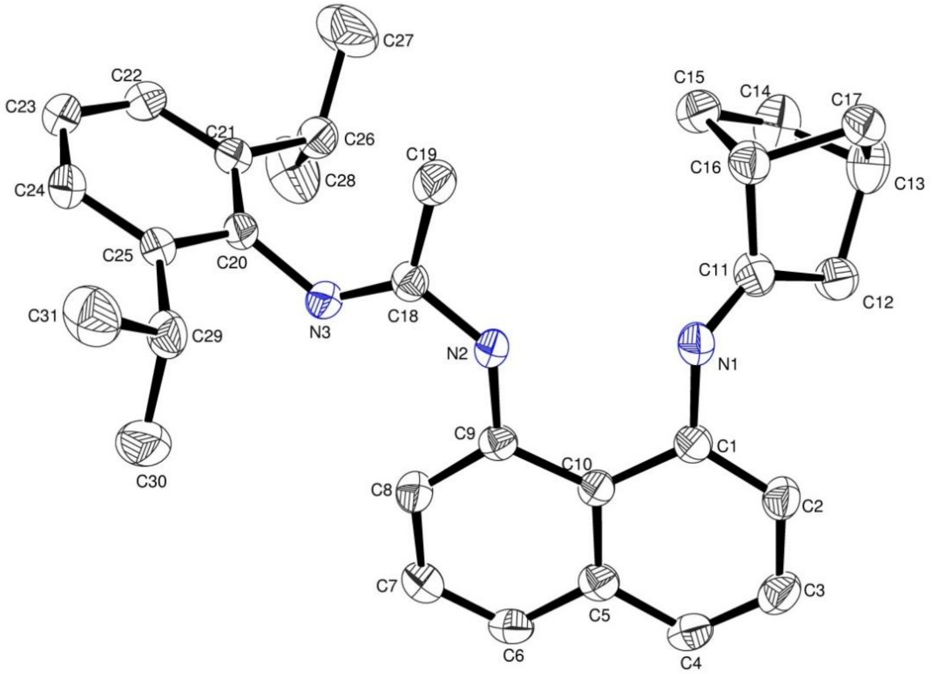

Figure S20 : Compound

Table S1. Crystal data and structure refinement for F210423.

Identification code	F210423
Empirical formula	C31 H37 N3
Formula weight	451.64
Temperature	193(2) K
Wavelength	0.71073 A
Crystal system, space group	Monoclinic, P 21/n
Unit cell dimensions	a = 9.5850(12) A alpha = 90 deg.<br>b = 14.2533(18) A beta = 103.816(4) deg.<br>c = 19.195(3) A gamma = 90 deg.
Volume	2546.5(6) A^3
Z, Calculated density	4, 1.178 Mg/m^3
Absorption coefficient	0.069 mm^-1
F(000)	976
Crystal size	0.200 x 0.080 x 0.040 mm
Theta range for data collection	1.799 to 25.257 deg.
Limiting indices	-11<=h<=11, -17<=k<=17, -23<=l<=22
Reflections collected / unique	46065 / 4615 [R(int) = 0.0874]
Completeness to theta = 25.242	99.9 %
Refinement method	Full-matrix least-squares on F^2
Data / restraints / parameters	4615 / 205 / 372
Goodness-of-fit on F^2	1.031
Final R indices [I>2sigma(I)]	R1 = 0.0549, wR2 = 0.1267
R indices (all data)	R1 = 0.0958, wR2 = 0.1488
Largest diff. peak and hole	0.410 and -0.249 e.A^-3

Table S2. Atomic coordinates (x 10^4) and equivalent isotropic displacement parameters (A^2 x 10^3) for F210423.
U(eq) is defined as one third of the trace of the orthogonalized Uij tensor.

	x	y	z	U(eq)
C(1)	5286(3)	6259(2)	6169(1)	27(1)
C(2)	5645(3)	6627(2)	5574(1)	33(1)
C(3)	4855(3)	6427(2)	4882(1)	36(1)
C(4)	3685(3)	5857(2)	4785(1)	32(1)
C(5)	3282(3)	5438(2)	5378(1)	28(1)
C(6)	2080(3)	4830(2)	5252(1)	33(1)
C(7)	1718(3)	4387(2)	5809(1)	35(1)
C(8)	2524(3)	4518(2)	6511(1)	31(1)
C(9)	3700(2)	5110(2)	6664(1)	24(1)
C(10)	4106(2)	5607(2)	6086(1)	24(1)
N(1)	6129(2)	6463(1)	6868(1)	30(1)
C(11)	6439(3)	7298(2)	7083(1)	33(1)
C(12)	6208(6)	8249(2)	6686(2)	36(1)
C(13)	6854(6)	8954(3)	7335(3)	47(1)
C(14)	5714(7)	8892(4)	7746(3)	53(1)
C(15)	6000(8)	7971(4)	8162(3)	40(1)
C(16)	7183(5)	7513(3)	7851(2)	34(1)
C(17)	8112(4)	8377(3)	7777(3)	33(1)
C(12')	5500(20)	8165(8)	6815(9)	43(3)
C(13')	6430(20)	8955(8)	7318(9)	41(2)
C(14')	5740(20)	8841(16)	7940(11)	40(3)
C(15')	6200(30)	7860(15)	8228(10)	41(3)
C(16')	7330(20)	7565(9)	7812(6)	36(3)
C(17')	7916(17)	8525(12)	7636(12)	38(3)
C(18)	4331(3)	4937(2)	8010(1)	26(1)
C(19)	5555(3)	5183(2)	8633(1)	38(1)
C(20)	2984(2)	4215(2)	8735(1)	26(1)
C(21)	2339(3)	4814(2)	9145(1)	28(1)
C(22)	1954(3)	4455(2)	9745(1)	35(1)
C(23)	2221(3)	3529(2)	9946(1)	37(1)
C(24)	2915(3)	2957(2)	9558(1)	34(1)
C(25)	3312(3)	3278(2)	8951(1)	28(1)
C(26)	2063(3)	5845(2)	8943(1)	35(1)
C(27)	2735(4)	6494(2)	9565(2)	74(1)
C(28)	484(3)	6053(2)	8675(2)	67(1)
C(29)	4048(3)	2647(2)	8512(1)	35(1)
C(30)	2970(4)	2256(2)	7861(2)	50(1)
C(31)	4916(3)	1851(2)	8941(2)	50(1)
N(2)	4553(2)	5222(1)	7362(1)	27(1)
N(3)	3187(2)	4510(1)	8062(1)	28(1)

Table S3. Bond lengths [A] and angles [deg] for F210423.

C(1)-C(2)	1.371(3)
C(1)-N(1)	1.422(3)
C(1)-C(10)	1.444(3)
C(2)-C(3)	1.393(4)
C(2)-H(2)	0.9500
C(3)-C(4)	1.360(4)
C(3)-H(3)	0.9500
C(4)-C(5)	1.419(3)
C(4)-H(4)	0.9500
C(5)-C(6)	1.416(3)
C(5)-C(10)	1.419(3)
C(6)-C(7)	1.357(4)
C(6)-H(6)	0.9500
C(7)-C(8)	1.396(3)
C(7)-H(7)	0.9500
C(8)-C(9)	1.382(3)
C(8)-H(8)	0.9500
C(9)-N(2)	1.403(3)
C(9)-C(10)	1.447(3)
N(1)-C(11)	1.271(3)
C(11)-C(16')	1.503(8)
C(11)-C(16)	1.506(4)
C(11)-C(12')	1.541(8)
C(11)-C(12)	1.545(4)
C(12)-C(13)	1.605(5)
C(12)-H(12A)	0.9900
C(12)-H(12B)	0.9900
C(13)-C(14)	1.496(5)
C(13)-C(17)	1.537(5)
C(13)-H(13)	1.0000
C(14)-C(15)	1.528(5)
C(14)-H(14A)	0.9900
C(14)-H(14B)	0.9900
C(15)-C(16)	1.547(4)
C(15)-H(15A)	0.9900
C(15)-H(15B)	0.9900
C(16)-C(17)	1.546(4)
C(16)-H(16)	1.0000
C(17)-H(17A)	0.9900
C(17)-H(17B)	0.9900
C(12')-C(13')	1.606(9)
C(12')-H(12C)	0.9900
C(12')-H(12D)	0.9900
C(13')-C(14')	1.509(9)
C(13')-C(17')	1.535(9)
C(13')-H(13')	1.0000
C(14')-C(15')	1.531(9)
C(14')-H(14C)	0.9900
C(14')-H(14D)	0.9900
C(15')-C(16')	1.545(8)
C(15')-H(15C)	0.9900
C(15')-H(15D)	0.9900
C(16')-C(17')	1.548(8)
C(16')-H(16')	1.0000

C(17')-H(17C)	0.9900
C(17')-H(17D)	0.9900
C(18)-N(3)	1.278(3)
C(18)-N(2)	1.371(3)
C(18)-C(19)	1.504(3)
C(19)-H(19A)	0.9800
C(19)-H(19B)	0.9800
C(19)-H(19C)	0.9800
C(20)-C(21)	1.402(3)
C(20)-C(25)	1.411(3)
C(20)-N(3)	1.415(3)
C(21)-C(22)	1.389(3)
C(21)-C(26)	1.526(3)
C(22)-C(23)	1.381(4)
C(22)-H(22)	0.9500
C(23)-C(24)	1.380(4)
C(23)-H(23)	0.9500
C(24)-C(25)	1.386(3)
C(24)-H(24)	0.9500
C(25)-C(29)	1.516(3)
C(26)-C(28)	1.507(4)
C(26)-C(27)	1.527(4)
C(26)-H(26)	1.0000
C(27)-H(27A)	0.9800
C(27)-H(27B)	0.9800
C(27)-H(27C)	0.9800
C(28)-H(28A)	0.9800
C(28)-H(28B)	0.9800
C(28)-H(28C)	0.9800
C(29)-C(30)	1.524(4)
C(29)-C(31)	1.527(4)
C(29)-H(29)	1.0000
C(30)-H(30A)	0.9800
C(30)-H(30B)	0.9800
C(30)-H(30C)	0.9800
C(31)-H(31A)	0.9800
C(31)-H(31B)	0.9800
C(31)-H(31C)	0.9800
N(2)-H(102)	0.90(3)

C(2)-C(1)-N(1)	120.6(2)
C(2)-C(1)-C(10)	120.0(2)
N(1)-C(1)-C(10)	119.3(2)
C(1)-C(2)-C(3)	121.9(2)
C(1)-C(2)-H(2)	119.1
C(3)-C(2)-H(2)	119.1
C(4)-C(3)-C(2)	119.7(2)
C(4)-C(3)-H(3)	120.1
C(2)-C(3)-H(3)	120.1
C(3)-C(4)-C(5)	121.0(2)
C(3)-C(4)-H(4)	119.5
C(5)-C(4)-H(4)	119.5
C(6)-C(5)-C(4)	119.1(2)
C(6)-C(5)-C(10)	120.8(2)
C(4)-C(5)-C(10)	120.0(2)
C(7)-C(6)-C(5)	120.0(2)
C(7)-C(6)-H(6)	120.0

C(5)-C(6)-H(6)	120.0
C(6)-C(7)-C(8)	120.8(2)
C(6)-C(7)-H(7)	119.6
C(8)-C(7)-H(7)	119.6
C(9)-C(8)-C(7)	121.5(2)
C(9)-C(8)-H(8)	119.3
C(7)-C(8)-H(8)	119.3
C(8)-C(9)-N(2)	122.2(2)
C(8)-C(9)-C(10)	119.4(2)
N(2)-C(9)-C(10)	118.4(2)
C(5)-C(10)-C(1)	117.2(2)
C(5)-C(10)-C(9)	117.5(2)
C(1)-C(10)-C(9)	125.3(2)
C(11)-N(1)-C(1)	122.3(2)
N(1)-C(11)-C(16')	125.2(6)
N(1)-C(11)-C(16)	121.8(3)
N(1)-C(11)-C(12')	124.7(5)
C(16')-C(11)-C(12')	105.2(7)
N(1)-C(11)-C(12)	131.8(2)
C(16)-C(11)-C(12)	106.4(3)
C(11)-C(12)-C(13)	100.6(3)
C(11)-C(12)-H(12A)	111.7
C(13)-C(12)-H(12A)	111.7
C(11)-C(12)-H(12B)	111.7
C(13)-C(12)-H(12B)	111.7
H(12A)-C(12)-H(12B)	109.4
C(14)-C(13)-C(17)	104.6(3)
C(14)-C(13)-C(12)	100.2(4)
C(17)-C(13)-C(12)	101.5(3)
C(14)-C(13)-H(13)	116.1
C(17)-C(13)-H(13)	116.1
C(12)-C(13)-H(13)	116.1
C(13)-C(14)-C(15)	105.3(3)
C(13)-C(14)-H(14A)	110.7
C(15)-C(14)-H(14A)	110.7
C(13)-C(14)-H(14B)	110.7
C(15)-C(14)-H(14B)	110.7
H(14A)-C(14)-H(14B)	108.8
C(14)-C(15)-C(16)	102.5(3)
C(14)-C(15)-H(15A)	111.3
C(16)-C(15)-H(15A)	111.3
C(14)-C(15)-H(15B)	111.3
C(16)-C(15)-H(15B)	111.3
H(15A)-C(15)-H(15B)	109.2
C(11)-C(16)-C(17)	102.8(3)
C(11)-C(16)-C(15)	104.1(3)
C(17)-C(16)-C(15)	101.0(3)
C(11)-C(16)-H(16)	115.7
C(17)-C(16)-H(16)	115.7
C(15)-C(16)-H(16)	115.7
C(13)-C(17)-C(16)	94.8(3)
C(13)-C(17)-H(17A)	112.8
C(16)-C(17)-H(17A)	112.8
C(13)-C(17)-H(17B)	112.8
C(16)-C(17)-H(17B)	112.8
H(17A)-C(17)-H(17B)	110.2
C(11)-C(12')-C(13')	99.6(6)

C(11)-C(12')-H(12C)	111.8
C(13')-C(12')-H(12C)	111.8
C(11)-C(12')-H(12D)	111.8
C(13')-C(12')-H(12D)	111.8
H(12C)-C(12')-H(12D)	109.6
C(14')-C(13')-C(17')	100.7(9)
C(14')-C(13')-C(12')	96.6(10)
C(17')-C(13')-C(12')	106.6(8)
C(14')-C(13')-H(13')	116.7
C(17')-C(13')-H(13')	116.7
C(12')-C(13')-H(13')	116.7
C(13')-C(14')-C(15')	103.7(7)
C(13')-C(14')-H(14C)	111.0
C(15')-C(14')-H(14C)	111.0
C(13')-C(14')-H(14D)	111.0
C(15')-C(14')-H(14D)	111.0
H(14C)-C(14')-H(14D)	109.0
C(14')-C(15')-C(16')	104.0(7)
C(14')-C(15')-H(15C)	111.0
C(16')-C(15')-H(15C)	111.0
C(14')-C(15')-H(15D)	111.0
C(16')-C(15')-H(15D)	111.0
H(15C)-C(15')-H(15D)	109.0
C(11)-C(16')-C(15')	103.9(10)
C(11)-C(16')-C(17')	100.1(8)
C(15')-C(16')-C(17')	102.0(8)
C(11)-C(16')-H(16')	116.2
C(15')-C(16')-H(16')	116.2
C(17')-C(16')-H(16')	116.2
C(13')-C(17')-C(16')	95.0(7)
C(13')-C(17')-H(17C)	112.7
C(16')-C(17')-H(17C)	112.7
C(13')-C(17')-H(17D)	112.7
C(16')-C(17')-H(17D)	112.7
H(17C)-C(17')-H(17D)	110.2
N(3)-C(18)-N(2)	122.3(2)
N(3)-C(18)-C(19)	124.7(2)
N(2)-C(18)-C(19)	113.0(2)
C(18)-C(19)-H(19A)	109.5
C(18)-C(19)-H(19B)	109.5
H(19A)-C(19)-H(19B)	109.5
C(18)-C(19)-H(19C)	109.5
H(19A)-C(19)-H(19C)	109.5
H(19B)-C(19)-H(19C)	109.5
C(21)-C(20)-C(25)	120.6(2)
C(21)-C(20)-N(3)	120.4(2)
C(25)-C(20)-N(3)	118.7(2)
C(22)-C(21)-C(20)	118.8(2)
C(22)-C(21)-C(26)	120.1(2)
C(20)-C(21)-C(26)	121.1(2)
C(23)-C(22)-C(21)	121.1(2)
C(23)-C(22)-H(22)	119.5
C(21)-C(22)-H(22)	119.5
C(24)-C(23)-C(22)	119.6(2)
C(24)-C(23)-H(23)	120.2
C(22)-C(23)-H(23)	120.2
C(23)-C(24)-C(25)	121.6(2)

C(23)-C(24)-H(24)	119.2
C(25)-C(24)-H(24)	119.2
C(24)-C(25)-C(20)	118.2(2)
C(24)-C(25)-C(29)	121.9(2)
C(20)-C(25)-C(29)	119.9(2)
C(28)-C(26)-C(21)	112.0(2)
C(28)-C(26)-C(27)	110.5(3)
C(21)-C(26)-C(27)	111.6(2)
C(28)-C(26)-H(26)	107.5
C(21)-C(26)-H(26)	107.5
C(27)-C(26)-H(26)	107.5
C(26)-C(27)-H(27A)	109.5
C(26)-C(27)-H(27B)	109.5
H(27A)-C(27)-H(27B)	109.5
C(26)-C(27)-H(27C)	109.5
H(27A)-C(27)-H(27C)	109.5
H(27B)-C(27)-H(27C)	109.5
C(26)-C(28)-H(28A)	109.5
C(26)-C(28)-H(28B)	109.5
H(28A)-C(28)-H(28B)	109.5
C(26)-C(28)-H(28C)	109.5
H(28A)-C(28)-H(28C)	109.5
H(28B)-C(28)-H(28C)	109.5
C(25)-C(29)-C(30)	111.0(2)
C(25)-C(29)-C(31)	114.0(2)
C(30)-C(29)-C(31)	110.3(2)
C(25)-C(29)-H(29)	107.1
C(30)-C(29)-H(29)	107.1
C(31)-C(29)-H(29)	107.1
C(29)-C(30)-H(30A)	109.5
C(29)-C(30)-H(30B)	109.5
H(30A)-C(30)-H(30B)	109.5
C(29)-C(30)-H(30C)	109.5
H(30A)-C(30)-H(30C)	109.5
H(30B)-C(30)-H(30C)	109.5
C(29)-C(31)-H(31A)	109.5
C(29)-C(31)-H(31B)	109.5
H(31A)-C(31)-H(31B)	109.5
C(29)-C(31)-H(31C)	109.5
H(31A)-C(31)-H(31C)	109.5
H(31B)-C(31)-H(31C)	109.5
C(18)-N(2)-C(9)	130.6(2)
C(18)-N(2)-H(102)	114.5(18)
C(9)-N(2)-H(102)	114.8(18)
C(18)-N(3)-C(20)	121.4(2)

Symmetry transformations used to generate equivalent atoms:

Table S4. Anisotropic displacement parameters (A^2 x 10^3) for F210423.
The anisotropic displacement factor exponent takes the form:
-2 pi^2 [h^2 a*^2 U11 + ... + 2 h k a* b* U12]

	U11	U22	U33	U23	U13	U12
C(1)	32(1)	22(1)	28(1)	0(1)	10(1)	2(1)
C(2)	41(2)	27(1)	34(2)	2(1)	16(1)	-3(1)
C(3)	50(2)	31(2)	31(2)	5(1)	17(1)	5(1)
C(4)	42(2)	30(1)	25(1)	0(1)	9(1)	8(1)
C(5)	34(1)	20(1)	30(1)	0(1)	9(1)	6(1)
C(6)	38(2)	32(1)	26(1)	-5(1)	3(1)	1(1)
C(7)	34(2)	33(1)	37(2)	-4(1)	6(1)	-5(1)
C(8)	35(1)	29(1)	31(1)	3(1)	11(1)	-2(1)
C(9)	28(1)	21(1)	25(1)	-1(1)	7(1)	4(1)
C(10)	30(1)	18(1)	27(1)	0(1)	11(1)	5(1)
N(1)	32(1)	28(1)	31(1)	3(1)	9(1)	-5(1)
C(11)	32(1)	31(2)	35(2)	2(1)	5(1)	-2(1)
C(12)	40(3)	32(2)	35(2)	1(1)	9(2)	0(2)
C(13)	38(3)	44(2)	62(2)	13(2)	19(2)	5(2)
C(14)	52(2)	50(2)	62(3)	11(2)	22(3)	4(2)
C(15)	38(3)	50(3)	33(2)	-2(2)	9(2)	-7(2)
C(16)	30(2)	37(2)	31(2)	4(2)	2(2)	-5(2)
C(17)	30(2)	36(2)	32(2)	-1(2)	4(2)	-4(2)
C(12')	44(6)	35(5)	42(5)	-5(4)	-2(5)	15(5)
C(13')	39(4)	38(4)	43(4)	0(4)	9(4)	6(4)
C(14')	40(4)	45(4)	38(5)	0(4)	17(4)	2(4)
C(15')	40(5)	44(4)	38(4)	6(4)	9(4)	-3(4)
C(16')	33(4)	41(4)	32(4)	2(4)	5(4)	-3(4)
C(17')	33(4)	41(4)	39(5)	4(4)	4(4)	-6(4)
C(18)	30(1)	21(1)	28(1)	2(1)	8(1)	2(1)
C(19)	37(2)	46(2)	29(1)	5(1)	5(1)	-7(1)
C(20)	26(1)	26(1)	24(1)	2(1)	4(1)	-6(1)
C(21)	31(1)	27(1)	26(1)	-1(1)	5(1)	-3(1)
C(22)	40(2)	36(2)	29(1)	-3(1)	12(1)	-3(1)
C(23)	45(2)	38(2)	28(1)	2(1)	11(1)	-10(1)
C(24)	42(2)	27(1)	31(1)	5(1)	8(1)	-5(1)
C(25)	33(1)	22(1)	27(1)	0(1)	7(1)	-5(1)
C(26)	43(2)	28(1)	35(2)	1(1)	13(1)	4(1)
C(27)	107(3)	32(2)	70(2)	-6(2)	-3(2)	-4(2)
C(28)	49(2)	47(2)	101(3)	12(2)	8(2)	10(2)
C(29)	47(2)	21(1)	39(2)	3(1)	17(1)	1(1)
C(30)	75(2)	36(2)	40(2)	-5(1)	14(2)	1(2)
C(31)	57(2)	33(2)	59(2)	1(1)	12(2)	8(1)
N(2)	30(1)	28(1)	24(1)	2(1)	8(1)	-5(1)
N(3)	34(1)	25(1)	24(1)	1(1)	8(1)	-1(1)

Crystal data and structure refinement of ligand LH (amidine-aminal)

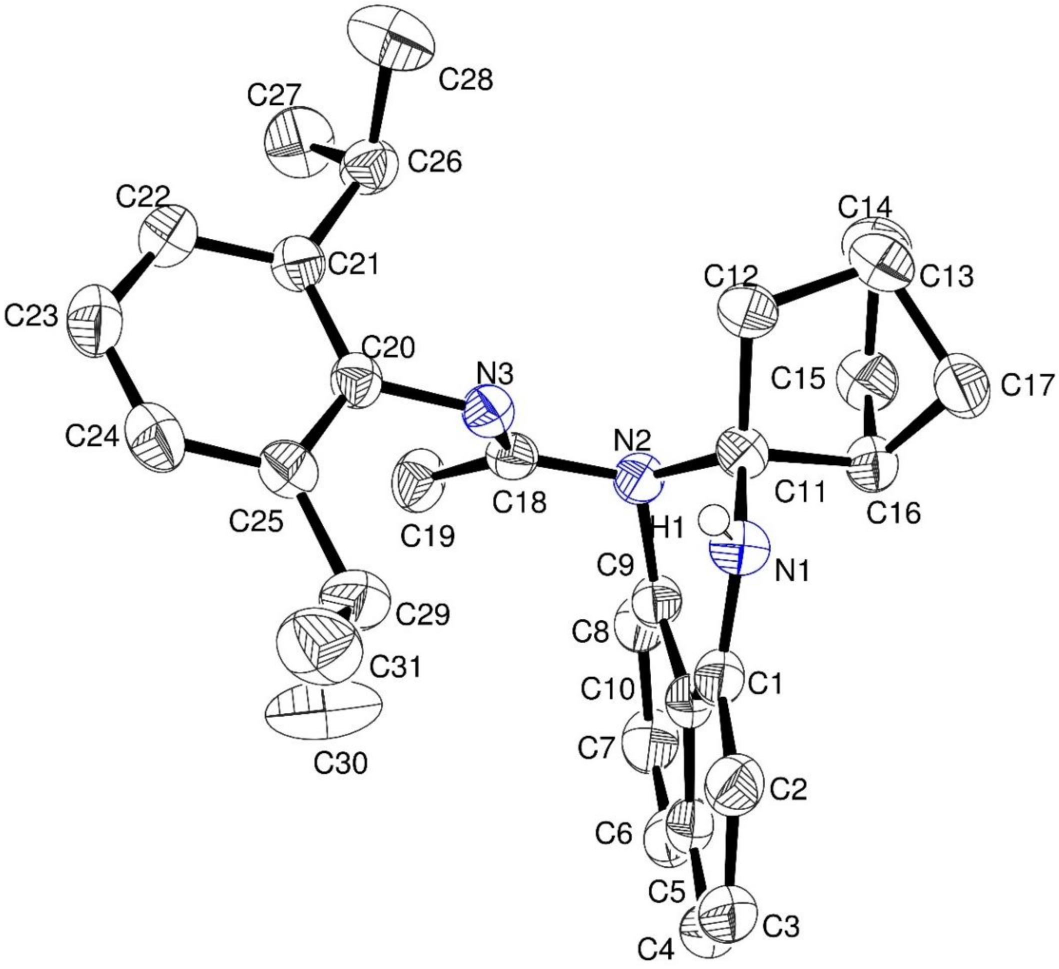

Figure S21 : Asymmetric Unit

Table S5. Crystal data and structure refinement for R-093.

Identification code	R-093
Empirical formula	C31 H37 N3
Formula weight	451.64
Temperature	193(2) K
Wavelength	1.54178 A
Crystal system, space group	Orthorhombic, P 21 21 21
Unit cell dimensions	a = 10.5162(6) A alpha = 90 deg.<br>b = 11.6735(7) A beta = 90 deg.<br>c = 20.6130(12) A gamma = 90 deg.
Volume	2530.5(3) A^3
Z, Calculated density	4, 1.186 Mg/m^3
Absorption coefficient	0.526 mm^-1
F(000)	976
Crystal size	0.200 x 0.060 x 0.040 mm
Theta range for data collection	4.290 to 72.292 deg.
Limiting indices	-12<=h<=12, -14<=k<=14, -25<=l<=25
Reflections collected / unique	23297 / 4992 [R(int) = 0.0666]
Completeness to theta = 67.679	99.9 %
Refinement method	Full-matrix least-squares on F^2
Data / restraints / parameters	4992 / 0 / 316
Goodness-of-fit on F^2	1.055
Final R indices [I>2sigma(I)]	R1 = 0.0482, wR2 = 0.1127
R indices (all data)	R1 = 0.0575, wR2 = 0.1194
Absolute structure parameter	0.2(4)
Extinction coefficient	0.0053(5)
Largest diff. peak and hole	0.245 and -0.161 e.A^-3

Table S6. Atomic coordinates (x 10^4) and equivalent isotropic displacement parameters (A^2 x 10^3) for R-093.
U(eq) is defined as one third of the trace of the orthogonalized Uij tensor.

	x	y	z	U(eq)
C(1)	1892(3)	4297(3)	6168(1)	34(1)
C(2)	694(3)	3879(3)	6302(2)	41(1)
C(3)	-384(3)	4555(3)	6190(2)	48(1)
C(4)	-284(3)	5639(3)	5944(2)	48(1)
C(5)	923(3)	6117(3)	5817(1)	40(1)
C(6)	1100(4)	7233(3)	5566(2)	47(1)
C(7)	2279(4)	7670(3)	5479(2)	47(1)
C(8)	3381(3)	7046(3)	5658(1)	39(1)
C(9)	3244(3)	5968(2)	5911(1)	32(1)
C(10)	2019(3)	5459(3)	5960(1)	34(1)
C(11)	4157(3)	4101(2)	5942(1)	31(1)
C(12)	5311(3)	3319(3)	6089(1)	37(1)
C(13)	5732(3)	2864(3)	5427(2)	44(1)
C(14)	6381(3)	3842(3)	5057(2)	49(1)
C(15)	5280(3)	4664(3)	4907(2)	46(1)
C(16)	4110(3)	4056(3)	5186(1)	36(1)
C(17)	4463(3)	2796(3)	5072(2)	45(1)
C(18)	4538(3)	5497(2)	6829(1)	31(1)
C(19)	4711(3)	6731(3)	7017(2)	43(1)
C(20)	4922(3)	4793(2)	7874(1)	31(1)
C(21)	6171(3)	4964(2)	8089(1)	34(1)
C(22)	6386(3)	5059(3)	8753(2)	41(1)
C(23)	5402(3)	4962(3)	9194(1)	44(1)
C(24)	4185(3)	4745(3)	8979(1)	43(1)
C(25)	3916(3)	4658(3)	8321(1)	36(1)
C(26)	7305(3)	5008(3)	7631(2)	39(1)
C(27)	8069(4)	6111(3)	7705(2)	55(1)
C(28)	8165(4)	3974(3)	7739(2)	61(1)
C(29)	2567(3)	4440(3)	8090(2)	47(1)
C(30)	1852(4)	5549(4)	8003(3)	84(2)
C(31)	1829(4)	3621(4)	8517(2)	61(1)
N(1)	2978(2)	3622(2)	6199(1)	34(1)
N(2)	4303(2)	5305(2)	6158(1)	31(1)
N(3)	4635(2)	4637(2)	7206(1)	31(1)

Table S7. Bond lengths [A] and angles [deg] for R-093.

C(1)-C(2)	1.379(4)
C(1)-N(1)	1.389(4)
C(1)-C(10)	1.428(4)
C(2)-C(3)	1.400(5)
C(2)-H(2)	0.9500
C(3)-C(4)	1.368(5)
C(3)-H(3)	0.9500
C(4)-C(5)	1.411(5)
C(4)-H(4)	0.9500
C(5)-C(6)	1.414(5)
C(5)-C(10)	1.415(4)
C(6)-C(7)	1.353(5)
C(6)-H(6)	0.9500
C(7)-C(8)	1.418(5)
C(7)-H(7)	0.9500
C(8)-C(9)	1.369(4)
C(8)-H(8)	0.9500
C(9)-C(10)	1.423(4)
C(9)-N(2)	1.449(4)
C(11)-N(1)	1.460(4)
C(11)-N(2)	1.482(4)
C(11)-C(12)	1.549(4)
C(11)-C(16)	1.559(4)
C(12)-C(13)	1.531(4)
C(12)-H(12A)	0.9900
C(12)-H(12B)	0.9900
C(13)-C(17)	1.523(5)
C(13)-C(14)	1.533(5)
C(13)-H(13)	1.0000
C(14)-C(15)	1.535(5)
C(14)-H(14A)	0.9900
C(14)-H(14B)	0.9900
C(15)-C(16)	1.532(4)
C(15)-H(15A)	0.9900
C(15)-H(15B)	0.9900
C(16)-C(17)	1.535(4)
C(16)-H(16)	1.0000
C(17)-H(17A)	0.9900
C(17)-H(17B)	0.9900
C(18)-N(3)	1.275(4)
C(18)-N(2)	1.423(3)
C(18)-C(19)	1.503(4)
C(19)-H(19A)	0.9800
C(19)-H(19B)	0.9800
C(19)-H(19C)	0.9800
C(20)-C(21)	1.401(4)
C(20)-C(25)	1.412(4)
C(20)-N(3)	1.420(3)
C(21)-C(22)	1.393(4)
C(21)-C(26)	1.521(4)
C(22)-C(23)	1.381(5)
C(22)-H(22)	0.9500
C(23)-C(24)	1.378(5)
C(23)-H(23)	0.9500

C(24)-C(25)	1.389(4)
C(24)-H(24)	0.9500
C(25)-C(29)	1.518(4)
C(26)-C(28)	1.525(5)
C(26)-C(27)	1.526(5)
C(26)-H(26)	1.0000
C(27)-H(27A)	0.9800
C(27)-H(27B)	0.9800
C(27)-H(27C)	0.9800
C(28)-H(28A)	0.9800
C(28)-H(28B)	0.9800
C(28)-H(28C)	0.9800
C(29)-C(30)	1.508(6)
C(29)-C(31)	1.513(5)
C(29)-H(29)	1.0000
C(30)-H(30A)	0.9800
C(30)-H(30B)	0.9800
C(30)-H(30C)	0.9800
C(31)-H(31A)	0.9800
C(31)-H(31B)	0.9800
C(31)-H(31C)	0.9800
N(1)-H(1)	0.84(4)
C(2)-C(1)-N(1)	122.7(3)
C(2)-C(1)-C(10)	118.8(3)
N(1)-C(1)-C(10)	118.4(3)
C(1)-C(2)-C(3)	120.5(3)
C(1)-C(2)-H(2)	119.7
C(3)-C(2)-H(2)	119.7
C(4)-C(3)-C(2)	121.3(3)
C(4)-C(3)-H(3)	119.3
C(2)-C(3)-H(3)	119.3
C(3)-C(4)-C(5)	120.2(3)
C(3)-C(4)-H(4)	119.9
C(5)-C(4)-H(4)	119.9
C(4)-C(5)-C(6)	123.4(3)
C(4)-C(5)-C(10)	118.6(3)
C(6)-C(5)-C(10)	118.0(3)
C(7)-C(6)-C(5)	121.1(3)
C(7)-C(6)-H(6)	119.4
C(5)-C(6)-H(6)	119.4
C(6)-C(7)-C(8)	121.3(3)
C(6)-C(7)-H(7)	119.3
C(8)-C(7)-H(7)	119.3
C(9)-C(8)-C(7)	119.1(3)
C(9)-C(8)-H(8)	120.5
C(7)-C(8)-H(8)	120.5
C(8)-C(9)-C(10)	120.4(3)
C(8)-C(9)-N(2)	122.9(3)
C(10)-C(9)-N(2)	116.6(2)
C(5)-C(10)-C(9)	119.7(3)
C(5)-C(10)-C(1)	120.1(3)
C(9)-C(10)-C(1)	120.2(3)
N(1)-C(11)-N(2)	110.0(2)
N(1)-C(11)-C(12)	111.6(2)
N(2)-C(11)-C(12)	114.8(2)
N(1)-C(11)-C(16)	108.8(2)

N(2)-C(11)-C(16)	109.6(2)
C(12)-C(11)-C(16)	101.6(2)
C(13)-C(12)-C(11)	104.8(2)
C(13)-C(12)-H(12A)	110.8
C(11)-C(12)-H(12A)	110.8
C(13)-C(12)-H(12B)	110.8
C(11)-C(12)-H(12B)	110.8
H(12A)-C(12)-H(12B)	108.9
C(17)-C(13)-C(12)	101.1(3)
C(17)-C(13)-C(14)	101.0(3)
C(12)-C(13)-C(14)	108.3(3)
C(17)-C(13)-H(13)	114.9
C(12)-C(13)-H(13)	114.9
C(14)-C(13)-H(13)	114.9
C(13)-C(14)-C(15)	103.3(3)
C(13)-C(14)-H(14A)	111.1
C(15)-C(14)-H(14A)	111.1
C(13)-C(14)-H(14B)	111.1
C(15)-C(14)-H(14B)	111.1
H(14A)-C(14)-H(14B)	109.1
C(16)-C(15)-C(14)	103.9(3)
C(16)-C(15)-H(15A)	111.0
C(14)-C(15)-H(15A)	111.0
C(16)-C(15)-H(15B)	111.0
C(14)-C(15)-H(15B)	111.0
H(15A)-C(15)-H(15B)	109.0
C(15)-C(16)-C(17)	101.1(3)
C(15)-C(16)-C(11)	109.5(2)
C(17)-C(16)-C(11)	100.2(2)
C(15)-C(16)-H(16)	114.8
C(17)-C(16)-H(16)	114.8
C(11)-C(16)-H(16)	114.8
C(13)-C(17)-C(16)	95.1(2)
C(13)-C(17)-H(17A)	112.7
C(16)-C(17)-H(17A)	112.7
C(13)-C(17)-H(17B)	112.7
C(16)-C(17)-H(17B)	112.7
H(17A)-C(17)-H(17B)	110.2
N(3)-C(18)-N(2)	118.9(2)
N(3)-C(18)-C(19)	126.0(3)
N(2)-C(18)-C(19)	115.1(2)
C(18)-C(19)-H(19A)	109.5
C(18)-C(19)-H(19B)	109.5
H(19A)-C(19)-H(19B)	109.5
C(18)-C(19)-H(19C)	109.5
H(19A)-C(19)-H(19C)	109.5
H(19B)-C(19)-H(19C)	109.5
C(21)-C(20)-C(25)	120.8(3)
C(21)-C(20)-N(3)	121.6(2)
C(25)-C(20)-N(3)	117.3(3)
C(22)-C(21)-C(20)	118.3(3)
C(22)-C(21)-C(26)	118.7(3)
C(20)-C(21)-C(26)	123.0(2)
C(23)-C(22)-C(21)	121.3(3)
C(23)-C(22)-H(22)	119.4
C(21)-C(22)-H(22)	119.4
C(24)-C(23)-C(22)	120.0(3)

C(24)-C(23)-H(23)	120.0
C(22)-C(23)-H(23)	120.0
C(23)-C(24)-C(25)	121.0(3)
C(23)-C(24)-H(24)	119.5
C(25)-C(24)-H(24)	119.5
C(24)-C(25)-C(20)	118.5(3)
C(24)-C(25)-C(29)	120.6(3)
C(20)-C(25)-C(29)	120.9(3)
C(21)-C(26)-C(28)	110.3(3)
C(21)-C(26)-C(27)	112.3(3)
C(28)-C(26)-C(27)	110.0(3)
C(21)-C(26)-H(26)	108.0
C(28)-C(26)-H(26)	108.0
C(27)-C(26)-H(26)	108.0
C(26)-C(27)-H(27A)	109.5
C(26)-C(27)-H(27B)	109.5
H(27A)-C(27)-H(27B)	109.5
C(26)-C(27)-H(27C)	109.5
H(27A)-C(27)-H(27C)	109.5
H(27B)-C(27)-H(27C)	109.5
C(26)-C(28)-H(28A)	109.5
C(26)-C(28)-H(28B)	109.5
H(28A)-C(28)-H(28B)	109.5
C(26)-C(28)-H(28C)	109.5
H(28A)-C(28)-H(28C)	109.5
H(28B)-C(28)-H(28C)	109.5
C(30)-C(29)-C(31)	110.9(3)
C(30)-C(29)-C(25)	111.1(3)
C(31)-C(29)-C(25)	113.8(3)
C(30)-C(29)-H(29)	106.9
C(31)-C(29)-H(29)	106.9
C(25)-C(29)-H(29)	106.9
C(29)-C(30)-H(30A)	109.5
C(29)-C(30)-H(30B)	109.5
H(30A)-C(30)-H(30B)	109.5
C(29)-C(30)-H(30C)	109.5
H(30A)-C(30)-H(30C)	109.5
H(30B)-C(30)-H(30C)	109.5
C(29)-C(31)-H(31A)	109.5
C(29)-C(31)-H(31B)	109.5
H(31A)-C(31)-H(31B)	109.5
C(29)-C(31)-H(31C)	109.5
H(31A)-C(31)-H(31C)	109.5
H(31B)-C(31)-H(31C)	109.5
C(1)-N(1)-C(11)	117.7(2)
C(1)-N(1)-H(1)	120(2)
C(11)-N(1)-H(1)	116(2)
C(18)-N(2)-C(9)	113.0(2)
C(18)-N(2)-C(11)	117.4(2)
C(9)-N(2)-C(11)	108.7(2)
C(18)-N(3)-C(20)	120.5(2)

Symmetry transformations used to generate equivalent atoms:

Table S8. Anisotropic displacement parameters (A^2 x 10^3) for R-093.
The anisotropic displacement factor exponent takes the form:
-2 pi^2 [h^2 a*^2 U11 + ... + 2 h k a* b* U12]

	U11	U22	U33	U23	U13	U12
C(1)	35(2)	38(2)	29(1)	-4(1)	-1(1)	-1(1)
C(2)	38(2)	49(2)	37(2)	-3(1)	1(1)	-5(2)
C(3)	33(2)	68(2)	42(2)	-13(2)	1(1)	-2(2)
C(4)	38(2)	65(2)	41(2)	-13(2)	-5(1)	13(2)
C(5)	42(2)	46(2)	31(1)	-9(1)	-6(1)	7(1)
C(6)	55(2)	45(2)	41(2)	-5(1)	-14(2)	18(2)
C(7)	64(2)	32(2)	44(2)	3(1)	-11(2)	10(2)
C(8)	47(2)	33(2)	38(2)	3(1)	-4(1)	0(1)
C(9)	36(2)	30(1)	30(1)	0(1)	-2(1)	2(1)
C(10)	37(2)	38(2)	28(1)	-5(1)	-2(1)	4(1)
C(11)	33(2)	27(1)	33(1)	1(1)	2(1)	0(1)
C(12)	37(2)	33(2)	41(2)	2(1)	1(1)	4(1)
C(13)	43(2)	39(2)	49(2)	0(1)	7(2)	9(1)
C(14)	43(2)	55(2)	50(2)	7(2)	9(2)	1(2)
C(15)	50(2)	46(2)	41(2)	5(1)	4(1)	3(2)
C(16)	39(2)	37(2)	33(1)	-2(1)	0(1)	0(1)
C(17)	48(2)	44(2)	45(2)	-12(2)	5(2)	1(2)
C(18)	28(1)	30(1)	34(1)	-1(1)	-1(1)	3(1)
C(19)	55(2)	30(2)	43(2)	-5(1)	-10(2)	2(1)
C(20)	36(2)	27(1)	31(1)	-2(1)	-1(1)	1(1)
C(21)	35(2)	33(2)	34(1)	-2(1)	0(1)	0(1)
C(22)	42(2)	45(2)	37(2)	0(1)	-5(1)	-1(1)
C(23)	58(2)	43(2)	31(1)	-2(1)	-2(1)	2(2)
C(24)	52(2)	42(2)	34(2)	-1(1)	9(1)	4(2)
C(25)	36(2)	33(2)	39(2)	-2(1)	4(1)	2(1)
C(26)	32(2)	45(2)	39(2)	1(1)	-1(1)	-3(1)
C(27)	53(2)	53(2)	60(2)	1(2)	5(2)	-12(2)
C(28)	52(2)	56(2)	76(3)	11(2)	19(2)	12(2)
C(29)	35(2)	57(2)	50(2)	0(2)	4(1)	-2(2)
C(30)	39(2)	80(3)	134(4)	30(3)	-7(3)	7(2)
C(31)	47(2)	65(2)	71(2)	-2(2)	14(2)	-12(2)
N(1)	33(1)	31(1)	38(1)	4(1)	1(1)	-1(1)
N(2)	33(1)	28(1)	32(1)	0(1)	-2(1)	1(1)
N(3)	31(1)	31(1)	30(1)	-1(1)	1(1)	0(1)

Crystal data and structure refinement of complex $AlMe_2L$

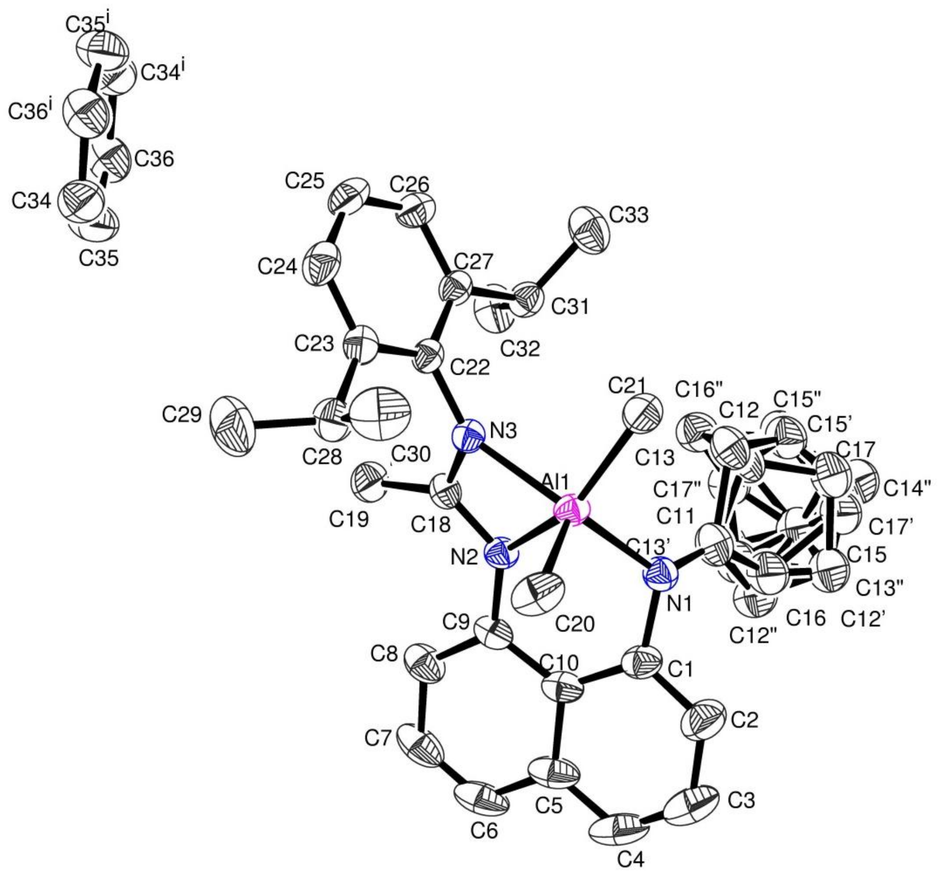

Figure S22 : Asymmetric Unit

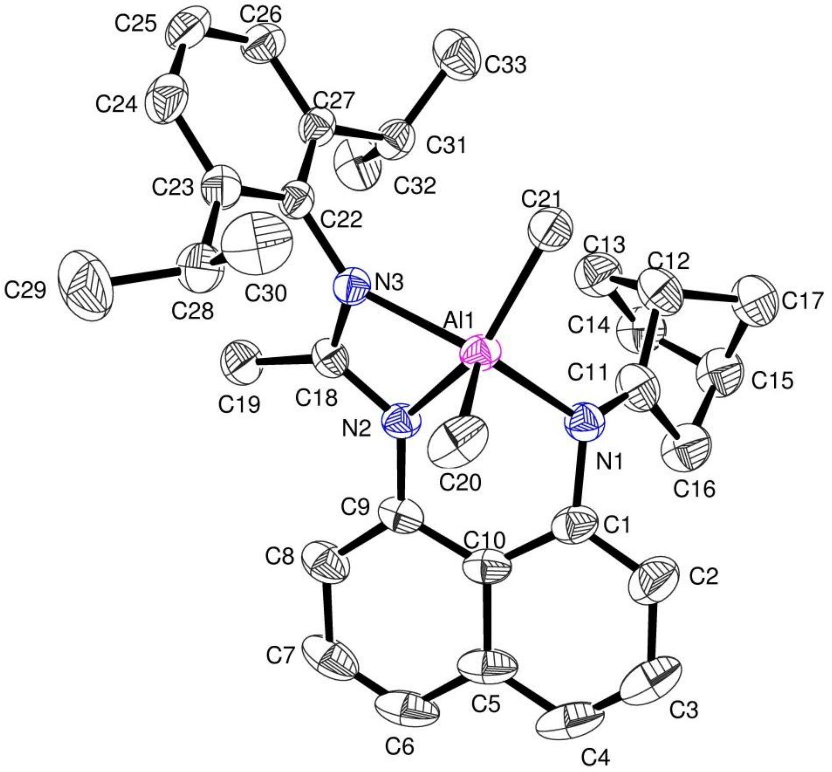
C25
C26
C33
C24
C27
C31
C23
C32
C21
C22
C13
C12
C29
N3
C17
C28
C30
Al1
C14
C11
C15
C19
C18
N2
N1
C20
C16
C9
C1
C8
C10
C2
C7
C5
C3
C6
C4

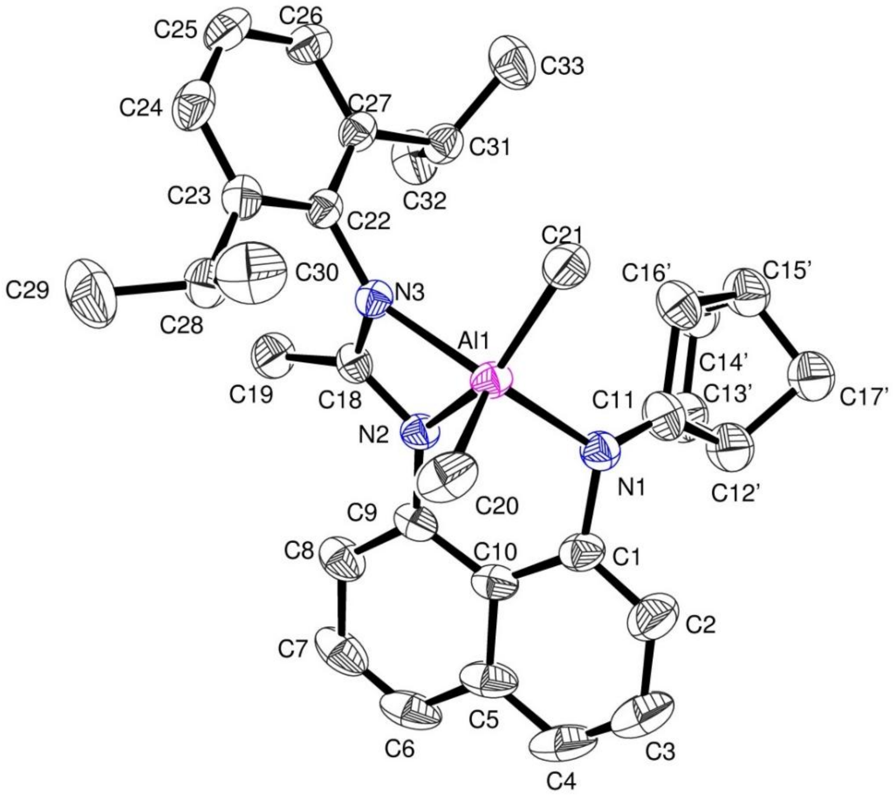
C25
C26
C33
C24
C27
C31
C23
C32
C22
C21
C16'
C15'
C29
C30
N3
C28
C14'
Al1
C13'
C19
C11
C17'
C18
N2
N1
C12'
C20
C9
C8
C10
C1
C2
C7
C5
C3
C6
C4

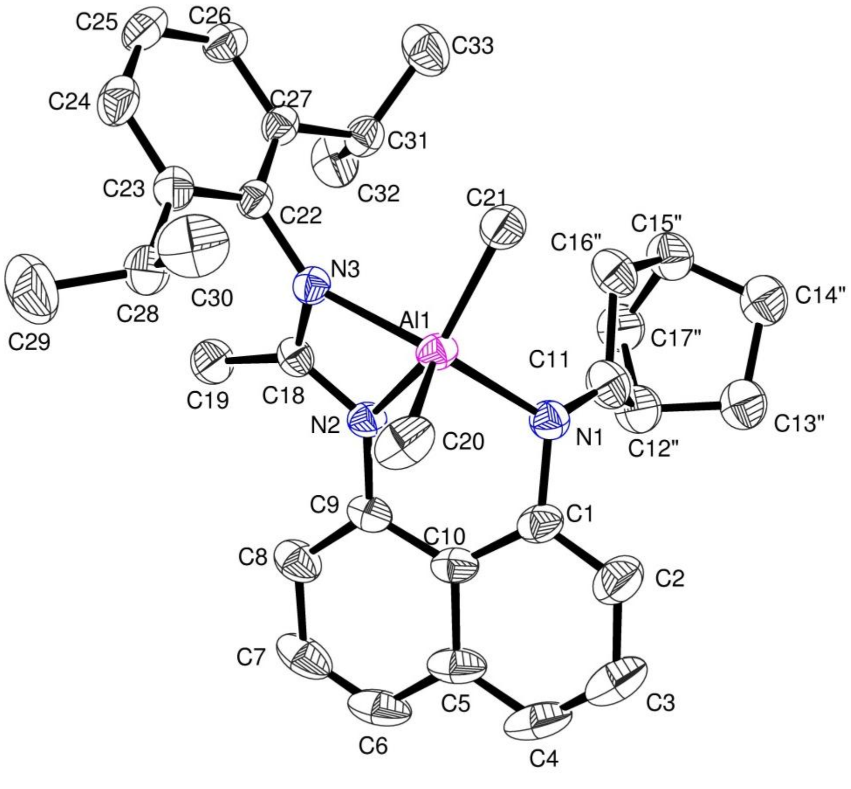

Figure S23 : Compound

Table S9. Crystal data and structure refinement for R-008.

Identification code	R-008
Empirical formula	C33 H42 Al N3, 0.5(C6 H6)
Formula weight	546.73
Temperature	193(2) K
Wavelength	0.71073 A
Crystal system, space group	Monoclinic, P 21/c
Unit cell dimensions	a = 11.2287(6) A alpha = 90 deg.<br>b = 29.4432(17) A beta = 112.423(2) deg.<br>c = 10.3178(6) A gamma = 90 deg.
Volume	3153.3(3) A^3
Z, Calculated density	4, 1.152 Mg/m^3
Absorption coefficient	0.093 mm^-1
F(000)	1180
Crystal size	0.180 x 0.100 x 0.080 mm
Theta range for data collection	2.856 to 26.461 deg.
Limiting indices	-12<=h<=14, -36<=k<=36, -12<=l<=12
Reflections collected / unique	74617 / 6439 [R(int) = 0.0616]
Completeness to theta = 25.242	99.6 %
Refinement method	Full-matrix least-squares on F^2
Data / restraints / parameters	6439 / 525 / 480
Goodness-of-fit on F^2	1.034
Final R indices [I>2sigma(I)]	R1 = 0.0444, wR2 = 0.1072
R indices (all data)	R1 = 0.0644, wR2 = 0.1184
Largest diff. peak and hole	0.253 and -0.292 e.A^-3

Table S10. Atomic coordinates (x 10^4) and equivalent isotropic displacement parameters (A^2 x 10^3) for R-008.
U(eq) is defined as one third of the trace of the orthogonalized Uij tensor.

	x	y	z	U(eq)
Al(1)	5761(1)	3256(1)	7214(1)	29(1)
C(1)	8491(2)	3035(1)	8951(2)	37(1)
C(2)	9115(2)	2656(1)	9667(2)	53(1)
C(3)	10303(2)	2521(1)	9634(3)	67(1)
C(4)	10858(2)	2770(1)	8916(3)	63(1)
C(5)	10230(2)	3153(1)	8119(2)	47(1)
C(6)	10810(2)	3406(1)	7358(2)	54(1)
C(7)	10183(2)	3761(1)	6560(2)	53(1)
C(8)	8927(2)	3880(1)	6435(2)	44(1)
C(9)	8302(1)	3645(1)	7154(2)	33(1)
C(10)	8985(1)	3284(1)	8076(2)	35(1)
C(18)	6111(1)	3979(1)	6126(2)	29(1)
C(19)	6297(2)	4394(1)	5400(2)	45(1)
C(20)	5948(2)	2763(1)	6019(2)	51(1)
C(21)	4291(2)	3145(1)	7793(2)	45(1)
C(22)	3801(1)	4064(1)	5290(2)	28(1)
C(23)	3074(2)	3975(1)	3869(2)	34(1)
C(24)	1934(2)	4217(1)	3212(2)	43(1)
C(25)	1517(2)	4535(1)	3931(2)	43(1)
C(26)	2220(2)	4610(1)	5334(2)	38(1)
C(27)	3364(1)	4376(1)	6040(2)	31(1)
C(28)	3489(2)	3613(1)	3083(2)	44(1)
C(29)	3286(3)	3749(1)	1591(2)	82(1)
C(30)	2813(2)	3169(1)	3111(3)	66(1)
C(31)	4123(2)	4457(1)	7590(2)	34(1)
C(32)	4895(2)	4897(1)	7855(2)	52(1)
C(33)	3270(2)	4451(1)	8433(2)	53(1)
C(34)	368(2)	4627(1)	-552(2)	49(1)
C(35)	1259(2)	4949(1)	160(2)	56(1)
C(36)	895(2)	5324(1)	710(2)	49(1)
N(1)	7333(1)	3182(1)	9126(1)	33(1)
N(2)	7025(1)	3706(1)	7001(1)	30(1)
N(3)	4971(1)	3823(1)	5997(1)	28(1)
C(11)	7482(2)	3328(1)	10344(2)	44(1)
C(12)	6548(3)	3515(1)	10876(3)	46(1)
C(13)	6766(3)	4024(1)	10859(3)	49(1)
C(14)	8076(4)	4098(1)	12094(4)	52(1)
C(15)	8459(3)	3636(1)	12667(3)	52(1)
C(16)	8841(3)	3367(1)	11603(3)	52(1)
C(17)	7169(3)	3411(1)	12479(3)	52(1)
C(12')	8508(6)	3376(3)	11735(5)	51(1)
C(13')	8801(8)	3881(3)	11819(9)	54(1)
C(14')	7616(10)	4099(2)	11998(10)	52(1)
C(15')	6794(8)	3705(3)	12000(9)	49(1)
C(16')	6217(5)	3516(4)	10495(9)	47(1)
C(17')	7778(10)	3334(3)	12748(4)	49(1)
C(12")	8517(14)	3575(5)	11473(14)	50(1)
C(13")	8575(12)	3239(4)	12655(13)	50(1)

C(14")	7450(14)	3396(5)	13054(14)	51(1)
C(15")	6762(13)	3755(5)	11976(13)	49(1)
C(16")	6239(9)	3521(8)	10534(17)	46(1)
C(17")	7925(17)	4006(5)	11810(20)	51(1)

Table S11. Bond lengths [A] and angles [deg] for R-008.

Al(1)-C(20)	1.9667(19)
Al(1)-C(21)	1.9840(17)
Al(1)-N(2)	2.0146(13)
Al(1)-N(3)	2.0739(13)
Al(1)-N(1)	2.0972(14)
Al(1)-C(18)	2.5054(16)
C(1)-C(2)	1.373(3)
C(1)-C(10)	1.429(2)
C(1)-N(1)	1.447(2)
C(2)-C(3)	1.405(3)
C(2)-H(2)	0.9500
C(3)-C(4)	1.352(3)
C(3)-H(3)	0.9500
C(4)-C(5)	1.416(3)
C(4)-H(4)	0.9500
C(5)-C(6)	1.409(3)
C(5)-C(10)	1.434(2)
C(6)-C(7)	1.351(3)
C(6)-H(6)	0.9500
C(7)-C(8)	1.410(3)
C(7)-H(7)	0.9500
C(8)-C(9)	1.385(2)
C(8)-H(8)	0.9500
C(9)-N(2)	1.3935(19)
C(9)-C(10)	1.437(2)
C(18)-N(3)	1.3166(19)
C(18)-N(2)	1.343(2)
C(18)-C(19)	1.491(2)
C(19)-H(19A)	0.9800
C(19)-H(19B)	0.9800
C(19)-H(19C)	0.9800
C(20)-H(20A)	0.9800
C(20)-H(20B)	0.9800
C(20)-H(20C)	0.9800
C(21)-H(21A)	0.9800
C(21)-H(21B)	0.9800
C(21)-H(21C)	0.9800
C(22)-C(27)	1.404(2)
C(22)-C(23)	1.405(2)
C(22)-N(3)	1.4246(19)
C(23)-C(24)	1.394(2)
C(23)-C(28)	1.517(2)
C(24)-C(25)	1.382(3)
C(24)-H(24)	0.9500
C(25)-C(26)	1.377(2)
C(25)-H(25)	0.9500
C(26)-C(27)	1.394(2)
C(26)-H(26)	0.9500
C(27)-C(31)	1.518(2)
C(28)-C(30)	1.518(3)
C(28)-C(29)	1.522(3)
C(28)-H(28)	1.0000
C(29)-H(29A)	0.9800
C(29)-H(29B)	0.9800

C(29)-H(29C)	0.9800
C(30)-H(30A)	0.9800
C(30)-H(30B)	0.9800
C(30)-H(30C)	0.9800
C(31)-C(33)	1.520(2)
C(31)-C(32)	1.523(2)
C(31)-H(31)	1.0000
C(32)-H(32A)	0.9800
C(32)-H(32B)	0.9800
C(32)-H(32C)	0.9800
C(33)-H(33A)	0.9800
C(33)-H(33B)	0.9800
C(33)-H(33C)	0.9800
C(34)-C(36)#1	1.372(3)
C(34)-C(35)	1.372(3)
C(34)-H(34)	0.9500
C(35)-C(36)	1.371(3)
C(35)-H(35)	0.9500
C(36)-H(36)	0.9500
N(1)-C(11)	1.278(2)
C(11)-C(12')	1.464(4)
C(11)-C(12)	1.464(4)
C(11)-C(12")	1.484(15)
C(11)-C(16)	1.586(3)
C(11)-C(16')	1.587(4)
C(11)-C(16")	1.587(4)
C(12)-C(13)	1.519(5)
C(12)-C(17)	1.560(4)
C(12)-H(12)	1.0000
C(13)-C(14)	1.551(4)
C(13)-H(13A)	0.9900
C(13)-H(13B)	0.9900
C(14)-C(15)	1.482(5)
C(14)-H(14A)	0.9900
C(14)-H(14B)	0.9900
C(15)-C(17)	1.536(4)
C(15)-C(16)	1.541(4)
C(15)-H(15)	1.0000
C(16)-H(16A)	0.9900
C(16)-H(16B)	0.9900
C(17)-H(17A)	0.9900
C(17)-H(17B)	0.9900
C(12')-C(13')	1.520(5)
C(12')-C(17')	1.560(4)
C(12')-H(12')	1.0000
C(13')-C(14')	1.551(4)
C(13')-H(13C)	0.9900
C(13')-H(13D)	0.9900
C(14')-C(15')	1.482(5)
C(14')-H(14C)	0.9900
C(14')-H(14D)	0.9900
C(15')-C(17')	1.536(4)
C(15')-C(16')	1.541(4)
C(15')-H(15')	1.0000
C(16')-H(16C)	0.9900
C(16')-H(16D)	0.9900
C(17')-H(17C)	0.9900

C(17')-H(17D)	0.9900
C(12")-C(17")	1.535(16)
C(12")-C(13")	1.553(15)
C(12")-H(12")	1.0000
C(13")-C(14")	1.540(15)
C(13")-H(13E)	0.9900
C(13")-H(13F)	0.9900
C(14")-C(15")	1.517(15)
C(14")-H(14E)	0.9900
C(14")-H(14F)	0.9900
C(15")-C(16")	1.539(14)
C(15")-C(17")	1.564(16)
C(15")-H(15")	1.0000
C(16")-H(16E)	0.9900
C(16")-H(16F)	0.9900
C(17")-H(17E)	0.9900
C(17")-H(17F)	0.9900
C(20)-Al(1)-C(21)	111.60(9)
C(20)-Al(1)-N(2)	99.72(8)
C(21)-Al(1)-N(2)	147.35(7)
C(20)-Al(1)-N(3)	109.73(7)
C(21)-Al(1)-N(3)	95.85(6)
N(2)-Al(1)-N(3)	63.92(5)
C(20)-Al(1)-N(1)	105.12(7)
C(21)-Al(1)-N(1)	101.43(7)
N(2)-Al(1)-N(1)	78.16(5)
N(3)-Al(1)-N(1)	131.63(5)
C(20)-Al(1)-C(18)	105.84(8)
C(21)-Al(1)-C(18)	124.37(7)
N(2)-Al(1)-C(18)	32.32(5)
N(3)-Al(1)-C(18)	31.67(5)
N(1)-Al(1)-C(18)	106.81(5)
C(2)-C(1)-C(10)	121.07(16)
C(2)-C(1)-N(1)	118.40(17)
C(10)-C(1)-N(1)	120.51(15)
C(1)-C(2)-C(3)	120.8(2)
C(1)-C(2)-H(2)	119.6
C(3)-C(2)-H(2)	119.6
C(4)-C(3)-C(2)	119.9(2)
C(4)-C(3)-H(3)	120.0
C(2)-C(3)-H(3)	120.0
C(3)-C(4)-C(5)	121.30(18)
C(3)-C(4)-H(4)	119.4
C(5)-C(4)-H(4)	119.4
C(6)-C(5)-C(4)	120.67(18)
C(6)-C(5)-C(10)	119.60(18)
C(4)-C(5)-C(10)	119.72(19)
C(7)-C(6)-C(5)	120.23(17)
C(7)-C(6)-H(6)	119.9
C(5)-C(6)-H(6)	119.9
C(6)-C(7)-C(8)	121.26(19)
C(6)-C(7)-H(7)	119.4
C(8)-C(7)-H(7)	119.4
C(9)-C(8)-C(7)	121.32(19)
C(9)-C(8)-H(8)	119.3
C(7)-C(8)-H(8)	119.3

C(8)-C(9)-N(2)	125.81(16)
C(8)-C(9)-C(10)	118.21(15)
N(2)-C(9)-C(10)	115.85(14)
C(1)-C(10)-C(5)	116.77(16)
C(1)-C(10)-C(9)	124.12(14)
C(5)-C(10)-C(9)	119.09(16)
N(3)-C(18)-N(2)	108.92(13)
N(3)-C(18)-C(19)	123.48(14)
N(2)-C(18)-C(19)	127.59(14)
N(3)-C(18)-Al(1)	55.79(8)
N(2)-C(18)-Al(1)	53.30(7)
C(19)-C(18)-Al(1)	176.66(12)
C(18)-C(19)-H(19A)	109.5
C(18)-C(19)-H(19B)	109.5
H(19A)-C(19)-H(19B)	109.5
C(18)-C(19)-H(19C)	109.5
H(19A)-C(19)-H(19C)	109.5
H(19B)-C(19)-H(19C)	109.5
Al(1)-C(20)-H(20A)	109.5
Al(1)-C(20)-H(20B)	109.5
H(20A)-C(20)-H(20B)	109.5
Al(1)-C(20)-H(20C)	109.5
H(20A)-C(20)-H(20C)	109.5
H(20B)-C(20)-H(20C)	109.5
Al(1)-C(21)-H(21A)	109.5
Al(1)-C(21)-H(21B)	109.5
H(21A)-C(21)-H(21B)	109.5
Al(1)-C(21)-H(21C)	109.5
H(21A)-C(21)-H(21C)	109.5
H(21B)-C(21)-H(21C)	109.5
C(27)-C(22)-C(23)	120.68(14)
C(27)-C(22)-N(3)	119.43(13)
C(23)-C(22)-N(3)	119.84(14)
C(24)-C(23)-C(22)	118.22(15)
C(24)-C(23)-C(28)	120.83(15)
C(22)-C(23)-C(28)	120.91(14)
C(25)-C(24)-C(23)	121.42(16)
C(25)-C(24)-H(24)	119.3
C(23)-C(24)-H(24)	119.3
C(26)-C(25)-C(24)	119.84(15)
C(26)-C(25)-H(25)	120.1
C(24)-C(25)-H(25)	120.1
C(25)-C(26)-C(27)	120.94(16)
C(25)-C(26)-H(26)	119.5
C(27)-C(26)-H(26)	119.5
C(26)-C(27)-C(22)	118.85(15)
C(26)-C(27)-C(31)	120.51(15)
C(22)-C(27)-C(31)	120.64(13)
C(23)-C(28)-C(30)	109.72(16)
C(23)-C(28)-C(29)	113.41(17)
C(30)-C(28)-C(29)	111.30(19)
C(23)-C(28)-H(28)	107.4
C(30)-C(28)-H(28)	107.4
C(29)-C(28)-H(28)	107.4
C(28)-C(29)-H(29A)	109.5
C(28)-C(29)-H(29B)	109.5
H(29A)-C(29)-H(29B)	109.5

C(28)-C(29)-H(29C)	109.5
H(29A)-C(29)-H(29C)	109.5
H(29B)-C(29)-H(29C)	109.5
C(28)-C(30)-H(30A)	109.5
C(28)-C(30)-H(30B)	109.5
H(30A)-C(30)-H(30B)	109.5
C(28)-C(30)-H(30C)	109.5
H(30A)-C(30)-H(30C)	109.5
H(30B)-C(30)-H(30C)	109.5
C(27)-C(31)-C(33)	112.23(14)
C(27)-C(31)-C(32)	112.04(14)
C(33)-C(31)-C(32)	110.28(15)
C(27)-C(31)-H(31)	107.3
C(33)-C(31)-H(31)	107.3
C(32)-C(31)-H(31)	107.3
C(31)-C(32)-H(32A)	109.5
C(31)-C(32)-H(32B)	109.5
H(32A)-C(32)-H(32B)	109.5
C(31)-C(32)-H(32C)	109.5
H(32A)-C(32)-H(32C)	109.5
H(32B)-C(32)-H(32C)	109.5
C(31)-C(33)-H(33A)	109.5
C(31)-C(33)-H(33B)	109.5
H(33A)-C(33)-H(33B)	109.5
C(31)-C(33)-H(33C)	109.5
H(33A)-C(33)-H(33C)	109.5
H(33B)-C(33)-H(33C)	109.5
C(36)#1-C(34)-C(35)	119.97(18)
C(36)#1-C(34)-H(34)	120.0
C(35)-C(34)-H(34)	120.0
C(36)-C(35)-C(34)	120.44(18)
C(36)-C(35)-H(35)	119.8
C(34)-C(35)-H(35)	119.8
C(35)-C(36)-C(34)#1	119.60(18)
C(35)-C(36)-H(36)	120.2
C(34)#1-C(36)-H(36)	120.2
C(11)-N(1)-C(1)	115.91(14)
C(11)-N(1)-Al(1)	129.68(12)
C(1)-N(1)-Al(1)	112.83(10)
C(18)-N(2)-C(9)	129.98(14)
C(18)-N(2)-Al(1)	94.38(9)
C(9)-N(2)-Al(1)	129.82(11)
C(18)-N(3)-C(22)	123.33(13)
C(18)-N(3)-Al(1)	92.54(9)
C(22)-N(3)-Al(1)	143.93(10)
N(1)-C(11)-C(12')	139.1(3)
N(1)-C(11)-C(12)	130.63(18)
N(1)-C(11)-C(12")	135.0(5)
N(1)-C(11)-C(16)	123.64(18)
C(12)-C(11)-C(16)	105.62(19)
N(1)-C(11)-C(16')	115.3(3)
C(12')-C(11)-C(16')	105.7(2)
N(1)-C(11)-C(16")	116.7(5)
C(12")-C(11)-C(16")	101.0(7)
C(11)-C(12)-C(13)	103.0(3)
C(11)-C(12)-C(17)	103.2(2)
C(13)-C(12)-C(17)	101.4(3)

C(11)-C(12)-H(12)	115.7
C(13)-C(12)-H(12)	115.7
C(17)-C(12)-H(12)	115.7
C(12)-C(13)-C(14)	103.6(2)
C(12)-C(13)-H(13A)	111.0
C(14)-C(13)-H(13A)	111.0
C(12)-C(13)-H(13B)	111.0
C(14)-C(13)-H(13B)	111.0
H(13A)-C(13)-H(13B)	109.0
C(15)-C(14)-C(13)	103.8(2)
C(15)-C(14)-H(14A)	111.0
C(13)-C(14)-H(14A)	111.0
C(15)-C(14)-H(14B)	111.0
C(13)-C(14)-H(14B)	111.0
H(14A)-C(14)-H(14B)	109.0
C(14)-C(15)-C(17)	103.2(3)
C(14)-C(15)-C(16)	107.8(3)
C(17)-C(15)-C(16)	101.2(2)
C(14)-C(15)-H(15)	114.4
C(17)-C(15)-H(15)	114.4
C(16)-C(15)-H(15)	114.4
C(15)-C(16)-C(11)	100.2(2)
C(15)-C(16)-H(16A)	111.7
C(11)-C(16)-H(16A)	111.7
C(15)-C(16)-H(16B)	111.7
C(11)-C(16)-H(16B)	111.7
H(16A)-C(16)-H(16B)	109.5
C(15)-C(17)-C(12)	93.9(2)
C(15)-C(17)-H(17A)	112.9
C(12)-C(17)-H(17A)	112.9
C(15)-C(17)-H(17B)	112.9
C(12)-C(17)-H(17B)	112.9
H(17A)-C(17)-H(17B)	110.4
C(11)-C(12')-C(13')	102.8(3)
C(11)-C(12')-C(17')	103.4(3)
C(13')-C(12')-C(17')	101.3(3)
C(11)-C(12')-H(12')	115.8
C(13')-C(12')-H(12')	115.8
C(17')-C(12')-H(12')	115.8
C(12')-C(13')-C(14')	103.6(3)
C(12')-C(13')-H(13C)	111.0
C(14')-C(13')-H(13C)	111.0
C(12')-C(13')-H(13D)	111.0
C(14')-C(13')-H(13D)	111.0
H(13C)-C(13')-H(13D)	109.0
C(15')-C(14')-C(13')	103.9(3)
C(15')-C(14')-H(14C)	111.0
C(13')-C(14')-H(14C)	111.0
C(15')-C(14')-H(14D)	111.0
C(13')-C(14')-H(14D)	111.0
H(14C)-C(14')-H(14D)	109.0
C(14')-C(15')-C(17')	103.2(3)
C(14')-C(15')-C(16')	107.7(3)
C(17')-C(15')-C(16')	101.2(3)
C(14')-C(15')-H(15')	114.4
C(17')-C(15')-H(15')	114.4
C(16')-C(15')-H(15')	114.4

C(15')-C(16')-C(11)	100.2(2)
C(15')-C(16')-H(16C)	111.7
C(11)-C(16')-H(16C)	111.7
C(15')-C(16')-H(16D)	111.7
C(11)-C(16')-H(16D)	111.7
H(16C)-C(16')-H(16D)	109.5
C(15')-C(17')-C(12')	93.9(3)
C(15')-C(17')-H(17C)	112.9
C(12')-C(17')-H(17C)	112.9
C(15')-C(17')-H(17D)	112.9
C(12')-C(17')-H(17D)	112.9
H(17C)-C(17')-H(17D)	110.4
C(11)-C(12")-C(17")	107.9(10)
C(11)-C(12")-C(13")	95.2(9)
C(17")-C(12")-C(13")	103.8(11)
C(11)-C(12")-H(12")	115.8
C(17")-C(12")-H(12")	115.8
C(13")-C(12")-H(12")	115.8
C(14")-C(13")-C(12")	103.1(10)
C(14")-C(13")-H(13E)	111.1
C(12")-C(13")-H(13E)	111.1
C(14")-C(13")-H(13F)	111.1
C(12")-C(13")-H(13F)	111.1
H(13E)-C(13")-H(13F)	109.1
C(15")-C(14")-C(13")	104.7(10)
C(15")-C(14")-H(14E)	110.8
C(13")-C(14")-H(14E)	110.8
C(15")-C(14")-H(14F)	110.8
C(13")-C(14")-H(14F)	110.8
H(14E)-C(14")-H(14F)	108.9
C(14")-C(15")-C(16")	107.2(12)
C(14")-C(15")-C(17")	101.3(11)
C(16")-C(15")-C(17")	97.7(11)
C(14")-C(15")-H(15")	116.1
C(16")-C(15")-H(15")	116.1
C(17")-C(15")-H(15")	116.1
C(15")-C(16")-C(11)	104.5(8)
C(15")-C(16")-H(16E)	110.9
C(11)-C(16")-H(16E)	110.9
C(15")-C(16")-H(16F)	110.9
C(11)-C(16")-H(16F)	110.9
H(16E)-C(16")-H(16F)	108.9
C(12")-C(17")-C(15")	94.9(10)
C(12")-C(17")-H(17E)	112.8
C(15")-C(17")-H(17E)	112.8
C(12")-C(17")-H(17F)	112.8
C(15")-C(17")-H(17F)	112.8
H(17E)-C(17")-H(17F)	110.2

Symmetry transformations used to generate equivalent atoms:
#1 -x,-y+1,-z

Table S12. Anisotropic displacement parameters (A^2 x 10^3) for R-008.
The anisotropic displacement factor exponent takes the form:
-2 pi^2 [h^2 a*^2 U11 + ... + 2 h k a* b* U12]

	U11	U22	U33	U23	U13	U12
Al(1)	24(1)	27(1)	35(1)	0(1)	10(1)	-1(1)
C(1)	27(1)	44(1)	34(1)	-5(1)	5(1)	4(1)
C(2)	43(1)	58(1)	50(1)	6(1)	8(1)	12(1)
C(3)	45(1)	65(1)	76(2)	6(1)	6(1)	22(1)
C(4)	31(1)	70(2)	79(2)	-14(1)	10(1)	18(1)
C(5)	25(1)	60(1)	53(1)	-23(1)	12(1)	0(1)
C(6)	27(1)	74(1)	63(1)	-29(1)	21(1)	-7(1)
C(7)	36(1)	80(2)	51(1)	-21(1)	26(1)	-21(1)
C(8)	33(1)	58(1)	42(1)	-8(1)	17(1)	-11(1)
C(9)	26(1)	42(1)	31(1)	-13(1)	11(1)	-7(1)
C(10)	24(1)	43(1)	34(1)	-14(1)	7(1)	-1(1)
C(18)	31(1)	31(1)	26(1)	-3(1)	12(1)	-5(1)
C(19)	41(1)	44(1)	52(1)	12(1)	21(1)	-3(1)
C(20)	48(1)	41(1)	52(1)	-13(1)	4(1)	5(1)
C(21)	31(1)	48(1)	56(1)	16(1)	16(1)	-1(1)
C(22)	26(1)	27(1)	30(1)	4(1)	8(1)	-3(1)
C(23)	32(1)	36(1)	32(1)	2(1)	10(1)	-5(1)
C(24)	35(1)	50(1)	32(1)	4(1)	1(1)	-4(1)
C(25)	30(1)	43(1)	46(1)	10(1)	4(1)	5(1)
C(26)	33(1)	33(1)	45(1)	4(1)	13(1)	3(1)
C(27)	30(1)	28(1)	32(1)	3(1)	9(1)	-2(1)
C(28)	41(1)	55(1)	30(1)	-6(1)	9(1)	-1(1)
C(29)	112(2)	97(2)	47(1)	-1(1)	42(1)	16(2)
C(30)	67(1)	50(1)	77(2)	-18(1)	24(1)	-6(1)
C(31)	36(1)	32(1)	33(1)	-1(1)	10(1)	3(1)
C(32)	54(1)	55(1)	43(1)	-9(1)	13(1)	-18(1)
C(33)	55(1)	67(1)	39(1)	-8(1)	21(1)	-11(1)
C(34)	52(1)	49(1)	44(1)	-8(1)	16(1)	9(1)
C(35)	39(1)	76(2)	52(1)	-16(1)	17(1)	1(1)
C(36)	51(1)	53(1)	43(1)	-11(1)	17(1)	-10(1)
N(1)	31(1)	37(1)	32(1)	-1(1)	12(1)	0(1)
N(2)	23(1)	34(1)	31(1)	-1(1)	10(1)	-2(1)
N(3)	25(1)	29(1)	28(1)	1(1)	8(1)	-2(1)
C(11)	50(1)	48(1)	36(1)	-5(1)	18(1)	-6(1)
C(12)	53(1)	53(1)	35(1)	-10(1)	21(1)	-7(1)
C(13)	49(1)	51(1)	44(1)	-5(1)	17(1)	4(1)
C(14)	49(1)	59(1)	45(2)	-10(1)	13(1)	-2(1)
C(15)	54(1)	60(1)	36(1)	-7(1)	10(1)	1(1)
C(16)	52(1)	63(2)	34(1)	-9(1)	7(1)	7(1)
C(17)	60(1)	57(1)	40(1)	-6(1)	20(1)	-4(1)
C(12')	53(2)	60(2)	37(2)	-8(2)	14(2)	2(2)
C(13')	55(2)	60(2)	41(2)	-7(2)	10(2)	0(2)
C(14')	53(2)	57(2)	42(2)	-7(2)	16(2)	1(2)
C(15')	54(2)	56(2)	38(2)	-8(2)	18(2)	0(2)
C(16')	56(2)	54(2)	35(2)	-8(2)	22(2)	-2(2)

C(17')	54(2)	57(2)	35(2)	-8(2)	14(2)	-1(2)
C(12")	51(2)	58(2)	37(2)	-7(2)	13(2)	1(2)
C(13")	53(2)	57(2)	34(2)	-7(2)	11(2)	0(2)
C(14")	55(2)	56(2)	36(2)	-7(2)	13(2)	-1(2)
C(15")	53(2)	56(2)	38(2)	-8(2)	18(2)	-1(2)
C(16")	56(2)	54(2)	35(2)	-8(2)	23(2)	-2(2)
C(17")	53(2)	58(2)	40(2)	-8(2)	15(2)	-1(2)

Crystal data and structure refinement of complex $Al_2Me_2L_2$

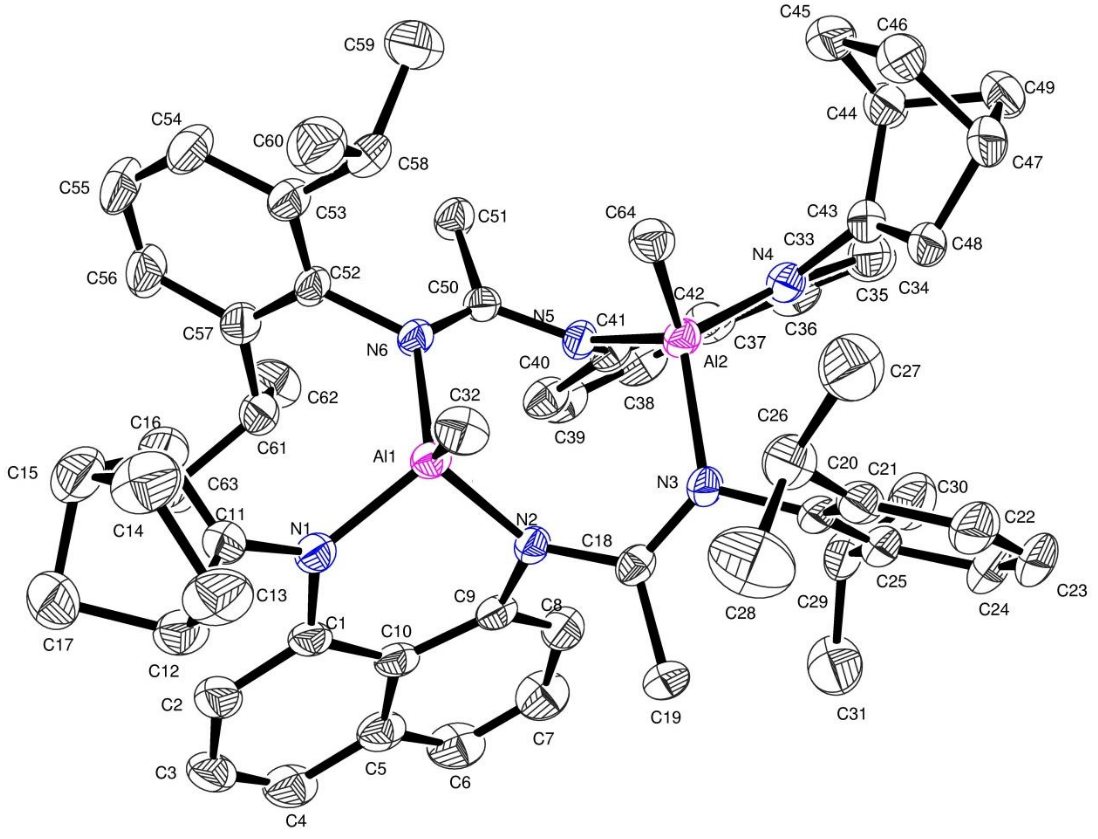

Figure S24 : Asymmetric Unit

Table S13. Crystal data and structure refinement for R-035-1.

Identification code	R-035-1
Empirical formula	C64 H76 Al2 N6
Formula weight	983.27
Temperature	193(2) K
Wavelength	0.71073 A
Crystal system, space group	Monoclinic, P 21/c
Unit cell dimensions	a = 15.3835(12) A alpha = 90 deg.<br>b = 17.3487(14) A beta = 109.571(2) deg.<br>c = 21.7171(18) A gamma = 90 deg.
Volume	5461.1(8) A^3
Z, Calculated density	4, 1.196 Mg/m^3
Absorption coefficient	0.100 mm^-1
F(000)	2112
Crystal size	0.100 x 0.060 x 0.060 mm
Theta range for data collection	2.311 to 24.776 deg.
Limiting indices	-16<=h<=18, -20<=k<=20, -25<=l<=25
Reflections collected / unique	99251 / 9345 [R(int) = 0.1592]
Completeness to theta = 24.776	99.6 %
Refinement method	Full-matrix least-squares on F^2
Data / restraints / parameters	9345 / 0 / 661
Goodness-of-fit on F^2	1.004
Final R indices [I>2sigma(I)]	R1 = 0.0580, wR2 = 0.1168
R indices (all data)	R1 = 0.1343, wR2 = 0.1485
Largest diff. peak and hole	0.345 and -0.295 e.A^-3

Table S14. Atomic coordinates (x 10^4) and equivalent isotropic displacement parameters (A^2 x 10^3) for R-035-1. U(eq) is defined as one third of the trace of the orthogonalized Uij tensor.

	x	y	z	U(eq)
Al(1)	3696(1)	6861(1)	7812(1)	26(1)
Al(2)	1354(1)	7401(1)	7625(1)	25(1)
C(1)	4861(2)	6806(2)	7002(2)	30(1)
C(2)	5655(2)	6582(2)	6879(2)	39(1)
C(3)	5737(3)	6650(2)	6259(2)	46(1)
C(4)	5024(3)	6908(2)	5742(2)	47(1)
C(5)	4198(3)	7143(2)	5835(2)	38(1)
C(6)	3453(3)	7394(2)	5289(2)	48(1)
C(7)	2638(3)	7616(2)	5349(2)	46(1)
C(8)	2552(2)	7623(2)	5969(2)	35(1)
C(9)	3269(2)	7425(2)	6523(2)	27(1)
C(10)	4111(2)	7127(2)	6472(2)	29(1)
C(11)	5554(2)	6424(2)	8113(2)	33(1)
C(12)	6469(2)	6863(2)	8357(2)	43(1)
C(13)	6528(3)	7242(2)	9007(2)	55(1)
C(14)	6604(3)	6548(2)	9474(2)	56(1)
C(15)	6593(3)	5860(2)	9024(2)	51(1)
C(16)	5647(2)	5825(2)	8522(2)	41(1)
C(17)	7143(2)	6187(2)	8617(2)	54(1)
C(18)	2696(2)	8228(2)	7163(2)	26(1)
C(19)	3107(2)	8952(2)	6980(2)	36(1)
C(20)	1592(2)	9031(2)	7412(2)	31(1)
C(21)	1831(2)	9442(2)	8000(2)	35(1)
C(22)	1395(3)	10151(2)	7995(2)	46(1)
C(23)	742(3)	10424(2)	7441(2)	50(1)
C(24)	512(3)	10015(2)	6870(2)	45(1)
C(25)	923(2)	9303(2)	6837(2)	36(1)
C(26)	2528(2)	9158(2)	8633(2)	41(1)
C(27)	2197(3)	9250(2)	9220(2)	59(1)
C(28)	3458(3)	9553(3)	8780(2)	78(2)
C(29)	605(2)	8875(2)	6190(2)	41(1)
C(30)	-447(3)	8827(2)	5898(2)	57(1)
C(31)	960(3)	9252(2)	5678(2)	59(1)
C(32)	4054(2)	7351(2)	8666(2)	39(1)
C(33)	-504(2)	7178(2)	6712(2)	30(1)
C(34)	-1438(2)	7359(2)	6484(2)	39(1)
C(35)	-2028(3)	7091(2)	5883(2)	47(1)
C(36)	-1700(3)	6655(2)	5492(2)	45(1)
C(37)	-753(2)	6462(2)	5690(2)	35(1)
C(38)	-425(3)	6039(2)	5263(2)	44(1)
C(39)	490(3)	5882(2)	5415(2)	45(1)
C(40)	1104(3)	6109(2)	6021(2)	37(1)
C(41)	803(2)	6463(2)	6483(2)	28(1)
C(42)	-138(2)	6707(2)	6313(2)	30(1)
C(43)	-371(2)	7781(2)	7728(2)	32(1)
C(44)	-1052(2)	7332(2)	7972(2)	40(1)
C(45)	-482(3)	7023(2)	8658(2)	46(1)

C(46)	-232(3)	7759(2)	9081(2)	46(1)
C(47)	-670(2)	8406(2)	8570(2)	41(1)
C(48)	-161(2)	8423(2)	8083(2)	34(1)
C(49)	-1562(2)	8007(2)	8154(2)	44(1)
C(50)	1992(2)	5917(2)	7346(2)	25(1)
C(51)	1489(2)	5155(2)	7266(2)	32(1)
C(52)	3336(2)	5217(2)	7921(2)	29(1)
C(53)	3450(2)	4985(2)	8561(2)	36(1)
C(54)	3846(2)	4263(2)	8764(2)	46(1)
C(55)	4125(3)	3800(2)	8355(2)	52(1)
C(56)	4031(2)	4039(2)	7733(2)	46(1)
C(57)	3641(2)	4758(2)	7496(2)	34(1)
C(58)	3152(2)	5482(2)	9033(2)	38(1)
C(59)	2317(3)	5138(2)	9169(2)	58(1)
C(60)	3944(3)	5612(2)	9679(2)	51(1)
C(61)	3587(2)	4997(2)	6815(2)	35(1)
C(62)	2873(3)	4534(2)	6275(2)	49(1)
C(63)	4525(2)	4908(2)	6721(2)	51(1)
C(64)	1752(2)	7192(2)	8553(2)	32(1)
N(1)	4751(2)	6671(2)	7609(1)	28(1)
N(2)	3118(2)	7539(1)	7131(1)	25(1)
N(3)	2002(2)	8266(1)	7392(1)	25(1)
N(4)	80(2)	7477(1)	7307(1)	29(1)
N(5)	1475(2)	6561(1)	7120(1)	25(1)
N(6)	2883(2)	5957(1)	7677(1)	24(1)

Table S15. Bond lengths [A] and angles [deg] for R-035-1.

Al(1)-N(1)	1.848(3)
Al(1)-N(2)	1.865(3)
Al(1)-C(32)	1.944(3)
Al(1)-N(6)	1.966(3)
Al(2)-N(4)	1.851(3)
Al(2)-N(5)	1.871(3)
Al(2)-C(64)	1.934(3)
Al(2)-N(3)	1.960(3)
C(1)-C(2)	1.391(4)
C(1)-N(1)	1.403(4)
C(1)-C(10)	1.441(5)
C(2)-C(3)	1.399(5)
C(2)-H(2)	0.9500
C(3)-C(4)	1.356(5)
C(3)-H(3)	0.9500
C(4)-C(5)	1.413(5)
C(4)-H(4)	0.9500
C(5)-C(6)	1.414(5)
C(5)-C(10)	1.433(5)
C(6)-C(7)	1.359(5)
C(6)-H(6)	0.9500
C(7)-C(8)	1.395(5)
C(7)-H(7)	0.9500
C(8)-C(9)	1.375(4)
C(8)-H(8)	0.9500
C(9)-N(2)	1.429(4)
C(9)-C(10)	1.434(4)
C(11)-C(16)	1.343(5)
C(11)-N(1)	1.415(4)
C(11)-C(12)	1.531(5)
C(12)-C(13)	1.532(5)
C(12)-C(17)	1.541(5)
C(12)-H(12)	1.0000
C(13)-C(14)	1.554(5)
C(13)-H(13A)	0.9900
C(13)-H(13B)	0.9900
C(14)-C(15)	1.540(6)
C(14)-H(14A)	0.9900
C(14)-H(14B)	0.9900
C(15)-C(16)	1.499(5)
C(15)-C(17)	1.523(5)
C(15)-H(15)	1.0000
C(16)-H(16)	0.9500
C(17)-H(17A)	0.9900
C(17)-H(17B)	0.9900
C(18)-N(3)	1.322(4)
C(18)-N(2)	1.374(4)
C(18)-C(19)	1.517(4)
C(19)-H(19A)	0.9800
C(19)-H(19B)	0.9800
C(19)-H(19C)	0.9800
C(20)-C(21)	1.400(5)
C(20)-C(25)	1.407(5)
C(20)-N(3)	1.476(4)

C(21)-C(22)	1.398(5)
C(21)-C(26)	1.515(5)
C(22)-C(23)	1.368(5)
C(22)-H(22)	0.9500
C(23)-C(24)	1.368(5)
C(23)-H(23)	0.9500
C(24)-C(25)	1.401(5)
C(24)-H(24)	0.9500
C(25)-C(29)	1.517(5)
C(26)-C(28)	1.521(5)
C(26)-C(27)	1.531(5)
C(26)-H(26)	1.0000
C(27)-H(27A)	0.9800
C(27)-H(27B)	0.9800
C(27)-H(27C)	0.9800
C(28)-H(28A)	0.9800
C(28)-H(28B)	0.9800
C(28)-H(28C)	0.9800
C(29)-C(30)	1.530(5)
C(29)-C(31)	1.539(5)
C(29)-H(29)	1.0000
C(30)-H(30A)	0.9800
C(30)-H(30B)	0.9800
C(30)-H(30C)	0.9800
C(31)-H(31A)	0.9800
C(31)-H(31B)	0.9800
C(31)-H(31C)	0.9800
C(32)-H(32A)	0.9800
C(32)-H(32B)	0.9800
C(32)-H(32C)	0.9800
C(33)-C(34)	1.390(4)
C(33)-N(4)	1.402(4)
C(33)-C(42)	1.436(4)
C(34)-C(35)	1.395(5)
C(34)-H(34)	0.9500
C(35)-C(36)	1.355(5)
C(35)-H(35)	0.9500
C(36)-C(37)	1.414(5)
C(36)-H(36)	0.9500
C(37)-C(38)	1.404(5)
C(37)-C(42)	1.432(5)
C(38)-C(39)	1.361(5)
C(38)-H(38)	0.9500
C(39)-C(40)	1.395(5)
C(39)-H(39)	0.9500
C(40)-C(41)	1.381(4)
C(40)-H(40)	0.9500
C(41)-N(5)	1.432(4)
C(41)-C(42)	1.433(4)
C(43)-C(48)	1.332(4)
C(43)-N(4)	1.422(4)
C(43)-C(44)	1.535(5)
C(44)-C(49)	1.534(5)
C(44)-C(45)	1.549(5)
C(44)-H(44)	1.0000
C(45)-C(46)	1.544(5)
C(45)-H(45A)	0.9900

C(45)-H(45B)	0.9900
C(46)-C(47)	1.564(5)
C(46)-H(46A)	0.9900
C(46)-H(46B)	0.9900
C(47)-C(48)	1.512(5)
C(47)-C(49)	1.533(5)
C(47)-H(47)	1.0000
C(48)-H(48)	0.9500
C(49)-H(49A)	0.9900
C(49)-H(49B)	0.9900
C(50)-N(6)	1.320(4)
C(50)-N(5)	1.363(4)
C(50)-C(51)	1.512(4)
C(51)-H(51A)	0.9800
C(51)-H(51B)	0.9800
C(51)-H(51C)	0.9800
C(52)-C(53)	1.400(5)
C(52)-C(57)	1.412(5)
C(52)-N(6)	1.472(4)
C(53)-C(54)	1.397(5)
C(53)-C(58)	1.523(5)
C(54)-C(55)	1.369(5)
C(54)-H(54)	0.9500
C(55)-C(56)	1.373(5)
C(55)-H(55)	0.9500
C(56)-C(57)	1.406(5)
C(56)-H(56)	0.9500
C(57)-C(61)	1.512(5)
C(58)-C(59)	1.532(5)
C(58)-C(60)	1.535(5)
C(58)-H(58)	1.0000
C(59)-H(59A)	0.9800
C(59)-H(59B)	0.9800
C(59)-H(59C)	0.9800
C(60)-H(60A)	0.9800
C(60)-H(60B)	0.9800
C(60)-H(60C)	0.9800
C(61)-C(63)	1.531(4)
C(61)-C(62)	1.537(5)
C(61)-H(61)	1.0000
C(62)-H(62A)	0.9800
C(62)-H(62B)	0.9800
C(62)-H(62C)	0.9800
C(63)-H(63A)	0.9800
C(63)-H(63B)	0.9800
C(63)-H(63C)	0.9800
C(64)-H(64A)	0.9800
C(64)-H(64B)	0.9800
C(64)-H(64C)	0.9800
N(1)-Al(1)-N(2)	98.41(12)
N(1)-Al(1)-C(32)	108.33(14)
N(2)-Al(1)-C(32)	112.87(14)
N(1)-Al(1)-N(6)	112.74(12)
N(2)-Al(1)-N(6)	105.90(12)
C(32)-Al(1)-N(6)	117.06(13)
N(4)-Al(2)-N(5)	97.89(12)

N(4)-Al(2)-C(64)	109.18(13)
N(5)-Al(2)-C(64)	113.95(13)
N(4)-Al(2)-N(3)	114.55(12)
N(5)-Al(2)-N(3)	106.67(11)
C(64)-Al(2)-N(3)	113.65(13)
C(2)-C(1)-N(1)	121.6(3)
C(2)-C(1)-C(10)	118.3(3)
N(1)-C(1)-C(10)	119.9(3)
C(1)-C(2)-C(3)	122.0(4)
C(1)-C(2)-H(2)	119.0
C(3)-C(2)-H(2)	119.0
C(4)-C(3)-C(2)	121.1(3)
C(4)-C(3)-H(3)	119.4
C(2)-C(3)-H(3)	119.4
C(3)-C(4)-C(5)	119.5(4)
C(3)-C(4)-H(4)	120.2
C(5)-C(4)-H(4)	120.2
C(4)-C(5)-C(6)	118.9(3)
C(4)-C(5)-C(10)	120.8(4)
C(6)-C(5)-C(10)	120.3(3)
C(7)-C(6)-C(5)	121.4(3)
C(7)-C(6)-H(6)	119.3
C(5)-C(6)-H(6)	119.3
C(6)-C(7)-C(8)	118.9(4)
C(6)-C(7)-H(7)	120.5
C(8)-C(7)-H(7)	120.5
C(9)-C(8)-C(7)	122.3(3)
C(9)-C(8)-H(8)	118.8
C(7)-C(8)-H(8)	118.8
C(8)-C(9)-N(2)	116.4(3)
C(8)-C(9)-C(10)	120.2(3)
N(2)-C(9)-C(10)	123.5(3)
C(5)-C(10)-C(9)	116.5(3)
C(5)-C(10)-C(1)	118.0(3)
C(9)-C(10)-C(1)	125.5(3)
C(16)-C(11)-N(1)	128.1(3)
C(16)-C(11)-C(12)	105.9(3)
N(1)-C(11)-C(12)	125.5(3)
C(13)-C(12)-C(11)	107.2(3)
C(13)-C(12)-C(17)	99.4(3)
C(11)-C(12)-C(17)	100.1(3)
C(13)-C(12)-H(12)	115.9
C(11)-C(12)-H(12)	115.9
C(17)-C(12)-H(12)	115.9
C(12)-C(13)-C(14)	103.8(3)
C(12)-C(13)-H(13A)	111.0
C(14)-C(13)-H(13A)	111.0
C(12)-C(13)-H(13B)	111.0
C(14)-C(13)-H(13B)	111.0
H(13A)-C(13)-H(13B)	109.0
C(15)-C(14)-C(13)	101.7(3)
C(15)-C(14)-H(14A)	111.4
C(13)-C(14)-H(14A)	111.4
C(15)-C(14)-H(14B)	111.4
C(13)-C(14)-H(14B)	111.4
H(14A)-C(14)-H(14B)	109.3
C(16)-C(15)-C(17)	100.6(3)

C(16)-C(15)-C(14)	107.1(3)
C(17)-C(15)-C(14)	100.9(3)
C(16)-C(15)-H(15)	115.4
C(17)-C(15)-H(15)	115.4
C(14)-C(15)-H(15)	115.4
C(11)-C(16)-C(15)	108.2(3)
C(11)-C(16)-H(16)	125.9
C(15)-C(16)-H(16)	125.9
C(15)-C(17)-C(12)	93.5(3)
C(15)-C(17)-H(17A)	113.0
C(12)-C(17)-H(17A)	113.0
C(15)-C(17)-H(17B)	113.0
C(12)-C(17)-H(17B)	113.0
H(17A)-C(17)-H(17B)	110.4
N(3)-C(18)-N(2)	121.2(3)
N(3)-C(18)-C(19)	121.1(3)
N(2)-C(18)-C(19)	117.5(3)
C(18)-C(19)-H(19A)	109.5
C(18)-C(19)-H(19B)	109.5
H(19A)-C(19)-H(19B)	109.5
C(18)-C(19)-H(19C)	109.5
H(19A)-C(19)-H(19C)	109.5
H(19B)-C(19)-H(19C)	109.5
C(21)-C(20)-C(25)	122.0(3)
C(21)-C(20)-N(3)	120.0(3)
C(25)-C(20)-N(3)	117.9(3)
C(22)-C(21)-C(20)	117.5(3)
C(22)-C(21)-C(26)	119.0(3)
C(20)-C(21)-C(26)	123.5(3)
C(23)-C(22)-C(21)	121.3(4)
C(23)-C(22)-H(22)	119.4
C(21)-C(22)-H(22)	119.4
C(24)-C(23)-C(22)	120.6(4)
C(24)-C(23)-H(23)	119.7
C(22)-C(23)-H(23)	119.7
C(23)-C(24)-C(25)	121.4(4)
C(23)-C(24)-H(24)	119.3
C(25)-C(24)-H(24)	119.3
C(24)-C(25)-C(20)	117.2(3)
C(24)-C(25)-C(29)	118.0(3)
C(20)-C(25)-C(29)	124.8(3)
C(21)-C(26)-C(28)	111.9(3)
C(21)-C(26)-C(27)	112.9(3)
C(28)-C(26)-C(27)	109.3(3)
C(21)-C(26)-H(26)	107.5
C(28)-C(26)-H(26)	107.5
C(27)-C(26)-H(26)	107.5
C(26)-C(27)-H(27A)	109.5
C(26)-C(27)-H(27B)	109.5
H(27A)-C(27)-H(27B)	109.5
C(26)-C(27)-H(27C)	109.5
H(27A)-C(27)-H(27C)	109.5
H(27B)-C(27)-H(27C)	109.5
C(26)-C(28)-H(28A)	109.5
C(26)-C(28)-H(28B)	109.5
H(28A)-C(28)-H(28B)	109.5
C(26)-C(28)-H(28C)	109.5

H(28A)-C(28)-H(28C)	109.5
H(28B)-C(28)-H(28C)	109.5
C(25)-C(29)-C(30)	112.2(3)
C(25)-C(29)-C(31)	112.2(3)
C(30)-C(29)-C(31)	107.8(3)
C(25)-C(29)-H(29)	108.2
C(30)-C(29)-H(29)	108.2
C(31)-C(29)-H(29)	108.2
C(29)-C(30)-H(30A)	109.5
C(29)-C(30)-H(30B)	109.5
H(30A)-C(30)-H(30B)	109.5
C(29)-C(30)-H(30C)	109.5
H(30A)-C(30)-H(30C)	109.5
H(30B)-C(30)-H(30C)	109.5
C(29)-C(31)-H(31A)	109.5
C(29)-C(31)-H(31B)	109.5
H(31A)-C(31)-H(31B)	109.5
C(29)-C(31)-H(31C)	109.5
H(31A)-C(31)-H(31C)	109.5
H(31B)-C(31)-H(31C)	109.5
Al(1)-C(32)-H(32A)	109.5
Al(1)-C(32)-H(32B)	109.5
H(32A)-C(32)-H(32B)	109.5
Al(1)-C(32)-H(32C)	109.5
H(32A)-C(32)-H(32C)	109.5
H(32B)-C(32)-H(32C)	109.5
C(34)-C(33)-N(4)	120.8(3)
C(34)-C(33)-C(42)	118.7(3)
N(4)-C(33)-C(42)	120.4(3)
C(33)-C(34)-C(35)	121.9(4)
C(33)-C(34)-H(34)	119.0
C(35)-C(34)-H(34)	119.0
C(36)-C(35)-C(34)	120.7(4)
C(36)-C(35)-H(35)	119.7
C(34)-C(35)-H(35)	119.7
C(35)-C(36)-C(37)	120.2(4)
C(35)-C(36)-H(36)	119.9
C(37)-C(36)-H(36)	119.9
C(38)-C(37)-C(36)	119.0(3)
C(38)-C(37)-C(42)	120.7(3)
C(36)-C(37)-C(42)	120.3(3)
C(39)-C(38)-C(37)	121.0(3)
C(39)-C(38)-H(38)	119.5
C(37)-C(38)-H(38)	119.5
C(38)-C(39)-C(40)	119.4(4)
C(38)-C(39)-H(39)	120.3
C(40)-C(39)-H(39)	120.3
C(41)-C(40)-C(39)	121.7(3)
C(41)-C(40)-H(40)	119.1
C(39)-C(40)-H(40)	119.1
C(40)-C(41)-N(5)	116.4(3)
C(40)-C(41)-C(42)	120.0(3)
N(5)-C(41)-C(42)	123.6(3)
C(37)-C(42)-C(41)	116.5(3)
C(37)-C(42)-C(33)	118.0(3)
C(41)-C(42)-C(33)	125.4(3)
C(48)-C(43)-N(4)	127.3(3)

C(48)-C(43)-C(44)	106.6(3)
N(4)-C(43)-C(44)	124.8(3)
C(49)-C(44)-C(43)	99.7(3)
C(49)-C(44)-C(45)	101.0(3)
C(43)-C(44)-C(45)	106.0(3)
C(49)-C(44)-H(44)	116.0
C(43)-C(44)-H(44)	116.0
C(45)-C(44)-H(44)	116.0
C(46)-C(45)-C(44)	103.6(3)
C(46)-C(45)-H(45A)	111.0
C(44)-C(45)-H(45A)	111.0
C(46)-C(45)-H(45B)	111.0
C(44)-C(45)-H(45B)	111.0
H(45A)-C(45)-H(45B)	109.0
C(45)-C(46)-C(47)	102.1(3)
C(45)-C(46)-H(46A)	111.4
C(47)-C(46)-H(46A)	111.4
C(45)-C(46)-H(46B)	111.4
C(47)-C(46)-H(46B)	111.4
H(46A)-C(46)-H(46B)	109.2
C(48)-C(47)-C(49)	99.7(3)
C(48)-C(47)-C(46)	108.0(3)
C(49)-C(47)-C(46)	100.5(3)
C(48)-C(47)-H(47)	115.5
C(49)-C(47)-H(47)	115.5
C(46)-C(47)-H(47)	115.5
C(43)-C(48)-C(47)	107.9(3)
C(43)-C(48)-H(48)	126.1
C(47)-C(48)-H(48)	126.1
C(44)-C(49)-C(47)	93.6(3)
C(44)-C(49)-H(49A)	113.0
C(47)-C(49)-H(49A)	113.0
C(44)-C(49)-H(49B)	113.0
C(47)-C(49)-H(49B)	113.0
H(49A)-C(49)-H(49B)	110.4
N(6)-C(50)-N(5)	121.8(3)
N(6)-C(50)-C(51)	120.4(3)
N(5)-C(50)-C(51)	117.4(3)
C(50)-C(51)-H(51A)	109.5
C(50)-C(51)-H(51B)	109.5
H(51A)-C(51)-H(51B)	109.5
C(50)-C(51)-H(51C)	109.5
H(51A)-C(51)-H(51C)	109.5
H(51B)-C(51)-H(51C)	109.5
C(53)-C(52)-C(57)	121.8(3)
C(53)-C(52)-N(6)	119.7(3)
C(57)-C(52)-N(6)	118.4(3)
C(54)-C(53)-C(52)	117.9(3)
C(54)-C(53)-C(58)	119.5(3)
C(52)-C(53)-C(58)	122.6(3)
C(55)-C(54)-C(53)	121.3(4)
C(55)-C(54)-H(54)	119.3
C(53)-C(54)-H(54)	119.3
C(54)-C(55)-C(56)	120.5(4)
C(54)-C(55)-H(55)	119.7
C(56)-C(55)-H(55)	119.7
C(55)-C(56)-C(57)	121.3(4)

C(55)-C(56)-H(56)	119.4
C(57)-C(56)-H(56)	119.4
C(56)-C(57)-C(52)	117.1(3)
C(56)-C(57)-C(61)	118.6(3)
C(52)-C(57)-C(61)	124.3(3)
C(53)-C(58)-C(59)	111.8(3)
C(53)-C(58)-C(60)	111.9(3)
C(59)-C(58)-C(60)	109.9(3)
C(53)-C(58)-H(58)	107.7
C(59)-C(58)-H(58)	107.7
C(60)-C(58)-H(58)	107.7
C(58)-C(59)-H(59A)	109.5
C(58)-C(59)-H(59B)	109.5
H(59A)-C(59)-H(59B)	109.5
C(58)-C(59)-H(59C)	109.5
H(59A)-C(59)-H(59C)	109.5
H(59B)-C(59)-H(59C)	109.5
C(58)-C(60)-H(60A)	109.5
C(58)-C(60)-H(60B)	109.5
H(60A)-C(60)-H(60B)	109.5
C(58)-C(60)-H(60C)	109.5
H(60A)-C(60)-H(60C)	109.5
H(60B)-C(60)-H(60C)	109.5
C(57)-C(61)-C(63)	110.9(3)
C(57)-C(61)-C(62)	113.2(3)
C(63)-C(61)-C(62)	108.1(3)
C(57)-C(61)-H(61)	108.2
C(63)-C(61)-H(61)	108.2
C(62)-C(61)-H(61)	108.2
C(61)-C(62)-H(62A)	109.5
C(61)-C(62)-H(62B)	109.5
H(62A)-C(62)-H(62B)	109.5
C(61)-C(62)-H(62C)	109.5
H(62A)-C(62)-H(62C)	109.5
H(62B)-C(62)-H(62C)	109.5
C(61)-C(63)-H(63A)	109.5
C(61)-C(63)-H(63B)	109.5
H(63A)-C(63)-H(63B)	109.5
C(61)-C(63)-H(63C)	109.5
H(63A)-C(63)-H(63C)	109.5
H(63B)-C(63)-H(63C)	109.5
Al(2)-C(64)-H(64A)	109.5
Al(2)-C(64)-H(64B)	109.5
H(64A)-C(64)-H(64B)	109.5
Al(2)-C(64)-H(64C)	109.5
H(64A)-C(64)-H(64C)	109.5
H(64B)-C(64)-H(64C)	109.5
C(1)-N(1)-C(11)	115.3(3)
C(1)-N(1)-Al(1)	125.9(2)
C(11)-N(1)-Al(1)	118.6(2)
C(18)-N(2)-C(9)	113.1(2)
C(18)-N(2)-Al(1)	128.0(2)
C(9)-N(2)-Al(1)	117.7(2)
C(18)-N(3)-C(20)	117.6(2)
C(18)-N(3)-Al(2)	127.1(2)
C(20)-N(3)-Al(2)	114.97(19)
C(33)-N(4)-C(43)	115.4(3)

C(33)-N(4)-Al(2)	126.1(2)
C(43)-N(4)-Al(2)	118.0(2)
C(50)-N(5)-C(41)	113.3(3)
C(50)-N(5)-Al(2)	126.0(2)
C(41)-N(5)-Al(2)	118.5(2)
C(50)-N(6)-C(52)	115.5(2)
C(50)-N(6)-Al(1)	128.0(2)
C(52)-N(6)-Al(1)	116.21(19)

Symmetry transformations used to generate equivalent atoms:

Table S16. Anisotropic displacement parameters (A^2 x 10^3) for R-035-1.
The anisotropic displacement factor exponent takes the form:
-2 pi^2 [h^2 a*^2 U11 + ... + 2 h k a* b* U12]

	U11	U22	U33	U23	U13	U12
Al(1)	28(1)	22(1)	28(1)	1(1)	10(1)	-2(1)
Al(2)	30(1)	19(1)	28(1)	-1(1)	11(1)	0(1)
C(1)	38(2)	20(2)	40(2)	-2(2)	22(2)	-5(2)
C(2)	40(2)	30(2)	53(3)	-1(2)	23(2)	-4(2)
C(3)	53(3)	31(2)	72(3)	-6(2)	45(3)	-4(2)
C(4)	69(3)	38(2)	51(3)	-3(2)	42(2)	-2(2)
C(5)	55(3)	25(2)	41(2)	-4(2)	26(2)	-5(2)
C(6)	71(3)	51(3)	28(2)	-2(2)	26(2)	-6(2)
C(7)	59(3)	50(3)	27(2)	2(2)	12(2)	0(2)
C(8)	44(2)	33(2)	32(2)	1(2)	16(2)	4(2)
C(9)	37(2)	20(2)	25(2)	1(2)	13(2)	-2(2)
C(10)	39(2)	20(2)	30(2)	-2(2)	17(2)	-7(2)
C(11)	29(2)	30(2)	41(2)	2(2)	14(2)	0(2)
C(12)	35(2)	41(2)	49(3)	10(2)	10(2)	-7(2)
C(13)	46(3)	48(3)	60(3)	2(2)	5(2)	-20(2)
C(14)	49(3)	59(3)	45(3)	10(2)	-2(2)	-10(2)
C(15)	40(2)	42(2)	61(3)	17(2)	3(2)	-1(2)
C(16)	30(2)	34(2)	56(3)	4(2)	9(2)	-6(2)
C(17)	28(2)	51(3)	76(3)	7(2)	9(2)	-3(2)
C(18)	34(2)	23(2)	22(2)	1(2)	8(2)	2(2)
C(19)	40(2)	23(2)	48(2)	3(2)	20(2)	-4(2)
C(20)	39(2)	17(2)	44(2)	0(2)	23(2)	-1(2)
C(21)	41(2)	24(2)	45(2)	-7(2)	24(2)	-6(2)
C(22)	64(3)	26(2)	56(3)	-5(2)	31(2)	-1(2)
C(23)	69(3)	19(2)	72(3)	0(2)	37(3)	8(2)
C(24)	56(3)	24(2)	55(3)	9(2)	22(2)	8(2)
C(25)	44(2)	23(2)	48(3)	4(2)	24(2)	3(2)
C(26)	49(2)	32(2)	45(3)	-12(2)	18(2)	-6(2)
C(27)	74(3)	53(3)	53(3)	-7(2)	26(2)	1(2)
C(28)	60(3)	93(4)	78(4)	-13(3)	19(3)	-27(3)
C(29)	46(2)	31(2)	44(2)	5(2)	14(2)	12(2)
C(30)	49(3)	47(3)	67(3)	10(2)	10(2)	9(2)
C(31)	63(3)	71(3)	44(3)	11(2)	17(2)	14(2)
C(32)	38(2)	41(2)	38(2)	-2(2)	14(2)	-5(2)
C(33)	29(2)	25(2)	33(2)	6(2)	7(2)	-2(2)
C(34)	31(2)	41(2)	44(3)	9(2)	11(2)	5(2)
C(35)	28(2)	57(3)	49(3)	18(2)	2(2)	-1(2)
C(36)	38(2)	47(3)	38(2)	10(2)	-4(2)	-7(2)
C(37)	40(2)	31(2)	28(2)	10(2)	4(2)	-4(2)
C(38)	58(3)	40(2)	23(2)	0(2)	0(2)	-8(2)
C(39)	67(3)	38(2)	29(2)	-5(2)	17(2)	-4(2)
C(40)	49(2)	28(2)	33(2)	0(2)	13(2)	-2(2)
C(41)	36(2)	18(2)	26(2)	-1(2)	8(2)	-4(2)
C(42)	31(2)	25(2)	30(2)	5(2)	7(2)	-2(2)
C(43)	30(2)	28(2)	39(2)	2(2)	14(2)	4(2)

C(44)	43(2)	30(2)	56(3)	-5(2)	27(2)	-3(2)
C(45)	56(3)	33(2)	59(3)	3(2)	34(2)	0(2)
C(46)	61(3)	38(2)	50(3)	3(2)	33(2)	7(2)
C(47)	47(2)	29(2)	55(3)	-3(2)	28(2)	7(2)
C(48)	37(2)	23(2)	49(2)	-1(2)	23(2)	2(2)
C(49)	40(2)	39(2)	65(3)	-1(2)	33(2)	1(2)
C(50)	29(2)	22(2)	26(2)	0(2)	13(2)	0(2)
C(51)	31(2)	23(2)	41(2)	0(2)	9(2)	-2(2)
C(52)	24(2)	18(2)	42(2)	4(2)	9(2)	-1(1)
C(53)	33(2)	27(2)	47(3)	10(2)	13(2)	1(2)
C(54)	46(2)	34(2)	56(3)	18(2)	13(2)	6(2)
C(55)	55(3)	31(2)	68(3)	15(2)	17(2)	17(2)
C(56)	44(2)	27(2)	67(3)	1(2)	20(2)	8(2)
C(57)	27(2)	24(2)	49(3)	1(2)	11(2)	-4(2)
C(58)	42(2)	36(2)	38(2)	17(2)	15(2)	10(2)
C(59)	48(3)	74(3)	59(3)	22(2)	25(2)	5(2)
C(60)	52(3)	52(3)	48(3)	7(2)	14(2)	1(2)
C(61)	37(2)	23(2)	50(3)	-4(2)	19(2)	1(2)
C(62)	49(2)	47(2)	55(3)	-13(2)	23(2)	-11(2)
C(63)	48(3)	38(2)	76(3)	-10(2)	33(2)	-4(2)
C(64)	35(2)	31(2)	32(2)	-2(2)	14(2)	-1(2)
N(1)	27(2)	25(2)	31(2)	3(1)	8(1)	-1(1)
N(2)	31(2)	19(2)	27(2)	2(1)	13(1)	3(1)
N(3)	31(2)	19(2)	29(2)	-2(1)	13(1)	2(1)
N(4)	33(2)	23(2)	34(2)	-1(1)	14(1)	0(1)
N(5)	25(2)	22(2)	29(2)	2(1)	8(1)	3(1)
N(6)	24(2)	19(1)	30(2)	2(1)	8(1)	-1(1)

Crystal data and structure refinement of complex AlI_2L

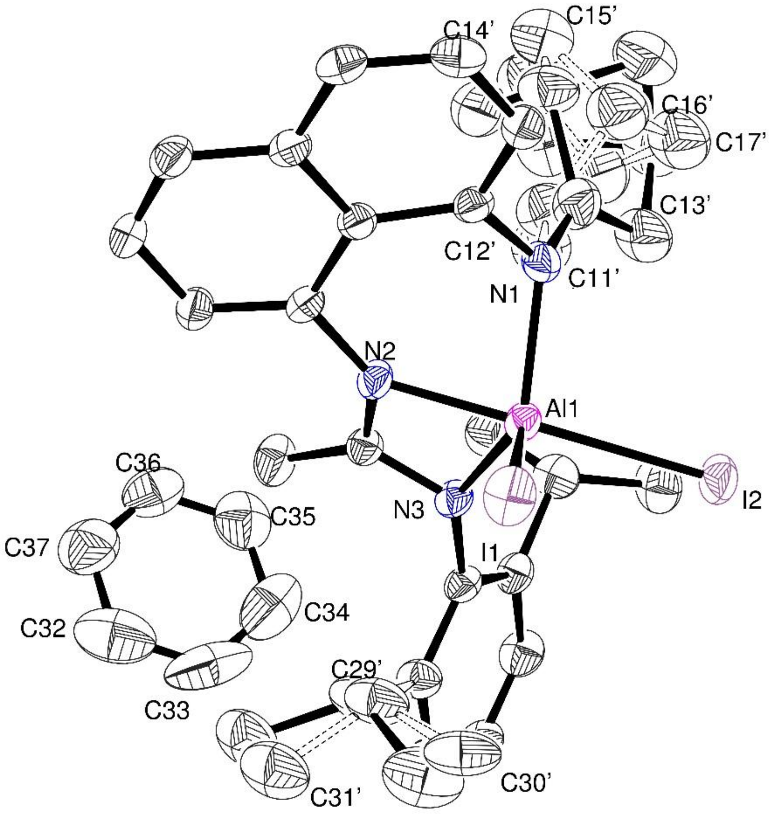

Figure S25 : Asymmetric Unit

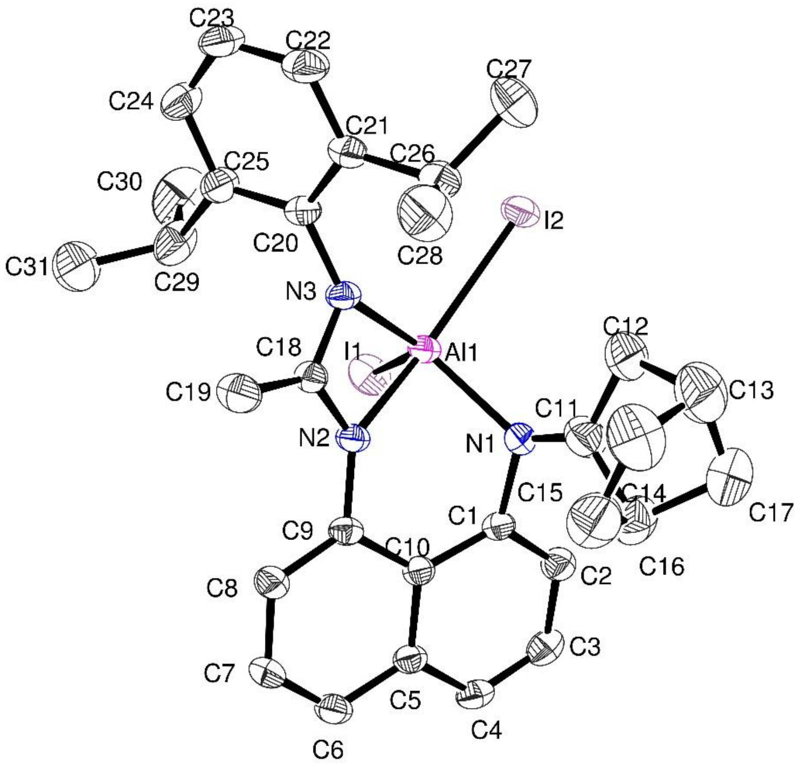

Figure S26 : Compound

Table S17. Crystal data and structure refinement for R-057.

Identification code	R-057
Empirical formula	C31 H36 Al I2 N3, C6 H6
Formula weight	809.52
Temperature	193(2) K
Wavelength	0.71073 A
Crystal system, space group	Monoclinic, P 21/c
Unit cell dimensions	a = 20.587(5) A alpha = 90 deg.<br>b = 10.690(2) A beta = 108.015(13) deg.<br>c = 17.233(4) A gamma = 90 deg.
Volume	3606.6(14) A^3
Z, Calculated density	4, 1.491 Mg/m^3
Absorption coefficient	1.797 mm^-1
F(000)	1616
Crystal size	0.180 x 0.080 x 0.060 mm
Theta range for data collection	2.704 to 30.794 deg.
Limiting indices	-29<=h<=29, -15<=k<=15,-24<=l<=24
Reflections collected / unique	74533 / 11279 [R(int) = 0.0650]
Completeness to theta = 25.242	99.8 %
Refinement method	Full-matrix least-squares on F^2
Data / restraints / parameters	11279 / 384 / 483
Goodness-of-fit on F^2	1.032
Final R indices [I>2sigma(I)]	R1 = 0.0422, wR2 = 0.0845
R indices (all data)	R1 = 0.0833, wR2 = 0.0994
Largest diff. peak and hole	1.125 and -0.665 e.A^-3

Table S18. Atomic coordinates (x 10^4) and equivalent isotropic displacement parameters (A^2 x 10^3) for R-057. U(eq) is defined as one third of the trace of the orthogonalized Uij tensor.

	x	y	z	U(eq)
C(1)	4532(2)	4133(3)	7786(2)	26(1)
C(2)	5124(2)	4774(3)	7855(2)	31(1)
C(3)	5763(2)	4276(3)	8311(2)	34(1)
C(4)	5798(2)	3171(3)	8714(2)	30(1)
C(5)	5195(2)	2472(3)	8655(2)	26(1)
C(6)	5227(2)	1312(3)	9075(2)	32(1)
C(7)	4648(2)	642(3)	8991(2)	32(1)
C(8)	4010(2)	1039(3)	8469(2)	30(1)
C(9)	3954(2)	2146(3)	8041(2)	24(1)
C(10)	4546(2)	2924(3)	8155(2)	24(1)
N(1)	3878(1)	4754(2)	7367(2)	30(1)
C(11)	3724(3)	5713(4)	7753(3)	44(1)
C(12)	3121(3)	6551(5)	7481(3)	56(1)
C(13)	3189(4)	7447(6)	8177(4)	72(2)
C(14)	3020(4)	6523(6)	8805(5)	72(1)
C(15)	3621(3)	5704(6)	9081(3)	65(1)
C(16)	4120(3)	6152(5)	8627(3)	52(1)
C(17)	3916(4)	7548(6)	8562(4)	78(2)
C(11')	3650(10)	5420(20)	7875(15)	59(3)
C(12')	2954(9)	5570(20)	7949(17)	69(3)
C(13')	2944(9)	6840(20)	8313(16)	70(3)
C(14')	3453(14)	6580(30)	9189(12)	69(2)
C(15')	4141(11)	6560(30)	9102(12)	69(3)
C(16')	4020(10)	6691(17)	8177(13)	63(2)
C(17')	3396(14)	7574(19)	7989(17)	69(3)
C(18)	2718(2)	2273(3)	7227(2)	25(1)
C(19)	2382(2)	1440(4)	7686(2)	40(1)
C(20)	1683(2)	2695(3)	6093(2)	26(1)
C(21)	1185(2)	3581(3)	6113(2)	30(1)
C(22)	506(2)	3339(4)	5660(2)	39(1)
C(23)	321(2)	2263(4)	5203(2)	42(1)
C(24)	815(2)	1409(4)	5181(2)	42(1)
C(26)	1370(2)	4742(3)	6640(2)	38(1)
C(27)	1050(2)	5934(4)	6189(3)	51(1)
C(28)	1170(3)	4587(4)	7420(3)	60(1)
C(25)	1502(2)	1591(3)	5619(2)	33(1)
C(29)	2029(7)	635(11)	5556(9)	47(2)
C(30)	2090(8)	640(17)	4695(8)	75(3)
C(31)	1864(7)	-704(9)	5819(11)	67(3)
C(29')	2050(14)	760(20)	5461(18)	51(3)
C(30')	2187(12)	1140(20)	4669(13)	58(4)
C(31')	1863(13)	-653(18)	5458(18)	65(4)
C(32)	852(3)	-993(7)	7654(4)	88(2)
C(33)	535(3)	-114(8)	7070(3)	93(2)
C(34)	427(3)	1071(7)	7308(4)	82(2)
C(35)	634(2)	1384(5)	8121(3)	68(1)

C(36)	944(2)	514(5)	8690(3)	64(1)
C(37)	1055(3)	-662(6)	8469(3)	74(2)
Al(1)	3240(1)	3692(1)	6485(1)	26(1)
N(2)	3378(1)	2543(2)	7414(2)	27(1)
N(3)	2388(1)	2913(2)	6543(2)	26(1)
I(1)	3948(1)	2566(1)	5710(1)	42(1)
I(2)	2698(1)	5431(1)	5392(1)	40(1)

Table S19. Bond lengths [A] and angles [deg] for R-057.

C(1)-C(2)	1.370(4)
C(1)-C(10)	1.438(4)
C(1)-N(1)	1.474(4)
C(2)-C(3)	1.410(5)
C(2)-H(2)	0.9500
C(3)-C(4)	1.361(5)
C(3)-H(3)	0.9500
C(4)-C(5)	1.424(4)
C(4)-H(4)	0.9500
C(5)-C(6)	1.428(4)
C(5)-C(10)	1.431(4)
C(6)-C(7)	1.359(5)
C(6)-H(6)	0.9500
C(7)-C(8)	1.408(4)
C(7)-H(7)	0.9500
C(8)-C(9)	1.380(4)
C(8)-H(8)	0.9500
C(9)-N(2)	1.401(4)
C(9)-C(10)	1.437(4)
N(1)-C(11)	1.313(5)
N(1)-C(11')	1.324(10)
N(1)-Al(1)	2.022(3)
C(11)-C(12)	1.484(6)
C(11)-C(16)	1.547(7)
C(12)-C(13)	1.507(8)
C(12)-H(12A)	0.9900
C(12)-H(12B)	0.9900
C(13)-C(17)	1.441(9)
C(13)-C(14)	1.581(9)
C(13)-H(13)	1.0000
C(14)-C(15)	1.470(9)
C(14)-H(14A)	0.9900
C(14)-H(14B)	0.9900
C(15)-C(16)	1.547(8)
C(15)-H(15A)	0.9900
C(15)-H(15B)	0.9900
C(16)-C(17)	1.545(8)
C(16)-H(16)	1.0000
C(17)-H(17A)	0.9900
C(17)-H(17B)	0.9900
C(11')-C(12')	1.486(11)
C(11')-C(16')	1.564(11)
C(12')-C(13')	1.502(12)
C(12')-H(12C)	0.9900
C(12')-H(12D)	0.9900
C(13')-C(17')	1.453(13)
C(13')-C(14')	1.575(12)
C(13')-H(13')	1.0000
C(14')-C(15')	1.470(13)
C(14')-H(14C)	0.9900
C(14')-H(14D)	0.9900
C(15')-C(16')	1.541(12)
C(15')-H(15C)	0.9900
C(15')-H(15D)	0.9900

C(16')-C(17')	1.545(12)
C(16')-H(16')	1.0000
C(17')-H(17C)	0.9900
C(17')-H(17D)	0.9900
C(18)-N(2)	1.327(4)
C(18)-N(3)	1.351(4)
C(18)-C(19)	1.494(4)
C(18)-Al(1)	2.438(3)
C(19)-H(19A)	0.9800
C(19)-H(19B)	0.9800
C(19)-H(19C)	0.9800
C(20)-C(21)	1.405(4)
C(20)-C(25)	1.418(5)
C(20)-N(3)	1.437(4)
C(21)-C(22)	1.398(4)
C(21)-C(26)	1.515(5)
C(22)-C(23)	1.379(5)
C(22)-H(22)	0.9500
C(23)-C(24)	1.376(5)
C(23)-H(23)	0.9500
C(24)-C(25)	1.395(5)
C(24)-H(24)	0.9500
C(26)-C(28)	1.531(6)
C(26)-C(27)	1.532(5)
C(26)-H(26)	1.0000
C(27)-H(27A)	0.9800
C(27)-H(27B)	0.9800
C(27)-H(27C)	0.9800
C(28)-H(28A)	0.9800
C(28)-H(28B)	0.9800
C(28)-H(28C)	0.9800
C(25)-C(29)	1.518(9)
C(25)-C(29')	1.522(14)
C(29)-C(30)	1.528(11)
C(29)-C(31)	1.570(10)
C(29)-H(29)	1.0000
C(30)-H(30A)	0.9800
C(30)-H(30B)	0.9800
C(30)-H(30C)	0.9800
C(31)-H(31A)	0.9800
C(31)-H(31B)	0.9800
C(31)-H(31C)	0.9800
C(29')-C(30')	1.534(15)
C(29')-C(31')	1.561(16)
C(29')-H(29')	1.0000
C(30')-H(30D)	0.9800
C(30')-H(30E)	0.9800
C(30')-H(30F)	0.9800
C(31')-H(31D)	0.9800
C(31')-H(31E)	0.9800
C(31')-H(31F)	0.9800
C(32)-C(37)	1.382(8)
C(32)-C(33)	1.384(9)
C(32)-H(32)	0.9500
C(33)-C(34)	1.371(9)
C(33)-H(33)	0.9500
C(34)-C(35)	1.374(8)

C(34)-H(34)	0.9500
C(35)-C(36)	1.360(7)
C(35)-H(35)	0.9500
C(36)-C(37)	1.353(7)
C(36)-H(36)	0.9500
C(37)-H(37)	0.9500
Al(1)-N(2)	1.968(3)
Al(1)-N(3)	1.972(3)
Al(1)-I(1)	2.5611(11)
Al(1)-I(2)	2.6353(10)
C(2)-C(1)-C(10)	121.0(3)
C(2)-C(1)-N(1)	118.1(3)
C(10)-C(1)-N(1)	120.8(3)
C(1)-C(2)-C(3)	120.7(3)
C(1)-C(2)-H(2)	119.6
C(3)-C(2)-H(2)	119.6
C(4)-C(3)-C(2)	120.4(3)
C(4)-C(3)-H(3)	119.8
C(2)-C(3)-H(3)	119.8
C(3)-C(4)-C(5)	120.7(3)
C(3)-C(4)-H(4)	119.7
C(5)-C(4)-H(4)	119.7
C(4)-C(5)-C(6)	121.1(3)
C(4)-C(5)-C(10)	119.8(3)
C(6)-C(5)-C(10)	119.0(3)
C(7)-C(6)-C(5)	120.3(3)
C(7)-C(6)-H(6)	119.9
C(5)-C(6)-H(6)	119.9
C(6)-C(7)-C(8)	121.5(3)
C(6)-C(7)-H(7)	119.3
C(8)-C(7)-H(7)	119.3
C(9)-C(8)-C(7)	120.5(3)
C(9)-C(8)-H(8)	119.7
C(7)-C(8)-H(8)	119.7
C(8)-C(9)-N(2)	125.6(3)
C(8)-C(9)-C(10)	119.7(3)
N(2)-C(9)-C(10)	114.3(3)
C(5)-C(10)-C(9)	118.8(3)
C(5)-C(10)-C(1)	117.1(3)
C(9)-C(10)-C(1)	124.0(3)
C(11)-N(1)-C(1)	115.5(3)
C(11')-N(1)-C(1)	112.2(12)
C(11)-N(1)-Al(1)	128.0(3)
C(11')-N(1)-Al(1)	121.2(8)
C(1)-N(1)-Al(1)	113.4(2)
N(1)-C(11)-C(12)	128.4(4)
N(1)-C(11)-C(16)	126.3(4)
C(12)-C(11)-C(16)	105.2(4)
C(11)-C(12)-C(13)	105.7(4)
C(11)-C(12)-H(12A)	110.6
C(13)-C(12)-H(12A)	110.6
C(11)-C(12)-H(12B)	110.6
C(13)-C(12)-H(12B)	110.6
H(12A)-C(12)-H(12B)	108.7
C(17)-C(13)-C(12)	104.0(5)
C(17)-C(13)-C(14)	98.5(6)

C(12)-C(13)-C(14)	99.3(5)
C(17)-C(13)-H(13)	117.3
C(12)-C(13)-H(13)	117.3
C(14)-C(13)-H(13)	117.3
C(15)-C(14)-C(13)	104.6(5)
C(15)-C(14)-H(14A)	110.8
C(13)-C(14)-H(14A)	110.8
C(15)-C(14)-H(14B)	110.8
C(13)-C(14)-H(14B)	110.8
H(14A)-C(14)-H(14B)	108.9
C(14)-C(15)-C(16)	106.0(5)
C(14)-C(15)-H(15A)	110.5
C(16)-C(15)-H(15A)	110.5
C(14)-C(15)-H(15B)	110.5
C(16)-C(15)-H(15B)	110.5
H(15A)-C(15)-H(15B)	108.7
C(17)-C(16)-C(11)	99.9(5)
C(17)-C(16)-C(15)	97.3(5)
C(11)-C(16)-C(15)	99.6(4)
C(17)-C(16)-H(16)	118.6
C(11)-C(16)-H(16)	118.6
C(15)-C(16)-H(16)	118.6
C(13)-C(17)-C(16)	100.5(5)
C(13)-C(17)-H(17A)	111.7
C(16)-C(17)-H(17A)	111.7
C(13)-C(17)-H(17B)	111.7
C(16)-C(17)-H(17B)	111.7
H(17A)-C(17)-H(17B)	109.4
N(1)-C(11')-C(12')	131.5(14)
N(1)-C(11')-C(16')	116.9(13)
C(12')-C(11')-C(16')	105.4(8)
C(11')-C(12')-C(13')	105.5(9)
C(11')-C(12')-H(12C)	110.6
C(13')-C(12')-H(12C)	110.6
C(11')-C(12')-H(12D)	110.6
C(13')-C(12')-H(12D)	110.6
H(12C)-C(12')-H(12D)	108.8
C(17')-C(13')-C(12')	103.6(11)
C(17')-C(13')-C(14')	98.7(11)
C(12')-C(13')-C(14')	98.5(12)
C(17')-C(13')-H(13')	117.6
C(12')-C(13')-H(13')	117.6
C(14')-C(13')-H(13')	117.6
C(15')-C(14')-C(13')	106.3(9)
C(15')-C(14')-H(14C)	110.5
C(13')-C(14')-H(14C)	110.5
C(15')-C(14')-H(14D)	110.5
C(13')-C(14')-H(14D)	110.5
H(14C)-C(14')-H(14D)	108.7
C(14')-C(15')-C(16')	104.6(9)
C(14')-C(15')-H(15C)	110.8
C(16')-C(15')-H(15C)	110.8
C(14')-C(15')-H(15D)	110.8
C(16')-C(15')-H(15D)	110.8
H(15C)-C(15')-H(15D)	108.9
C(15')-C(16')-C(17')	97.5(10)
C(15')-C(16')-C(11')	100.0(12)

C(17')-C(16')-C(11')	99.9(10)
C(15')-C(16')-H(16')	118.5
C(17')-C(16')-H(16')	118.5
C(11')-C(16')-H(16')	118.5
C(13')-C(17')-C(16')	100.3(9)
C(13')-C(17')-H(17C)	111.7
C(16')-C(17')-H(17C)	111.7
C(13')-C(17')-H(17D)	111.7
C(16')-C(17')-H(17D)	111.7
H(17C)-C(17')-H(17D)	109.5
N(2)-C(18)-N(3)	107.5(3)
N(2)-C(18)-C(19)	127.4(3)
N(3)-C(18)-C(19)	125.0(3)
N(2)-C(18)-Al(1)	53.66(15)
N(3)-C(18)-Al(1)	53.89(15)
C(19)-C(18)-Al(1)	178.0(2)
C(18)-C(19)-H(19A)	109.5
C(18)-C(19)-H(19B)	109.5
H(19A)-C(19)-H(19B)	109.5
C(18)-C(19)-H(19C)	109.5
H(19A)-C(19)-H(19C)	109.5
H(19B)-C(19)-H(19C)	109.5
C(21)-C(20)-C(25)	120.8(3)
C(21)-C(20)-N(3)	120.0(3)
C(25)-C(20)-N(3)	119.2(3)
C(22)-C(21)-C(20)	118.2(3)
C(22)-C(21)-C(26)	120.6(3)
C(20)-C(21)-C(26)	121.2(3)
C(23)-C(22)-C(21)	121.7(3)
C(23)-C(22)-H(22)	119.2
C(21)-C(22)-H(22)	119.2
C(24)-C(23)-C(22)	119.5(3)
C(24)-C(23)-H(23)	120.2
C(22)-C(23)-H(23)	120.2
C(23)-C(24)-C(25)	121.8(4)
C(23)-C(24)-H(24)	119.1
C(25)-C(24)-H(24)	119.1
C(21)-C(26)-C(28)	110.6(3)
C(21)-C(26)-C(27)	112.9(3)
C(28)-C(26)-C(27)	110.3(3)
C(21)-C(26)-H(26)	107.6
C(28)-C(26)-H(26)	107.6
C(27)-C(26)-H(26)	107.6
C(26)-C(27)-H(27A)	109.5
C(26)-C(27)-H(27B)	109.5
H(27A)-C(27)-H(27B)	109.5
C(26)-C(27)-H(27C)	109.5
H(27A)-C(27)-H(27C)	109.5
H(27B)-C(27)-H(27C)	109.5
C(26)-C(28)-H(28A)	109.5
C(26)-C(28)-H(28B)	109.5
H(28A)-C(28)-H(28B)	109.5
C(26)-C(28)-H(28C)	109.5
H(28A)-C(28)-H(28C)	109.5
H(28B)-C(28)-H(28C)	109.5
C(24)-C(25)-C(20)	118.0(3)
C(24)-C(25)-C(29)	119.6(7)

C(20)-C(25)-C(29)	122.3(7)
C(24)-C(25)-C(29')	120.3(12)
C(20)-C(25)-C(29')	120.6(12)
C(25)-C(29)-C(30)	110.0(9)
C(25)-C(29)-C(31)	111.8(8)
C(30)-C(29)-C(31)	111.9(8)
C(25)-C(29)-H(29)	107.7
C(30)-C(29)-H(29)	107.7
C(31)-C(29)-H(29)	107.7
C(29)-C(30)-H(30A)	109.5
C(29)-C(30)-H(30B)	109.5
H(30A)-C(30)-H(30B)	109.5
C(29)-C(30)-H(30C)	109.5
H(30A)-C(30)-H(30C)	109.5
H(30B)-C(30)-H(30C)	109.5
C(29)-C(31)-H(31A)	109.5
C(29)-C(31)-H(31B)	109.5
H(31A)-C(31)-H(31B)	109.5
C(29)-C(31)-H(31C)	109.5
H(31A)-C(31)-H(31C)	109.5
H(31B)-C(31)-H(31C)	109.5
C(25)-C(29')-C(30')	110.8(15)
C(25)-C(29')-C(31')	111.7(16)
C(30')-C(29')-C(31')	111.5(16)
C(25)-C(29')-H(29')	107.5
C(30')-C(29')-H(29')	107.5
C(31')-C(29')-H(29')	107.5
C(29')-C(30')-H(30D)	109.5
C(29')-C(30')-H(30E)	109.5
H(30D)-C(30')-H(30E)	109.5
C(29')-C(30')-H(30F)	109.5
H(30D)-C(30')-H(30F)	109.5
H(30E)-C(30')-H(30F)	109.5
C(29')-C(31')-H(31D)	109.5
C(29')-C(31')-H(31E)	109.5
H(31D)-C(31')-H(31E)	109.5
C(29')-C(31')-H(31F)	109.5
H(31D)-C(31')-H(31F)	109.5
H(31E)-C(31')-H(31F)	109.5
C(37)-C(32)-C(33)	119.5(6)
C(37)-C(32)-H(32)	120.3
C(33)-C(32)-H(32)	120.3
C(34)-C(33)-C(32)	119.6(5)
C(34)-C(33)-H(33)	120.2
C(32)-C(33)-H(33)	120.2
C(33)-C(34)-C(35)	120.2(6)
C(33)-C(34)-H(34)	119.9
C(35)-C(34)-H(34)	119.9
C(36)-C(35)-C(34)	119.8(6)
C(36)-C(35)-H(35)	120.1
C(34)-C(35)-H(35)	120.1
C(37)-C(36)-C(35)	121.1(5)
C(37)-C(36)-H(36)	119.5
C(35)-C(36)-H(36)	119.5
C(36)-C(37)-C(32)	119.9(6)
C(36)-C(37)-H(37)	120.0
C(32)-C(37)-H(37)	120.0

N(2)-Al(1)-N(3)	66.52(11)
N(2)-Al(1)-N(1)	80.92(11)
N(3)-Al(1)-N(1)	125.47(12)
N(2)-Al(1)-C(18)	32.92(10)
N(3)-Al(1)-C(18)	33.61(10)
N(1)-Al(1)-C(18)	104.15(11)
N(2)-Al(1)-I(1)	99.71(9)
N(3)-Al(1)-I(1)	119.10(9)
N(1)-Al(1)-I(1)	108.26(9)
C(18)-Al(1)-I(1)	113.28(8)
N(2)-Al(1)-I(2)	160.79(9)
N(3)-Al(1)-I(2)	98.50(8)
N(1)-Al(1)-I(2)	100.03(8)
C(18)-Al(1)-I(2)	131.00(8)
I(1)-Al(1)-I(2)	98.18(4)
C(18)-N(2)-C(9)	132.9(3)
C(18)-N(2)-Al(1)	93.42(18)
C(9)-N(2)-Al(1)	133.7(2)
C(18)-N(3)-C(20)	122.7(3)
C(18)-N(3)-Al(1)	92.50(18)
C(20)-N(3)-Al(1)	143.8(2)

Symmetry transformations used to generate equivalent atoms:

Table S20. Anisotropic displacement parameters (A^2 x 10^3) for R-057.
The anisotropic displacement factor exponent takes the form:
-2 pi^2 [h^2 a*^2 U11 + ... + 2 h k a* b* U12]

	U11	U22	U33	U23	U13	U12
C(1)	23(1)	27(2)	23(1)	-1(1)	2(1)	1(1)
C(2)	30(2)	32(2)	29(2)	2(1)	8(1)	-2(1)
C(3)	26(2)	41(2)	35(2)	-7(2)	8(1)	-6(1)
C(4)	22(1)	38(2)	26(2)	-3(1)	2(1)	2(1)
C(5)	25(1)	28(2)	21(1)	-2(1)	3(1)	3(1)
C(6)	34(2)	31(2)	26(2)	1(1)	1(1)	9(1)
C(7)	36(2)	30(2)	26(2)	8(1)	3(1)	4(1)
C(8)	30(2)	30(2)	29(2)	7(1)	6(1)	-2(1)
C(9)	25(1)	28(2)	18(1)	1(1)	4(1)	2(1)
C(10)	23(1)	25(2)	22(1)	-1(1)	5(1)	1(1)
N(1)	26(1)	24(1)	35(1)	3(1)	3(1)	0(1)
C(11)	49(2)	39(2)	43(2)	1(2)	11(2)	9(2)
C(12)	56(2)	39(2)	68(3)	-9(2)	12(2)	11(2)
C(13)	78(3)	56(3)	86(3)	-8(2)	30(3)	15(3)
C(14)	81(3)	67(3)	81(3)	-2(3)	44(3)	9(3)
C(15)	75(3)	70(3)	51(3)	1(2)	20(2)	-14(3)
C(16)	55(2)	49(2)	50(2)	-18(2)	12(2)	1(2)
C(17)	87(3)	53(3)	81(3)	-12(2)	7(3)	-11(3)
C(11')	63(4)	50(4)	59(5)	-10(4)	9(4)	13(4)
C(12')	70(4)	57(4)	72(5)	-3(4)	10(5)	5(4)
C(13')	73(4)	59(4)	77(4)	-5(4)	22(4)	13(4)
C(14')	75(4)	59(4)	70(4)	-11(4)	19(4)	5(4)
C(15')	75(4)	63(5)	65(4)	-9(4)	15(4)	-7(5)
C(16')	67(4)	51(4)	66(4)	-10(4)	15(4)	5(4)
C(17')	73(5)	54(4)	78(5)	-9(4)	20(4)	7(4)
C(18)	24(1)	25(2)	25(1)	1(1)	6(1)	2(1)
C(19)	29(2)	47(2)	45(2)	16(2)	12(2)	-1(2)
C(20)	22(1)	32(2)	23(1)	3(1)	4(1)	1(1)
C(21)	25(2)	32(2)	30(2)	5(1)	7(1)	5(1)
C(22)	24(2)	48(2)	43(2)	6(2)	6(1)	6(2)
C(23)	26(2)	50(2)	41(2)	7(2)	-4(1)	-2(2)
C(24)	39(2)	44(2)	34(2)	-3(2)	-1(2)	-10(2)
C(26)	28(2)	37(2)	47(2)	-2(2)	8(2)	7(2)
C(27)	58(3)	39(2)	61(3)	4(2)	23(2)	16(2)
C(28)	81(3)	55(3)	44(2)	-8(2)	20(2)	9(2)
C(25)	28(2)	35(2)	34(2)	-4(1)	6(1)	0(1)
C(29)	36(3)	45(4)	59(4)	-22(3)	14(3)	1(3)
C(30)	80(6)	81(8)	76(5)	-33(6)	40(4)	0(6)
C(31)	61(5)	41(4)	87(7)	-14(5)	8(6)	14(3)
C(29')	40(5)	49(6)	59(6)	-20(6)	10(5)	4(5)
C(30')	55(7)	70(9)	51(6)	-36(7)	20(5)	1(7)
C(31')	63(7)	55(6)	77(10)	-22(7)	23(8)	8(6)
C(32)	63(4)	102(5)	102(5)	-43(4)	28(3)	-9(3)
C(33)	60(3)	177(8)	42(3)	-23(4)	17(2)	-41(4)
C(34)	48(3)	127(6)	69(4)	32(4)	16(3)	-15(3)
C(35)	52(3)	73(4)	85(4)	5(3)	28(3)	-13(3)

C(36)	49(3)	89(4)	52(3)	-4(3)	13(2)	-5(3)
C(37)	58(3)	81(4)	73(3)	-1(3)	5(3)	7(3)
Al(1)	22(1)	26(1)	26(1)	5(1)	2(1)	1(1)
N(2)	21(1)	28(1)	28(1)	8(1)	2(1)	-1(1)
N(3)	19(1)	29(1)	26(1)	3(1)	3(1)	2(1)
I(1)	44(1)	44(1)	44(1)	4(1)	21(1)	9(1)
I(2)	31(1)	37(1)	43(1)	16(1)	0(1)	0(1)

Crystal data and structure refinement of mixture complex AlI_2L/AlMeIL

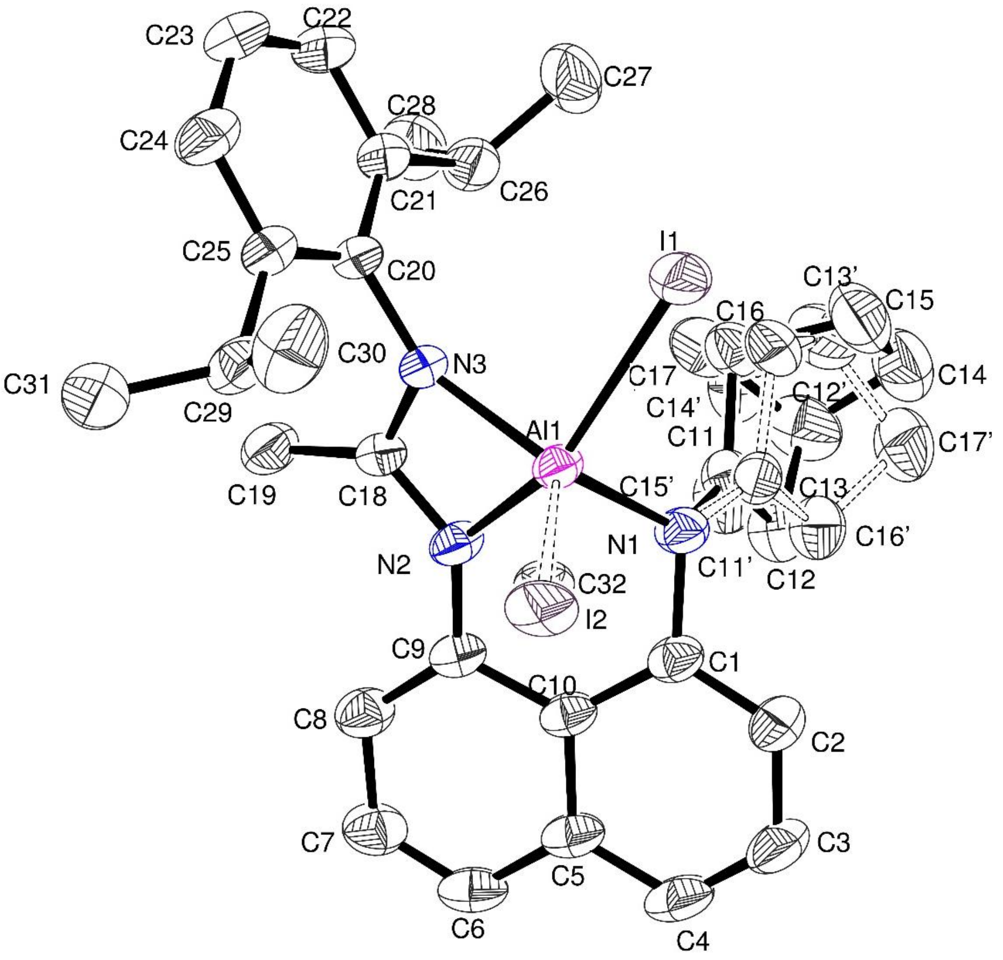

Figure S27 : Asymmetric Unit

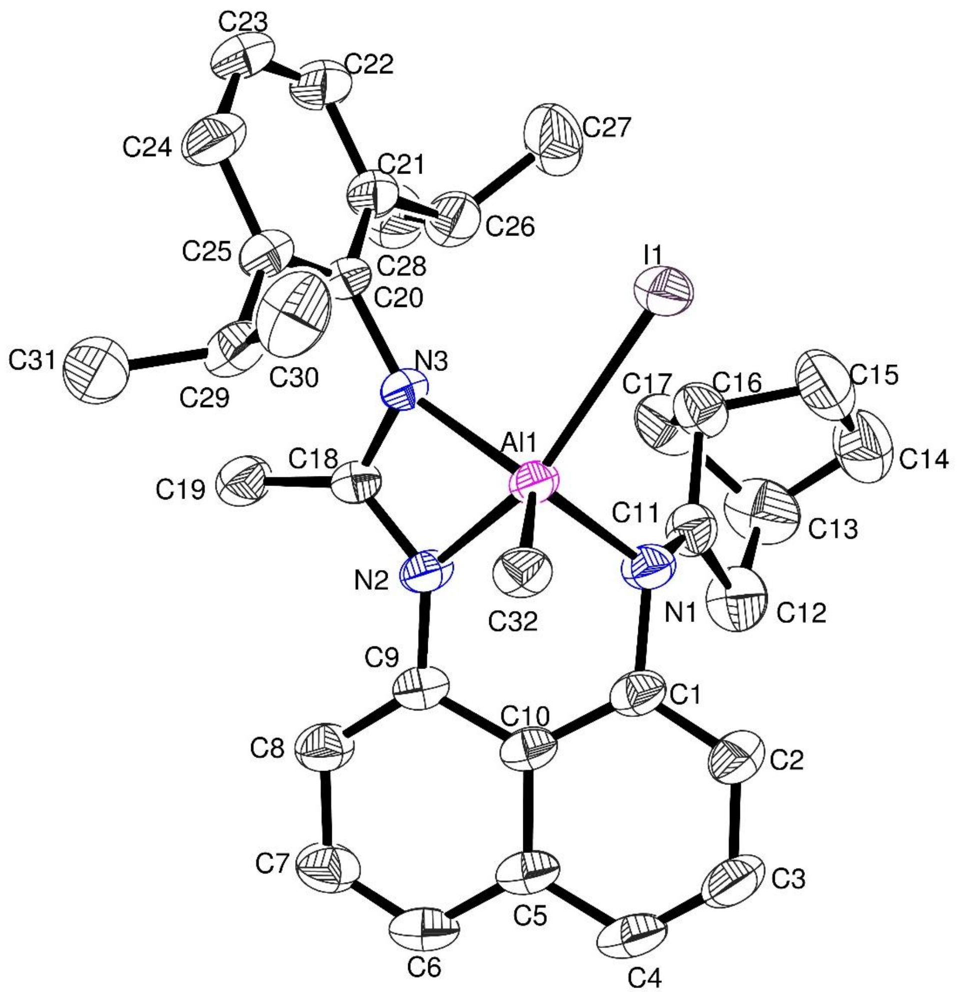

Figure S28 : Compound

Table S21. Crystal data and structure refinement for R-048.

Identification code	R-048
Empirical formula	C31.97 H38.92 Al I1.03 N3
Formula weight	622.45
Temperature	193(2) K
Wavelength	0.71073 A
Crystal system, space group	Monoclinic, P 21/c
Unit cell dimensions	a = 16.3651(13) A alpha = 90 deg.<br>b = 11.1036(9) A beta = 98.214(3) deg.<br>c = 16.7701(15) A gamma = 90 deg.
Volume	3016.1(4) A^3
Z, Calculated density	4, 1.371 Mg/m^3
Absorption coefficient	1.146 mm^-1
F(000)	1277
Crystal size	0.160 x 0.100 x 0.080 mm
Theta range for data collection	2.625 to 32.155 deg.
Limiting indices	-24<=h<=24, -16<=k<=16, -25<=l<=25
Reflections collected / unique	100619 / 10573 [R(int) = 0.0559]
Completeness to theta = 25.242	99.8 %
Refinement method	Full-matrix least-squares on F^2
Data / restraints / parameters	10573 / 301 / 414
Goodness-of-fit on F^2	1.025
Final R indices [I>2sigma(I)]	R1 = 0.0407, wR2 = 0.0921
R indices (all data)	R1 = 0.0767, wR2 = 0.1097
Largest diff. peak and hole	0.603 and -0.598 e.A^-3

Table S22. Atomic coordinates (x 10^4) and equivalent isotropic displacement parameters (A^2 x 10^3) for R-048.
U(eq) is defined as one third of the trace of the orthogonalized Uij tensor.

	x	y	z	U(eq)
C(1)	4407(1)	5502(2)	7891(1)	36(1)
C(2)	5118(2)	4855(3)	7906(2)	47(1)
C(3)	5865(2)	5278(3)	8342(2)	55(1)
C(4)	5882(2)	6325(3)	8755(2)	51(1)
C(5)	5164(1)	7051(3)	8740(2)	41(1)
C(6)	5185(2)	8161(3)	9161(2)	47(1)
C(7)	4494(2)	8841(3)	9126(2)	49(1)
C(8)	3752(2)	8508(3)	8643(2)	44(1)
C(9)	3707(1)	7454(2)	8207(1)	34(1)
C(10)	4412(1)	6659(2)	8274(1)	34(1)
N(1)	3637(1)	4972(2)	7496(1)	37(1)
C(11)	3309(5)	4151(6)	7908(5)	40(1)
C(12)	3660(5)	3630(6)	8721(4)	54(1)
C(13)	2969(5)	2799(6)	8899(4)	71(1)
C(14)	2944(5)	1730(6)	8314(4)	71(2)
C(15)	2659(4)	2226(5)	7567(4)	71(1)
C(16)	2493(4)	3560(5)	7672(3)	44(1)
C(17)	2180(4)	3545(6)	8479(4)	59(1)
C(11')	3441(7)	3923(7)	7752(6)	37(2)
C(12')	2657(5)	3262(7)	7431(5)	50(2)
C(13')	2543(5)	2441(7)	8140(5)	65(2)
C(14')	2461(6)	3248(8)	8884(6)	63(2)
C(15')	3193(6)	3853(8)	9072(5)	73(2)
C(16')	3804(6)	3314(8)	8522(5)	50(2)
C(17')	3487(6)	2077(7)	8448(5)	66(2)
C(18)	2295(1)	7460(2)	7402(1)	31(1)
C(19)	1835(2)	8408(3)	7785(2)	47(1)
C(20)	1192(1)	7063(2)	6298(2)	34(1)
C(21)	513(2)	6347(2)	6434(2)	44(1)
C(22)	-244(2)	6550(3)	5953(2)	57(1)
C(23)	-331(2)	7427(3)	5368(2)	60(1)
C(24)	335(2)	8130(3)	5246(2)	53(1)
C(25)	1110(1)	7962(2)	5706(2)	40(1)
C(26)	586(2)	5374(2)	7078(2)	51(1)
C(27)	373(2)	4133(3)	6713(3)	68(1)
C(28)	59(2)	5657(3)	7740(2)	66(1)
C(29)	1839(2)	8722(3)	5543(2)	46(1)
C(30)	2114(2)	8366(3)	4745(2)	68(1)
C(31)	1666(2)	10077(3)	5568(2)	61(1)
C(32)	3904(3)	6847(5)	6098(3)	36(1)
I(2)	4077(8)	7002(15)	5963(10)	47(3)
Al(1)	3059(1)	6076(1)	6643(1)	30(1)
I(1)	2449(1)	4521(1)	5480(1)	48(1)
N(2)	3065(1)	7097(2)	7618(1)	34(1)
N(3)	1989(1)	6832(2)	6746(1)	31(1)

Table S23. Bond lengths [A] and angles [deg] for R-048.

C(1)-C(2)	1.365(4)
C(1)-C(10)	1.436(4)
C(1)-N(1)	1.460(3)
C(2)-C(3)	1.412(4)
C(2)-H(2)	0.9500
C(3)-C(4)	1.351(5)
C(3)-H(3)	0.9500
C(4)-C(5)	1.422(4)
C(4)-H(4)	0.9500
C(5)-C(6)	1.419(4)
C(5)-C(10)	1.428(3)
C(6)-C(7)	1.354(4)
C(6)-H(6)	0.9500
C(7)-C(8)	1.411(4)
C(7)-H(7)	0.9500
C(8)-C(9)	1.377(4)
C(8)-H(8)	0.9500
C(9)-N(2)	1.392(3)
C(9)-C(10)	1.443(3)
N(1)-C(11')	1.297(8)
N(1)-C(11)	1.305(6)
N(1)-Al(1)	2.015(2)
C(11)-C(16)	1.490(9)
C(11)-C(12)	1.517(9)
C(12)-C(13)	1.521(9)
C(12)-H(12A)	0.9900
C(12)-H(12B)	0.9900
C(13)-C(14)	1.537(9)
C(13)-C(17)	1.610(9)
C(13)-H(13)	1.0000
C(14)-C(15)	1.387(9)
C(14)-H(14A)	0.9900
C(14)-H(14B)	0.9900
C(15)-C(16)	1.521(8)
C(15)-H(15A)	0.9900
C(15)-H(15B)	0.9900
C(16)-C(17)	1.514(8)
C(16)-H(16)	1.0000
C(17)-H(17A)	0.9900
C(17)-H(17B)	0.9900
C(11')-C(16')	1.502(12)
C(11')-C(12')	1.510(12)
C(12')-C(13')	1.531(10)
C(12')-H(12C)	0.9900
C(12')-H(12D)	0.9900
C(13')-C(14')	1.557(11)
C(13')-C(17')	1.608(11)
C(13')-H(13')	1.0000
C(14')-C(15')	1.370(11)
C(14')-H(14C)	0.9900
C(14')-H(14D)	0.9900
C(15')-C(16')	1.572(10)
C(15')-H(15C)	0.9900
C(15')-H(15D)	0.9900

C(16')-C(17')	1.467(11)
C(16')-H(16')	1.0000
C(17')-H(17C)	0.9900
C(17')-H(17D)	0.9900
C(18)-N(2)	1.324(3)
C(18)-N(3)	1.338(3)
C(18)-C(19)	1.492(3)
C(18)-Al(1)	2.448(2)
C(19)-H(19A)	0.9800
C(19)-H(19B)	0.9800
C(19)-H(19C)	0.9800
C(20)-C(25)	1.400(4)
C(20)-C(21)	1.410(3)
C(20)-N(3)	1.433(3)
C(21)-C(22)	1.398(4)
C(21)-C(26)	1.519(4)
C(22)-C(23)	1.376(5)
C(22)-H(22)	0.9500
C(23)-C(24)	1.379(4)
C(23)-H(23)	0.9500
C(24)-C(25)	1.399(3)
C(24)-H(24)	0.9500
C(25)-C(29)	1.519(4)
C(26)-C(27)	1.528(4)
C(26)-C(28)	1.533(5)
C(26)-H(26)	1.0000
C(27)-H(27A)	0.9800
C(27)-H(27B)	0.9800
C(27)-H(27C)	0.9800
C(28)-H(28A)	0.9800
C(28)-H(28B)	0.9800
C(28)-H(28C)	0.9800
C(29)-C(30)	1.524(4)
C(29)-C(31)	1.532(5)
C(29)-H(29)	1.0000
C(30)-H(30A)	0.9800
C(30)-H(30B)	0.9800
C(30)-H(30C)	0.9800
C(31)-H(31A)	0.9800
C(31)-H(31B)	0.9800
C(31)-H(31C)	0.9800
C(32)-Al(1)	1.961(3)
C(32)-H(32A)	0.9800
C(32)-H(32B)	0.9800
C(32)-H(32C)	0.9800
I(2)-Al(1)	2.382(8)
Al(1)-N(3)	1.9704(19)
Al(1)-N(2)	1.989(2)
Al(1)-I(1)	2.6873(7)
C(2)-C(1)-C(10)	120.9(2)
C(2)-C(1)-N(1)	118.3(2)
C(10)-C(1)-N(1)	120.8(2)
C(1)-C(2)-C(3)	120.4(3)
C(1)-C(2)-H(2)	119.8
C(3)-C(2)-H(2)	119.8
C(4)-C(3)-C(2)	120.3(3)

C(4)-C(3)-H(3)	119.9
C(2)-C(3)-H(3)	119.9
C(3)-C(4)-C(5)	121.5(2)
C(3)-C(4)-H(4)	119.2
C(5)-C(4)-H(4)	119.2
C(6)-C(5)-C(4)	121.5(2)
C(6)-C(5)-C(10)	119.7(2)
C(4)-C(5)-C(10)	118.8(3)
C(7)-C(6)-C(5)	120.2(2)
C(7)-C(6)-H(6)	119.9
C(5)-C(6)-H(6)	119.9
C(6)-C(7)-C(8)	121.6(3)
C(6)-C(7)-H(7)	119.2
C(8)-C(7)-H(7)	119.2
C(9)-C(8)-C(7)	120.3(3)
C(9)-C(8)-H(8)	119.9
C(7)-C(8)-H(8)	119.9
C(8)-C(9)-N(2)	126.5(2)
C(8)-C(9)-C(10)	119.8(2)
N(2)-C(9)-C(10)	113.3(2)
C(5)-C(10)-C(1)	117.8(2)
C(5)-C(10)-C(9)	118.2(2)
C(1)-C(10)-C(9)	124.0(2)
C(11')-N(1)-C(1)	116.7(5)
C(11)-N(1)-C(1)	115.8(4)
C(11')-N(1)-Al(1)	131.9(5)
C(11)-N(1)-Al(1)	127.9(4)
C(1)-N(1)-Al(1)	111.38(15)
N(1)-C(11)-C(16)	126.0(7)
N(1)-C(11)-C(12)	127.7(7)
C(16)-C(11)-C(12)	106.3(5)
C(11)-C(12)-C(13)	102.2(5)
C(11)-C(12)-H(12A)	111.3
C(13)-C(12)-H(12A)	111.3
C(11)-C(12)-H(12B)	111.3
C(13)-C(12)-H(12B)	111.3
H(12A)-C(12)-H(12B)	109.2
C(12)-C(13)-C(14)	107.2(6)
C(12)-C(13)-C(17)	100.0(5)
C(14)-C(13)-C(17)	99.8(5)
C(12)-C(13)-H(13)	115.8
C(14)-C(13)-H(13)	115.8
C(17)-C(13)-H(13)	115.8
C(15)-C(14)-C(13)	104.2(5)
C(15)-C(14)-H(14A)	110.9
C(13)-C(14)-H(14A)	110.9
C(15)-C(14)-H(14B)	110.9
C(13)-C(14)-H(14B)	110.9
H(14A)-C(14)-H(14B)	108.9
C(14)-C(15)-C(16)	109.0(6)
C(14)-C(15)-H(15A)	109.9
C(16)-C(15)-H(15A)	109.9
C(14)-C(15)-H(15B)	109.9
C(16)-C(15)-H(15B)	109.9
H(15A)-C(15)-H(15B)	108.3
C(11)-C(16)-C(17)	99.9(5)
C(11)-C(16)-C(15)	106.9(5)

C(17)-C(16)-C(15)	100.5(5)
C(11)-C(16)-H(16)	115.8
C(17)-C(16)-H(16)	115.8
C(15)-C(16)-H(16)	115.8
C(16)-C(17)-C(13)	92.6(4)
C(16)-C(17)-H(17A)	113.2
C(13)-C(17)-H(17A)	113.2
C(16)-C(17)-H(17B)	113.2
C(13)-C(17)-H(17B)	113.2
H(17A)-C(17)-H(17B)	110.5
N(1)-C(11')-C(16')	127.0(8)
N(1)-C(11')-C(12')	123.8(8)
C(16')-C(11')-C(12')	107.4(6)
C(11')-C(12')-C(13')	101.8(6)
C(11')-C(12')-H(12C)	111.4
C(13')-C(12')-H(12C)	111.4
C(11')-C(12')-H(12D)	111.4
C(13')-C(12')-H(12D)	111.4
H(12C)-C(12')-H(12D)	109.3
C(12')-C(13')-C(14')	108.3(7)
C(12')-C(13')-C(17')	100.4(6)
C(14')-C(13')-C(17')	94.4(6)
C(12')-C(13')-H(13')	116.8
C(14')-C(13')-H(13')	116.8
C(17')-C(13')-H(13')	116.8
C(15')-C(14')-C(13')	107.1(7)
C(15')-C(14')-H(14C)	110.3
C(13')-C(14')-H(14C)	110.3
C(15')-C(14')-H(14D)	110.3
C(13')-C(14')-H(14D)	110.3
H(14C)-C(14')-H(14D)	108.5
C(14')-C(15')-C(16')	106.6(7)
C(14')-C(15')-H(15C)	110.4
C(16')-C(15')-H(15C)	110.4
C(14')-C(15')-H(15D)	110.4
C(16')-C(15')-H(15D)	110.4
H(15C)-C(15')-H(15D)	108.6
C(17')-C(16')-C(11')	104.9(7)
C(17')-C(16')-C(15')	99.1(7)
C(11')-C(16')-C(15')	97.2(7)
C(17')-C(16')-H(16')	117.4
C(11')-C(16')-H(16')	117.4
C(15')-C(16')-H(16')	117.4
C(16')-C(17')-C(13')	96.0(6)
C(16')-C(17')-H(17C)	112.6
C(13')-C(17')-H(17C)	112.6
C(16')-C(17')-H(17D)	112.6
C(13')-C(17')-H(17D)	112.6
H(17C)-C(17')-H(17D)	110.1
N(2)-C(18)-N(3)	107.3(2)
N(2)-C(18)-C(19)	128.0(2)
N(3)-C(18)-C(19)	124.7(2)
N(2)-C(18)-Al(1)	54.21(12)
N(3)-C(18)-Al(1)	53.44(11)
C(19)-C(18)-Al(1)	173.16(19)
C(18)-C(19)-H(19A)	109.5
C(18)-C(19)-H(19B)	109.5

H(19A)-C(19)-H(19B)	109.5
C(18)-C(19)-H(19C)	109.5
H(19A)-C(19)-H(19C)	109.5
H(19B)-C(19)-H(19C)	109.5
C(25)-C(20)-C(21)	121.2(2)
C(25)-C(20)-N(3)	119.1(2)
C(21)-C(20)-N(3)	119.6(2)
C(22)-C(21)-C(20)	118.0(3)
C(22)-C(21)-C(26)	119.8(2)
C(20)-C(21)-C(26)	122.2(2)
C(23)-C(22)-C(21)	121.3(3)
C(23)-C(22)-H(22)	119.4
C(21)-C(22)-H(22)	119.4
C(22)-C(23)-C(24)	120.2(2)
C(22)-C(23)-H(23)	119.9
C(24)-C(23)-H(23)	119.9
C(23)-C(24)-C(25)	121.1(3)
C(23)-C(24)-H(24)	119.5
C(25)-C(24)-H(24)	119.5
C(24)-C(25)-C(20)	118.3(2)
C(24)-C(25)-C(29)	120.0(2)
C(20)-C(25)-C(29)	121.7(2)
C(21)-C(26)-C(27)	111.5(3)
C(21)-C(26)-C(28)	111.9(2)
C(27)-C(26)-C(28)	110.8(3)
C(21)-C(26)-H(26)	107.4
C(27)-C(26)-H(26)	107.4
C(28)-C(26)-H(26)	107.4
C(26)-C(27)-H(27A)	109.5
C(26)-C(27)-H(27B)	109.5
H(27A)-C(27)-H(27B)	109.5
C(26)-C(27)-H(27C)	109.5
H(27A)-C(27)-H(27C)	109.5
H(27B)-C(27)-H(27C)	109.5
C(26)-C(28)-H(28A)	109.5
C(26)-C(28)-H(28B)	109.5
H(28A)-C(28)-H(28B)	109.5
C(26)-C(28)-H(28C)	109.5
H(28A)-C(28)-H(28C)	109.5
H(28B)-C(28)-H(28C)	109.5
C(25)-C(29)-C(30)	110.6(3)
C(25)-C(29)-C(31)	112.8(2)
C(30)-C(29)-C(31)	110.9(3)
C(25)-C(29)-H(29)	107.4
C(30)-C(29)-H(29)	107.4
C(31)-C(29)-H(29)	107.4
C(29)-C(30)-H(30A)	109.5
C(29)-C(30)-H(30B)	109.5
H(30A)-C(30)-H(30B)	109.5
C(29)-C(30)-H(30C)	109.5
H(30A)-C(30)-H(30C)	109.5
H(30B)-C(30)-H(30C)	109.5
C(29)-C(31)-H(31A)	109.5
C(29)-C(31)-H(31B)	109.5
H(31A)-C(31)-H(31B)	109.5
C(29)-C(31)-H(31C)	109.5
H(31A)-C(31)-H(31C)	109.5

H(31B)-C(31)-H(31C)	109.5
Al(1)-C(32)-H(32A)	109.5
Al(1)-C(32)-H(32B)	109.5
H(32A)-C(32)-H(32B)	109.5
Al(1)-C(32)-H(32C)	109.5
H(32A)-C(32)-H(32C)	109.5
H(32B)-C(32)-H(32C)	109.5
C(32)-Al(1)-N(3)	123.33(17)
C(32)-Al(1)-N(2)	102.31(18)
N(3)-Al(1)-N(2)	65.54(8)
C(32)-Al(1)-N(1)	107.78(17)
N(3)-Al(1)-N(1)	122.02(9)
N(2)-Al(1)-N(1)	79.39(9)
N(3)-Al(1)-I(2)	123.3(4)
N(2)-Al(1)-I(2)	103.0(4)
N(1)-Al(1)-I(2)	108.2(4)
C(32)-Al(1)-C(18)	114.52(18)
N(3)-Al(1)-C(18)	33.05(7)
N(2)-Al(1)-C(18)	32.67(7)
N(1)-Al(1)-C(18)	103.62(9)
I(2)-Al(1)-C(18)	114.9(4)
C(32)-Al(1)-I(1)	98.95(17)
N(3)-Al(1)-I(1)	95.44(6)
N(2)-Al(1)-I(1)	157.05(6)
N(1)-Al(1)-I(1)	102.28(7)
I(2)-Al(1)-I(1)	98.2(4)
C(18)-Al(1)-I(1)	128.03(6)
C(18)-N(2)-C(9)	134.5(2)
C(18)-N(2)-Al(1)	93.12(14)
C(9)-N(2)-Al(1)	131.30(16)
C(18)-N(3)-C(20)	122.94(19)
C(18)-N(3)-Al(1)	93.51(13)
C(20)-N(3)-Al(1)	141.65(16)

Symmetry transformations used to generate equivalent atoms:

Table S24. Anisotropic displacement parameters (A^2 x 10^3) for R-048.
The anisotropic displacement factor exponent takes the form:
-2 pi^2 [h^2 a*^2 U11 + ... + 2 h k a* b* U12]

	U11	U22	U33	U23	U13	U12
C(1)	30(1)	42(1)	35(1)	9(1)	-2(1)	-1(1)
C(2)	38(1)	46(2)	54(2)	5(1)	-5(1)	6(1)
C(3)	30(1)	63(2)	67(2)	8(2)	-6(1)	9(1)
C(4)	29(1)	69(2)	51(2)	8(1)	-10(1)	-4(1)
C(5)	29(1)	55(2)	35(1)	7(1)	-3(1)	-6(1)
C(6)	36(1)	65(2)	38(1)	0(1)	-5(1)	-13(1)
C(7)	47(1)	58(2)	39(1)	-11(1)	-2(1)	-7(1)
C(8)	36(1)	54(2)	39(1)	-8(1)	0(1)	0(1)
C(9)	28(1)	45(1)	29(1)	1(1)	2(1)	-3(1)
C(10)	27(1)	45(1)	29(1)	7(1)	0(1)	-2(1)
N(1)	32(1)	37(1)	40(1)	7(1)	-4(1)	-3(1)
C(11)	41(3)	36(3)	41(3)	5(2)	5(2)	0(2)
C(12)	65(3)	50(3)	47(3)	15(2)	10(2)	0(3)
C(13)	85(3)	65(3)	60(3)	20(2)	5(2)	-22(3)
C(14)	84(4)	46(3)	86(3)	1(3)	19(3)	-4(3)
C(15)	80(3)	48(3)	86(3)	-18(3)	19(3)	-7(2)
C(16)	44(2)	41(2)	48(3)	-3(2)	14(2)	-7(2)
C(17)	56(3)	64(3)	62(3)	-19(2)	28(2)	-14(2)
C(11')	37(3)	36(3)	38(3)	-2(3)	3(2)	5(3)
C(12')	52(3)	34(3)	62(3)	2(3)	5(3)	-8(3)
C(13')	76(3)	53(3)	67(3)	10(3)	12(3)	-17(3)
C(14')	72(4)	57(4)	68(4)	10(3)	34(3)	7(3)
C(15')	87(4)	71(4)	60(3)	8(3)	13(3)	10(3)
C(16')	58(3)	46(3)	45(3)	8(3)	7(3)	5(3)
C(17')	84(4)	44(3)	68(3)	15(3)	8(3)	5(3)
C(18)	25(1)	36(1)	32(1)	1(1)	2(1)	-3(1)
C(19)	34(1)	55(2)	52(2)	-12(1)	0(1)	8(1)
C(20)	23(1)	36(1)	42(1)	-4(1)	-2(1)	2(1)
C(21)	29(1)	38(1)	63(2)	-2(1)	1(1)	-2(1)
C(22)	27(1)	53(2)	88(2)	-2(2)	-4(1)	-7(1)
C(23)	32(1)	61(2)	80(2)	-3(2)	-17(1)	5(1)
C(24)	40(1)	59(2)	54(2)	8(1)	-12(1)	6(1)
C(25)	30(1)	42(1)	45(1)	2(1)	-3(1)	3(1)
C(26)	33(1)	42(2)	77(2)	10(1)	7(1)	-6(1)
C(27)	58(2)	44(2)	108(3)	-3(2)	27(2)	-10(2)
C(28)	64(2)	61(2)	75(2)	4(2)	15(2)	-7(2)
C(29)	34(1)	53(2)	49(2)	16(1)	0(1)	4(1)
C(30)	69(2)	73(2)	67(2)	19(2)	21(2)	12(2)
C(31)	50(2)	52(2)	81(2)	6(2)	6(2)	-3(2)
C(32)	26(2)	40(2)	43(2)	6(1)	10(1)	-7(2)
I(2)	34(5)	54(6)	57(6)	8(4)	23(4)	-16(4)
Al(1)	25(1)	34(1)	31(1)	1(1)	-1(1)	1(1)
I(1)	38(1)	52(1)	51(1)	-18(1)	-3(1)	0(1)
N(2)	25(1)	45(1)	31(1)	-4(1)	-1(1)	2(1)
N(3)	24(1)	32(1)	35(1)	1(1)	-1(1)	1(1)

Computational details

All calculations were carried out using density functional theory (DFT)[7,8] as implemented in the Gaussian 16 software[9] package employing the (U)M06-2X[10] hybrid functional. Geometry optimizations and analytical frequency calculations were performed using the 6-31G(d,p) triple-ζ basis set[11,12] for C, N, H, and Al atoms, while the LANL2DZ[13,14] effective core potential was used for iodine. Frequency analyses confirmed that all optimized structures correspond to true minima, as all vibrational frequencies were found to be real (i.e. positive). Subsequently, single-point energy calculations were carried out on the optimized geometries using an augmented basis set: 6-311++G(d,p)[15–17] for C, N, H, and Al atoms, and aug-cc-pVTZ-PP[18] for iodine. Thermodynamic corrections corresponding to standard conditions (298 K, 1 atm) were taken into account to report reaction pathways in terms of standard Gibbs free energies. Solvent effects were included in all calculations using the polarizable continuum model[19,20] (PCM), with benzene as the solvent, in line with experimental conditions. Due to the structural complexity of KC_8, where potassium atoms are intercalated between graphite layers, making it unsuitable for standard DFT treatments, it was modeled as a potassium atom bearing a single valence electron[21]. In addition, since the proposed mechanisms involve radical species, all calculations were performed under open-shell (unrestricted) conditions.

It is worth noting that the geometry of the final compound AlI_2L was constructed based on the available single-crystal X-ray diffraction data (R-057).

Cartesian coordinates of optimized structures

At the PCM(benzene)-(U)M06-2X/6-311++G(d,p),aug-cc-pVTZ-PP(I)//(U)M06-2X/6-31G(d,p),LANL2DZ(I) level of theory, E and G correspond to the zero-point and the Gibbs free corrected energies.

LH

E = -1406.889109 Hartree

G = -1406.957339
Hartree

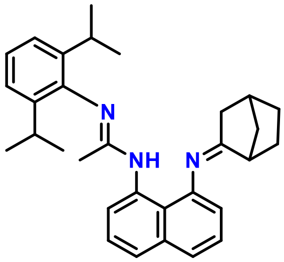

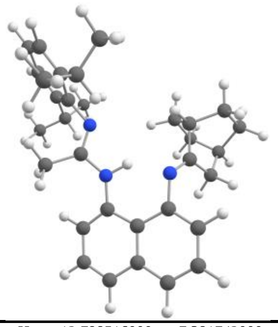

C	9.993830000	6.541927000	3.128314000	C	17.709808000	8.630397000	7.217795000	H	12.798516000	7.381742000	9.209393000
C	8.677968000	6.112926000	3.018551000	C	16.423818000	8.660643000	7.750054000	H	14.116800000	6.388442000	8.560815000
H	8.337544000	5.317996000	3.673798000	H	16.283430000	8.943894000	8.789829000	C	11.111041000	4.687618000	4.096532000
C	7.786454000	6.697536000	2.101040000	C	17.055615000	7.456095000	3.638478000	C	10.719867000	3.638115000	3.062013000
H	6.763085000	6.338331000	2.043798000	H	16.083600000	7.332976000	3.153471000	C	12.030382000	4.131536000	5.161233000
C	8.223444000	7.701246000	1.272090000	C	17.744451000	6.077203000	3.619218000	C	11.617691000	2.398290000	3.261862000
H	7.557737000	8.143244000	0.536409000	H	17.149862000	5.334645000	4.159827000	H	10.818400000	4.062667000	2.055696000
C	9.553451000	8.192818000	1.358062000	H	17.879479000	5.723975000	2.590433000	H	9.665622000	3.358241000	3.174193000
C	9.976473000	9.217626000	0.470853000	H	18.732476000	6.120527000	4.090630000	C	13.371638000	3.767566000	4.470869000
H	9.269333000	9.602158000	-0.257770000	C	17.859423000	8.479926000	2.815537000	H	12.188645000	4.886137000	5.935129000
C	11.264010000	9.688676000	0.526111000	H	18.871244000	8.613961000	3.212961000	C	11.404892000	2.842356000	5.743625000
C	12.163877000	9.204307000	1.493206000	H	17.957503000	8.146525000	1.776307000	C	13.095848000	2.841718000	3.250269000
H	13.175639000	9.582961000	1.494655000	H	17.375478000	9.461981000	2.813071000	H	11.431861000	1.679123000	2.456411000
C	11.782343000	8.251053000	2.432729000	C	15.306544000	8.329674000	6.976700000	C	11.287064000	1.762085000	4.628138000
C	10.462061000	7.654186000	2.335961000	N	12.629277000	7.829939000	3.454603000	H	14.006938000	3.263329000	5.208134000
N	10.836539000	5.940436000	4.090068000	N	14.379302000	7.576863000	4.852461000	H	13.895056000	4.682497000	4.179231000
C	13.764640000	8.366777000	4.037384000	H	18.565512000	8.890794000	7.834027000	H	12.033073000	2.487465000	6.567509000
C	14.158835000	9.802340000	3.770112000	C	13.914130000	8.342976000	7.601487000	H	10.423688000	3.068391000	6.175720000
H	13.283478000	10.435302000	3.614016000	H	13.218912000	7.957847000	6.850469000	H	13.748533000	1.962301000	3.273928000
H	14.733142000	10.173851000	4.620320000	C	13.463003000	9.770099000	7.966700000	H	13.311988000	3.365676000	2.311673000
H	14.799487000	9.883617000	2.885266000	C	13.823790000	7.409774000	8.823395000	H	11.974942000	0.930412000	4.817483000
C	15.495818000	7.981700000	5.614854000	H	13.475340000	10.429311000	7.092632000	H	10.278590000	1.333804000	4.609667000
C	16.803830000	7.921223000	5.070533000	H	12.444144000	9.764251000	8.370168000	H	11.603638000	10.445604000	-0.174949000
C	17.888600000	8.251479000	5.890627000	H	14.119230000	10.213513000	8.724075000	H	12.289434000	7.015690000	3.969441000
H	18.894162000	8.209065000	5.480525000	H	14.473915000	7.742918000	9.639789000				

AlI_3

E = -1130.055943
Hartree

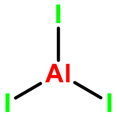

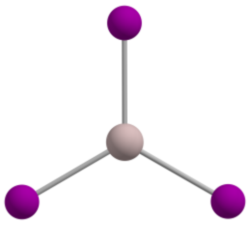

G = -1130.092853
Hartree

Al	12.043471000	6.742826000	5.760442000	I	12.866936000	4.850786000	7.135516000	I	13.044597000	7.186561000	3.535674000
I	10.218854000	8.191124000	6.610125000								

K (radical)

E = -599.929614
Hartree

·K

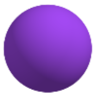

G = -599.945444
Hartree

K	12.043464000	6.742824000	5.760439000

KH

E = -600.520408
Hartree

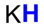

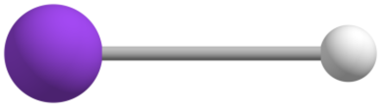

G = -600.539538
Hartree

K	11.933719000	6.829936000	5.811546000
H	10.254772000	8.162616000	6.593401000

L (radical)

E = -1406.271394
Hartree

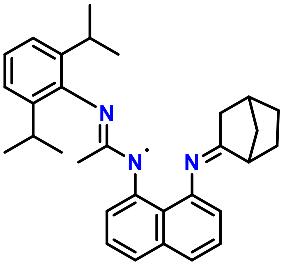

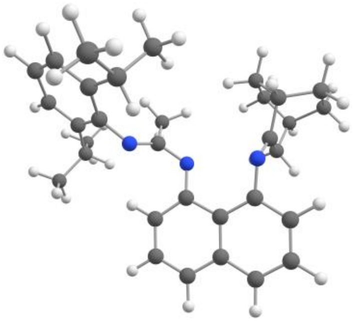

G = -1406.339586
Hartree

C	10.175824000	5.399567000	3.802317000	C	17.560399000	9.168148000	7.379950000	H	13.683208000	12.538681000	5.482221000
C	8.983036000	4.691074000	3.583993000	C	16.874679000	10.062219000	6.564882000	H	13.597534000	11.335054000	6.785009000
H	9.059443000	3.649760000	3.287145000	H	17.283531000	11.056215000	6.406295000	C	11.909000000	4.262929000	2.673075000
C	7.728499000	5.274643000	3.780211000	C	15.218708000	6.132658000	7.301332000	C	11.537071000	4.685135000	1.257874000
H	6.830445000	4.692539000	3.595254000	H	14.141326000	6.222262000	7.123895000	C	13.053898000	3.274897000	2.704209000
C	7.639489000	6.575767000	4.234606000	C	15.416586000	5.643788000	8.745300000	C	12.428438000	3.926187000	0.256224000
H	6.674263000	7.035131000	4.425632000	H	15.061762000	6.383134000	9.469920000	H	11.680509000	5.771071000	1.177401000
C	8.813952000	7.324577000	4.478077000	H	14.857317000	4.715982000	8.906332000	H	10.473390000	4.497054000	1.069775000
C	8.702135000	8.646690000	4.996776000	H	16.467251000	5.428746000	8.970482000	C	14.282721000	3.876594000	1.984444000
H	7.709154000	9.050715000	5.171937000	C	15.762553000	5.094426000	6.299026000	H	13.287443000	3.026666000	3.742420000
C	9.828550000	9.400433000	5.284693000	H	16.846108000	4.972064000	6.411928000	C	12.594982000	2.018216000	1.920588000
C	11.097129000	8.889924000	5.032679000	H	15.292498000	4.117401000	6.458736000	C	13.903888000	4.283722000	0.529462000
H	11.971673000	9.495063000	5.225868000	H	15.566318000	5.396231000	5.265702000	H	12.159219000	4.207723000	-0.768138000
C	11.289935000	7.594873000	4.455755000	C	15.666794000	9.714885000	5.946836000	C	12.226182000	2.409356000	0.456950000
C	10.108826000	6.759164000	4.229338000	N	12.478347000	7.176197000	4.017645000	H	15.084774000	3.129839000	1.983690000
N	11.360416000	4.656027000	3.752967000	N	13.888212000	8.013681000	5.671475000	H	14.657201000	4.734008000	2.550585000
C	13.678012000	7.713578000	4.428203000	H	18.500929000	9.457742000	7.839661000	H	13.406236000	1.281781000	1.935371000
C	14.708436000	7.792238000	3.318847000	C	14.914957000	10.764831000	5.132234000	H	11.743060000	1.555387000	2.429601000
H	14.495563000	8.655181000	2.676758000	H	14.102527000	10.262875000	4.599911000	H	14.546949000	3.773852000	-0.196590000
H	15.719352000	7.900570000	3.715438000	C	15.794953000	11.447117000	4.069222000	H	14.053803000	5.358905000	0.377416000
H	14.646278000	6.902773000	2.689019000	C	14.265111000	11.811469000	6.060300000	H	12.845453000	1.858665000	-0.259813000
C	15.141175000	8.416554000	6.173091000	H	16.258536000	10.713693000	3.402230000	H	11.185810000	2.143682000	0.236931000
C	15.811930000	7.512892000	7.041438000	H	15.194854000	12.131018000	3.458910000	H	9.724177000	10.402850000	5.689454000

C	17.023313000	7.903689000	7.614979000	H	16.598188000	12.037040000	4.522859000
H	17.553195000	7.213366000	8.264150000	H	15.026231000	12.362013000	6.623886000

AlI_2 (radical)

E = -834.155371 Hartree

G = -834.188256 Hartree

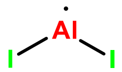

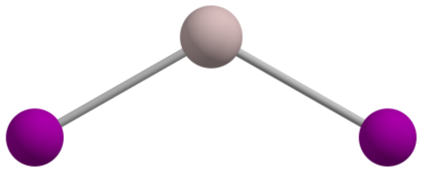

Al	12.032701000	6.738051000	5.784362000	I	10.168613000	8.229150000	6.636158000	I	12.887436000	4.799582000	7.175579000

KI

E = -895.859882 Hartree

G = -895.885372 Hartree

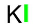

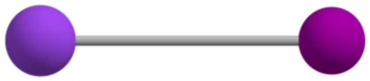

K	12.247671000	6.580733000	5.665344000
I	9.940820000	8.411819000	6.739603000

LK

E = -2006.313765 Hartree

G = -2006.383340 Hartree

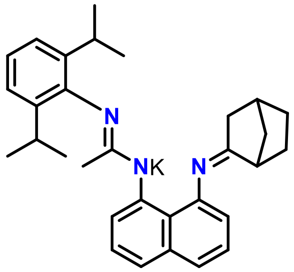

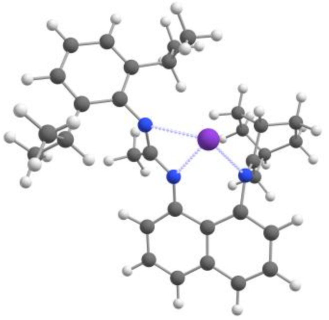

C	10.615741000	5.600644000	2.890081000	C	17.758868000	9.338758000	7.216101000	H	12.606235000	10.693048000	8.509365000
C	9.563857000	4.774933000	2.514143000	C	16.565332000	10.051089000	7.286445000	H	13.418439000	9.119176000	8.624373000
H	9.693096000	3.699908000	2.602968000	H	16.554006000	11.015965000	7.786526000	C	12.600779000	4.479380000	2.430953000
C	8.371660000	5.303601000	1.983461000	C	16.631900000	6.159951000	5.411478000	C	12.473487000	4.672626000	0.923365000
H	7.568495000	4.632640000	1.691696000	H	15.766082000	6.099554000	4.744833000	C	13.823004000	3.665554000	2.795343000
C	8.252184000	6.660051000	1.805465000	C	16.452283000	5.077045000	6.495914000	C	13.597863000	3.896441000	0.211924000
H	7.355329000	7.083949000	1.361910000	H	15.551677000	5.262513000	7.093161000	H	12.523151000	5.745481000	0.695672000
C	9.287039000	7.546081000	2.210092000	H	16.380000000	4.075117000	6.053770000	H	11.486194000	4.338817000	0.587193000
C	9.128880000	8.942942000	2.015708000	H	17.300197000	5.076112000	7.190002000	C	15.076120000	4.363031000	2.211271000
H	8.226604000	9.306801000	1.533169000	C	17.886366000	5.874845000	4.569911000	H	13.902721000	3.574213000	3.884328000
C	10.100831000	9.810679000	2.457731000	H	18.793299000	5.833860000	5.182586000	C	13.683557000	2.264427000	2.148635000
C	11.234509000	9.338016000	3.137275000	H	17.796524000	4.906808000	4.064269000	C	14.961489000	4.455987000	0.661127000
H	11.917481000	10.058227000	3.571957000	H	18.035672000	6.644125000	3.805964000	H	13.488034000	4.001831000	-0.873074000
C	11.461813000	7.971336000	3.366440000	C	15.378003000	9.551856000	6.739905000	C	13.503337000	2.408494000	0.607675000
C	10.484818000	7.030004000	2.826725000	N	12.431939000	7.493274000	4.205354000	H	15.964310000	3.792537000	2.504592000
N	11.805845000	4.930328000	3.328977000	N	14.195546000	7.697459000	5.632292000	H	15.180514000	5.358117000	2.653089000
C	13.599382000	8.081307000	4.506514000	H	18.669573000	9.746945000	7.645094000	H	14.581410000	1.681763000	2.384471000
C	14.297307000	9.047835000	3.551001000	C	14.080550000	10.334400000	6.931853000	H	12.836608000	1.731886000	2.594082000
H	14.268291000	10.076887000	3.923114000	H	13.325080000	9.894822000	6.276808000	H	15.767755000	3.893528000	0.177161000
H	15.353858000	8.776794000	3.467113000	C	14.204756000	11.822829000	6.560189000	H	15.063364000	5.495472000	0.330899000
H	13.835074000	9.031938000	2.562955000	C	13.563652000	10.175707000	8.376152000	H	14.267368000	1.833329000	0.073361000

C	15.390696000	8.285288000	6.090451000	H	14.584012000	11.952613000	5.541321000	H	12.533275000	2.005895000	0.294918000
C	16.608091000	7.551651000	6.033816000	H	13.227500000	12.314313000	6.623121000	H	9.977374000	10.882330000	2.320817000
C	17.768327000	8.092098000	6.596856000	H	14.881337000	12.359682000	7.234020000	K	12.517207000	5.585717000	5.978025000
H	18.697438000	7.530890000	6.549909000	H	14.275710000	10.591923000	9.097748000				

AlI_2L

E = -2240.579822 Hartree

G = -2240.652104 Hartree

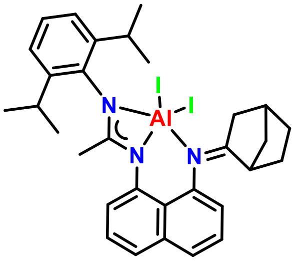

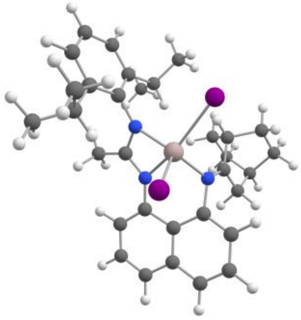

C	10.132014000	6.220922000	3.648691000	C	16.395354000	9.334900000	7.801109000	H	14.326252000	10.135420000	9.620111000
C	8.912280000	5.584634000	3.756650000	H	16.440636000	10.133047000	8.536098000	H	12.641157000	10.371541000	9.126543000
H	8.839846000	4.674362000	4.343002000	C	16.291306000	6.117536000	4.908754000	H	13.332011000	8.744123000	9.154988000
C	7.777431000	6.093786000	3.091625000	H	15.266778000	6.001963000	4.544969000	C	11.761940000	4.537849000	3.573295000
H	6.826267000	5.580292000	3.189818000	C	16.704841000	4.780830000	5.552352000	C	11.120619000	3.942057000	2.326966000
C	7.890819000	7.209754000	2.296813000	H	16.054339000	4.533634000	6.395384000	C	13.033952000	3.826258000	3.944113000
H	7.034582000	7.581304000	1.741284000	H	16.644071000	3.968239000	4.819119000	C	11.975206000	2.770820000	1.809492000
C	9.116918000	7.920756000	2.207233000	H	17.736960000	4.811184000	5.916971000	H	11.007777000	4.729666000	1.570880000
C	9.232426000	9.087470000	1.405341000	C	17.181981000	6.438431000	3.691882000	H	10.106783000	3.600889000	2.554597000
H	8.375097000	9.411166000	0.823114000	H	18.229405000	6.559231000	3.988370000	C	14.000966000	3.949527000	2.733030000
C	10.406929000	9.800667000	1.389282000	H	17.137486000	5.628662000	2.954859000	H	13.481366000	4.257192000	4.837728000
C	11.493848000	9.431036000	2.209222000	H	16.872055000	7.363894000	3.195620000	C	12.717650000	2.328474000	4.185243000
H	12.355190000	10.083350000	2.248839000	C	15.190135000	9.096864000	7.129539000	C	13.383990000	3.289087000	1.468421000
C	11.430847000	8.300744000	3.016239000	Al	12.172525000	6.809858000	5.655900000	H	11.499976000	2.349038000	0.917439000
C	10.252881000	7.466613000	2.953900000	N	12.342533000	7.943804000	3.997453000	C	12.069365000	1.702592000	2.917150000
N	11.312437000	5.577907000	4.204832000	N	13.923935000	7.777882000	5.480474000	H	14.940355000	3.462993000	3.014061000
C	13.554660000	8.383438000	4.344538000	I	10.280000000	8.136565000	6.820731000	H	14.237475000	5.002816000	2.551820000
C	14.394875000	9.388652000	3.612588000	I	12.805009000	5.022846000	7.486289000	H	13.658875000	1.828456000	4.435381000
H	13.988523000	10.394354000	3.765114000	H	18.455042000	8.775931000	8.082623000	H	12.071671000	2.228334000	5.061912000
H	15.419610000	9.374885000	3.984145000	C	13.980539000	9.965205000	7.460731000	H	14.007393000	2.460215000	1.116600000
H	14.388690000	9.183794000	2.538860000	H	13.141578000	9.634855000	6.844949000	H	13.326715000	4.009106000	0.644559000
C	15.147010000	8.051979000	6.174422000	C	14.234565000	11.450082000	7.134852000	H	12.656437000	0.850482000	2.558594000
C	16.291641000	7.258706000	5.920530000	C	13.548283000	9.791580000	8.929169000	H	11.068598000	1.318074000	3.144102000
C	17.471799000	7.543442000	6.617249000	H	14.525253000	11.591454000	6.088177000	H	10.493274000	10.694207000	0.777993000
H	18.358307000	6.945940000	6.425882000	H	13.328921000	12.038087000	7.316823000				
C	17.530335000	8.572421000	7.550303000	H	15.032913000	11.869798000	7.756671000				
